# CD147 mediates the adsorption of influenza A virus on the cell surface through direct interaction with HA

**DOI:** 10.3389/fcimb.2025.1647283

**Published:** 2025-08-29

**Authors:** Ting Wang, Lei Cao, Yufei Zhang, Chuxing Cheng, Haiying Mao, Xiaotong Hu, Xiaomei Sun, Kun Huang, Meilin Jin

**Affiliations:** ^1^ State Key Laboratory of Agricultural Microbiology, Huazhong Agricultural University, Wuhan, China; ^2^ College of Animal Medicine, Huazhong Agricultural University, Wuhan, China; ^3^ Center for Cross-disciplinary in Animal Epidemic and Public Health, Hubei Jiangxia Laboratory, Wuhan, China

**Keywords:** avian influenza virus, hemagglutinin, CD147, viral adsorption, hostpathogen interaction, influenza A virus

## Abstract

The adsorption of avian influenza virus (AIV) initiates the viral lifecycle, determining host tropism and pathogenicity. In addition to the classical AIV receptor, auxiliary receptors also play important roles in viral adsorption, though these remains to be fully elucidated. In this study, we identified 25 avian membrane proteins that interact with H5N6 hemagglutinin (HA), one of which, CD147, was confirmed to play a crucial role in promoting AIV adsorption and replication through overexpression and knockout experiments in DF1 and A549 cells. As a highly glycosylated transmembrane protein, CD147 was further shown to directly bind to the HA receptor-binding domain via its extracellular immunoglobulin-like domains, independently of its glycosylation, thereby mediating viral adsorption. Moreover, AIV infection upregulated the hyperglycosylated form (HG-CD147), while glycosylation inhibitors reduced viral adsorption, highlighting the role of glycosylation in modulating CD147 function. Finally, disrupting the CD147-HA interaction with soluble proteins or monoclonal antibodies inhibited viral adsorption and replication. This study identifies CD147 as an adjuvant receptor that promotes influenza virus adsorption and provides a mechanistic foundation basis for developing broad-spectrum therapeutics targeting HA-host protein interactions.

## 1 Introduction

Birds are the natural reservoir of influenza viruses (Lloren et al., 2017), and the cross-species transmission of avian influenza viruses (AIVs) has emerged as a global biosecurity concern (World Health Organization, 2025; de Jong et al., 2005). The continuous evolution of novel AIV variants has led to an expanding host range (Zou et al., 2025), causing systemic economic losses in livestock industries and posing persistent threats to global public health (World Health Organization, 2025; Zhao et al., 2024; Nooruzzaman et al., 2025). Influenza viruses rely not only on viral proteins but also heavily on host cellular proteins and associated mechanisms to complete their life cycle (Newhouse et al., 2009).

As an enveloped virus, the influenza virus (Ivanova et al., 2015), initiates infection by binding its surface spike glycoprotein, hemagglutinin (HA), to host receptors. HA is a trimer composed of HA1 and HA2 subunits, derived from proteolytic cleavage of HA0, with HA1 responsible for recognizing host cell membrane receptors and triggering endocytosis (Yang et al., 2013). Traditionally, sialic acid (SA) has been regarded as the primary receptor, with its distribution and structural features (e.g., linkage type) directly influencing viral adsorption efficiency and host specificity (Wilson et al., 2002; Imai and Kawaoka, 2012). Regulatory factors such as SLC35A1 (Han et al., 2018) and GNE (Ma et al., 2023) have been extensively studied. However, sialidase-treated MDCK cells (Stray et al., 2000) and SA-deficient Lec8 cells (Kajiwara et al., 2020) remain susceptible to infection, suggesting the involvement of unidentified non-SA receptors in mediating viral adsorption and internalization (Wang et al., 2020). Although recent studies revealed SA-dependent (Eierhoff et al., 2010) and -independent (Ni et al., 2024) entry pathways (Chan et al., 2016), the mechanistic roles of alternative receptors remain poorly understood.

CD147, a single-pass transmembrane N-glycoprotein belonging to the immunoglobulin superfamily (Yoshida et al., 2000), undergoes terminal glycan modifications including fucosylation and sialylation (Bai et al., 2014). It is ubiquitously expressed across epithelial cells (Wu et al., 2016) and collaborates with cytokines to regulate diverse physiological and pathological processes (Kumar et al., 2019). Accumulating evidence indicates that CD147 acts as a receptor or co-receptor by interacting with pathogen surface glycoproteins to facilitate cellular recognition, adhesion, and entry, thereby promoting infections by multiple pathogens. For instance, CD147 mediates Plasmodium falciparum invasion (Zhang et al., 2018), supports Neisseria meningitidis adhesion (dos Santos Souza et al., 2021) and Listeria monocytogenes cell-to-cell spread (Dhanda et al., 2019), and enhances the adsorption and endocytosis of several viruses, including HIV (Pushkarsky et al., 2001), SARS-CoV (Chen et al., 2005), HBV (Tian et al., 2010), HCV (Shackel et al., 2002; Julich-Haertel et al., 2017), KSHV (Dai et al., 2013), and SARS-CoV-2 (Pushkarsky et al., 2001; Wang K. et al., 2021).

Notably, Fu first identified CD147 in clinical samples from patients infected with H1N1 influenza, suggesting its association with influenza virus infection (Fu et al., 2016). Despite this association, the specific molecular role of CD147 in AIV life cycle has not been characterized. No prior study has demonstrated a direct interaction between CD147 and any viral protein. This study aims to investigate the role of CD147 in avian influenza virus infection by determining whether it directly interacts with HA and contributes to viral adsorption and replication. We identified CD147 as a host membrane protein that interacts with AIV HA protein and positively regulates viral proliferation in avian DF1 cells through membrane protein isolation combined with immunoprecipitation-mass spectrometry. Further investigations demonstrated that the extracellular domain of CD147 directly interacts with the receptor-binding domain (RBD) in the globular head region of HA, mediating virus adsorption on the host cell surface. These findings indicate that cell surface proteins beyond SA participate in influenza virus adsorption, highlighting the critical role of CD147 in the virus life cycle.

## 2 Materials and methods

### 2.1 Cells, viruses, and plasmids

DF-1, MDCK, HEK293T, and HeLa cells were cultured in Dulbecco’s modified Eagle Medium (DMEM, Thermo Fisher Scientific, #11965092). A549 cells were cultured in Ham’s F-12 medium (Thermo Fisher Scientific, #11765054). All cell lines were sourced from the American Type Culture Collection. All basic culture media were supplemented with 10% fetal bovine serum (FBS; PAN-Biotech, #P30–3306) and 1% penicillin–streptomycin. All cells were cultured at 37°C in 5% CO2. HEK293F cells were cultured in SMM 293T-II-N medium (Sino Biological, #RZ18JA2801-A) at 37°C in 5% CO2 with constant shaking at 130 rpm.

The influenza A virus (IAV) strains used were A/Puerto Rico/8/34 (H1N1-PR8), A/duck/Hubei/WH18/2015 (H5N6-JX) (GenBank accession number KX652135), and A/chicken/Zhe Jiang/2023010/2023 (H9N2), the latter of which was isolated and pre-served by our laboratory. The HA gene sequences are provided in **Supplementary Material S1**. All viruses were propagated in 10-day-old embryonated eggs and stored in our laboratory.

Human and chicken host genes were cloned into the eukaryotic expression vector p3×Flag-CMV-14, while influenza virus HA genes were inserted into the eukaryotic expression vector pCAGGS-Ha (HA stands for the HA protein of influenza virus, and Ha stands for the Ha tag). The CRISPR/Cas9 vector lentiCRISPR v2 vector, lenti-virus packaging plasmid psPAX2, and membrane protein plasmid pMD2.G were also used. PCR primers were designed using SnapGene v7.2 and synthesized by Tsingke (Beijing, China). All plasmid constructs were verified through sequencing. Small interfering RNAs (siRNAs) targeting chicken CD147 were designed and synthesized by JTS Scientific (Wuhan, China), and knockdown efficiency was validated via western blotting. Primer and siRNA sequences are listed in **Supplementary Table S1**.

### 2.2 Antibodies and reagents

The following antibodies were used: anti-Flag mouse monoclonal antibody (Sigma, Germany, #F1804); anti-CD147 rabbit monoclonal antibody, anti-CD147 mouse poly-clonal antibody, anti-HA rabbit polyclonal antibody, and anti-GAPDH mouse mono-clonal antibody (Wuhan Sanying, #11989-1-AP, #66443-1-Ig, #51064-2-Ig, and #60004-1-Ig, respectively); anti-Strep mouse monoclonal antibody (AbClon, #AE066); and rabbit polyclonal antibodies against IAV proteins HA, NA, M1, M2, and NP (GeneTex, #GTX127357, #GTX125974, #GTX125928, #GTX640415, and #GTX125923, respectively). Secondary antibodies included Alexa Fluor 488-labeled AffiniPure goat anti-mouse IgG and Alexa Fluor 594-labeled AffiniPure goat anti-rabbit IgG (Wuhan Sanying, #SA00013–1 and #SA00013-4, respectively).

The following reagents were used: DAPI (Beyotime, #C1002); ampicillin, kanamy-cin A, puromycin, polybrene, PEI, PD153035 (MedChemExpress, #HY-B0522, #HY-16566, #HY-B1743, #HY-112735, #HY-K2014, and #153436-54-5, respectively); and N-tosyl L-phenylalanine chloromethyl ketone (TPCK) trypsin (2.5μg/mL, Worthington Biotechnology, #LS003740).

### 2.3 Virus manipulation

Upon reaching 90% confluence or following transfection, cells were washed twice with phosphate-buffered saline (PBS), followed by incubation with diluted viruses for 1 h at 37°C in 5% CO2. The viral diluent was then removed and replaced with serum-free medium containing 1% penicillin–streptomycin. All experiments with H5N6-JX viruses were conducted in a biosafety level 3 (BSL3) laboratory, according to the guidelines of the BSL3 laboratory at Huazhong Agricultural University (HZAU). All procedures were approved by the Institutional Biosafety Committee of HZAU.

### 2.4 Transfections and virus titration

Transfections were performed using Lipofectamine 2000 (Invitrogen, #11668500) at a 2:1 Lipofectamine/DNA or RNA ratio in serum-free minimal essential medium (MEM) Opti-MEM (Invitrogen), according to the manufacturer’s protocol. The culture medium was replaced with fresh medium supplemented with 10% FBS 6 h post-transfection. For virus titration of IAV, viral supernatants were harvested at the indicated time points, serially diluted in DMEM, and adsorbed onto MDCK cell monolayers seeded in 96-well plates. After 1 h of adsorption, the inoculum was removed, the cells were washed with PBS, and fresh DMEM, with or without trypsin-TPCK, was added. The plates were incubated at 37°C for 72 h. Virus titers were determined by calculating log10^(50% tissue culture infective dose[TCID50])^/mL using the Spearman–Karber method (Ramakrishnan, 2016).

### 2.5 Co-immunoprecipitation assay

Cells were transfected with the indicated plasmids. Whole-cell lysates from cells were prepared as described previously (Hui et al., 2021). Co-IP was performed using anti-Flag or anti-Ha antibody-conjugated magnetic beads (MedChemExpress, #HY-K0207 and #HY-K0201). Subsequently, the beads were washed three times with lysis buffer and eluted with 1× sodium dodecyl sulfate (SDS) loading buffer. Samples were evaluated using western blotting.

### 2.6 Affinity purification mass spectrometry analysis

To identify HA-associated host factors, we performed AP-MS. DF-1 cells were seeded into six-well plates and transfected with pCAGGS-H5-ACS-HA or pCAGGS-HA empty vectors for 24 h. The cells were then washed with PBS and lysed in non-denaturing protein lysis buffer (Invent, #Minute™ WA-010). The lysates were incubated with HA-labeled agarose beads (MedChemExpress, #HY-K0201) for 4 h while rotating at 4°C. To isolate membrane proteins, DF-1 cells were seeded into 6-well plates and subjected to plasma membrane/protein isolation using a cell fractionation kit (In-vent, #Minute™ SM-005) according to the manufacturer’s protocol. The membrane proteins were then incubated with the HA-labeled agarose beads for 8 h and rotated at 4°C. Bead-bound immune complexes were washed five times and subjected to tryptic digestion. After reduction and alkylation, trypsin (mass ratio, 1:50) was added, and the proteins were hydrolyzed at 37°C for 20 h. After desalination, the enzymatic hydrolysis product was lyophilized and redissolved in 0.1% formic acid solution. The liquid chromatography-tandem mass spectrometry signals were processed using the Mascot 2.2 algorithm, with the Uniprot Gallus protein database (36,624 sequences). The following parameters were used: variable modifications—oxidation (Met), N-acetylation, pyroglutamination (Gln); maximum missed cleavages—2; peptide mass tolerance—100 ppm; MS/MS tolerance—0.5 Da. Protein identification was based on at least one MS/MS data signal with Mascot scores exceeding the threshold (P < 0.05).

### 2.7 Western blotting

Cells were lysed using either the Mammalian Protein Extraction Kit (KWBio, #CW0889M) or Western and IP Cell Lysis Buffer (Beyotime, #P0013J) after being washed twice with PBS. Protein concentrations were quantified using standard procedure. Each protein sample was separated using 10–12% SDS-polyacrylamide gel electrophoresis (PAGE) and transferred onto a polyvinylidene difluoride membrane. The membrane was incubated with 2% (v/w) bovine serum albumin (BSA) for 1 h at room temperature and sequentially incubated with primary and secondary antibodies. Images were captured using an ECL detection system (Tannon, Beijing, China). All experiments were repeated at least three times, and representative results are shown.

### 2.8 Cytotoxicity assay

The viability of DF1 and A549 cells following transfection, treatment with small molecule reagents, or knockout (KO) was assessed using a Cell-Counting Kit-8 (CCK-8) assay, according to the manufacturer’s instructions (MedChemExpress, #HY-K0301) (Hua et al., 2019). Briefly, cells in 96-well plates were treated with siRNAs or compounds. Cell viability was measured at 24 h after treatment, where 10μl of the CCK-8 reagent was added to each well and incubated at 37°C for 2 h. Absorbance of samples was measured at 450 nm. The cell survival rate was calculated using the following formula: [(As-Ab)/(Ac-Ab)] × 100, where As, Ac, and Ab are the absorbance of the test, control, and blank wells.

### 2.9 Generation of CD147-knockout A549 cells

CD147-KO A549 cells were generated using the CRISPR/Cas9 system as previously described (Sander and Joung, 2014). Two single guide RNAs (sgRNAs) targeting the human CD147 gene (sgCD147; the sequences are listed in **Supplementary Table S2**) and a control sgRNA targeting the luciferase gene (sgLuc) were cloned into the lentiCRISPR v2 vector. Recombinant lentiviruses were produced in HEK293T cells by co-transfecting the lentiCRISPR-sgRNA constructs with psPAX2 and pMD2G. A549 cells expressing Cas9 were transduced with the sgCD147 or sgLuc lentiviruses. At 48 h post-infection (hpi), cells were subjected to puromycin selection (2.5μg/ml) to obtain pooled CD147-KO or control clones. Monoclonal cell lines were then established by sorting with flow cytometry and seeding single cells into a 96-well plate to generate clonal lines. CD147 KO in A549 cells was confirmed via sequence (supporting material sequence S2) and western blotting.

### 2.10 Drug inhibition assays

For the CD147 glycosylation inhibition assay, wild-type A549 and MDCK cells were pretreated with PD153035 (MedChemExpress, #153436-54-5) at varying concentrations for 6, 12, and 24 h. After pretreatment, the cells were infected with influenza virus at a multiplicity of infection (MOI) of 10 for virus binding assays. CD147 expression and glycosylation status were assessed via western blotting.

### 2.11 Virus binding and internalization

Virus binding and internalization analyses were performed as previously described (Ni et al., 2024). DF-1 cells in 12-well plates were transfected with expression plasmids or siRNAs and then infected with the H9N2 virus. A549 cells in 12-well plates were transfected with expression plasmids, treated with drugs, or transduced with CD147-KO lentiviruses and then incubated with the PR8 virus. Both virus strains were used at an MOI of 50 or 10 at 4 °C for 1 h. The cells were subsequently washed with ice-cold PBS, and lysates were prepared to measure viral RNA (vRNA) levels via quantitative reverse transcription PCR (RT-qPCR) and protein levels via western blotting.

For the internalization assay, treated cells in 12-well plates were infected with virus at an MOI of 50 or 10 and incubated at 4 °C for 1 h. The cells were then washed three times with cold serum-free DMEM or Ham’s F-12 medium and incubated at 37 °C for 15 or 60 min to allow internalization. The cells were then washed five times with cold acidic PBS (pH 1.3) to remove any cell surface-bound virus. The cells were then lysed to quantify the vRNA levels and protein levels.

### 2.12 RNA isolation and RT-qPCR

Total RNA was extracted using TRIzol reagent, as previously described. RNA was reverse transcribed into cDNA using the AMV XL reverse transcriptase (TaKaRa Bio, #2620A). Real-time PCR was conducted using FastStart Universal SYBR Green Master Mix (Roche, #04913914001) on a Vii7A real-time PCR system (Applied Biosystems). Primer sequences used for RT-qPCR are listed in **Supplementary Tables S1**, **S2**, and **S3**. Relative gene expression levels were calculated using the 2−ΔΔCt method with GAPDH as the internal control.

### 2.13 Virus growth kinetics

DF-1 or A549 cells were washed with DMEM or Ham’s/F-12 medium and infected with the indicated virus at an MOI of 0.001 or 0.01. After 1 h of virus adsorption, the inoculum was removed, and the cells were washed with DMEM or Ham’s/F-12. Infected cells were cultured in MEM. For PR8 virus infections, MDCK cells were maintained in MEM supplemented with 2.5 mg/mL TPCK-trypsin, and A549 cells were maintained in MEM with 1 mg/mL TPCK-trypsin. The supernatants from infected cells were collected at various time points post-infection and added to MDCK cells to determine viral titers. Viral titers were calculated as log10^(TCID50)^/mL using the Spearman–Karber method (Ramakrishnan, 2016).

### 2.14 Indirect immunofluorescence imaging

DF-1 and HeLa cells were cultured on coverslips and transfected with the indicated plasmids using Lipofectamine 2000. A549 cells were infected with PR8 at an MOI of 0.1. After 24 hpi, the cells were washed twice with PBS, fixed with 4% paraformaldehyde for 15 min, permeabilized with 0.5% (v/v) Triton X-100 for 15 min, and blocked with 2% (v/w) BSA for 1 h. The coverslips were then incubated with primary antibodies for 2 h, followed by incubation with the appropriate Alexa Fluor-conjugated secondary antibody for 1 h. Nuclei were stained with DAPI (Beyotime, #C1002) for 15 min at room temperature. The samples were visualized using a confocal microscope (LSM 880; Zeiss, Oberkochen, Germany). Details on statistical analyses are provided in the figure legends. Pearson correlation coefficients for molecular colocalization were calculated using the Coloc 2 plugin in the Fiji software.

### 2.15 Eukaryotic expression and purification of recombinant proteins

The codon-optimized gene encoding the extracellular domain (ECD) of influenza virus HA (HA-ECD) was cloned into the eukaryotic expression vector pCAGGS-HA-His. Similarly, the gene encoding the extracellular domain of CD147 (CD147-ECD) was cloned into the eukaryotic expression vector pCAGGS-CD147-Strep-tag II. HEK293F cells were used for protein expression. When the cell density reached 2.0 × 10^6^ cells/mL, plasmids were pre-mixed with fresh medium and PEI transfection reagent (plasmid: PEI = 1 mg: 2 mL) for 15 mins before transfection. After 60 h, culture supernatants were collected by centrifugation at 4,000 × g for 15 min and filtered through a 0.45 µm mem-brane. The supernatants were diluted in 25 mM HEPES buffer with 150 mM NaCl (pH 7.5). The secreted HA-ECD protein was purified using a Ni-NTA affinity resin. The loaded Ni resin was rinsed with washing buffer A (25 mM HEPES [pH 7.5] and 500 mM NaCl), followed by buffer B (25 mM HEPES [pH 7.5], 150 mM NaCl, and 30 mM imid-azole). Elution was performed using buffer B with 270 mM imidazole. The CD147-Strep-tag II-ECD protein was purified with agarose microspheres and eluted with buffer containing 50 mM biotin. All eluates were further purified via size-exclusion chromatography (SEC; Superose 6 Increase 10/300 GL, GE Healthcare) in 25 mM HEPES (pH 7.5) and 150 mM NaCl. Peak fractions were collected, snap-frozen in liquid nitrogen, and stored at −80°C for subsequent experiments.

### 2.16 Streptavidin pull-down

Purified HA-His and CD147-Streptavidin were mixed at a 1:1 molar ratio and incubated for 1 h at 4°C. Subsequently, the protein mixture was incubated with Strep-II tag protein agarose purification resin at 4°C for 2 h. After washing with HEPES buffer, bound proteins were eluted with 50 mM biotin elution buffer. The samples from each step were analyzed via SDS-PAGE.

### 2.17 Bio-layer interferometry

The interaction affinity between HA and CD147 proteins was analyzed using a BLI system (Octet Red96e, ForteBio). CD147 protein (20μg/mL) was biotinylated using a biotinylation kit (Genemore, 1828M) and immobilized on an Octet Streptavidin Bio-sensor (Sartorius). The biosensors were then immersed in HA protein solutions at various concentrations (0, 6.25, 12.5, 25, 50, 100, 200, and 400 nM) for 10 min. Binding was measured in a binding buffer containing 25 mM HEPES, 150 mM NaCl (pH 7.5), and 0.04% Tween-20. Data were analyzed using Octet Data Analysis HT v12.0 software. Reference sensors and samples were subtracted, and the dissociation constant KD was calculated using a global 1:1 binding model.

### 2.18 Prediction of the interaction structure between CD147 and HA

We used the open-source AlphaFold3 platform on Google Colab to predict the interaction structure of each protein sequence. The sampling parameters were set as follows: an iterative recycling strategy was applied, using the previous output as the input for the next iteration. The iteration was terminated once the preset root mean square deviation tolerance threshold of 0.5 was reached. Specifically, when the difference between two consecutive predictions exceeded 0.5 root mean square deviation, the system stopped cycling and generated the final prediction model. The quality of predictions was evaluated using the following indices: the prediction local distance difference test score, which reflects the confidence level of model prediction; and the interchain predicted template modeling (ipTM) score, which assesses the topological similarity of the protein structures. A confidence threshold of 0.75 was used for ipTM. Predictions were performed using the AlphaFold2 Multimer_v3 model. The highest-scoring final prediction model was visualized using UCSF Chimera v1.17.3 software.

### 2.19 Negative staining

The purified H9-His protein was incubated with huCD147mt-Streptavidin protein at approximately a 1:1 molar ratio for 30 min. The protein mixture was then subjected to SEC using a buffer containing 25 mM HEPES (pH 7.0) and 150 mM NaCl. Protein complexes showing a peak shift were collected. For negative staining, 2.5μl of the protein sample was loaded onto a Formvar-coated grid after pre-hydrophilization and vacuum equilibration. The grid was hydrophilized again and vacuumized. Subsequently, 4μL of 2% uranyl acetate stain was added and incubated for 1 min. Excess liquid was blotted off, leaving a thin layer of stain. Micrographs were collected at 120 kV using a Talos L120C transmission electron microscope (Thermo Fisher Scientific, Waltham, MA, USA). Using Relion software, approximately 900–950 particles were manually selected from 20 micrographs for each structure to generate 2D class averages. No 3D reconstruction was performed in this study.

### 2.20 Infection neutralization experiment

A549 cells were seeded in a 96-well plate and grown overnight. Once the cells reached 90% confluency, they were washed three times in serum-free F-12 medium to prepare for the experiment. The huCD147-Strep protein (final concentrations 0, 300, 600, and 1000μg/mL) and PR8 virus (MOI of 10) were incubated together at 4°C for 1 h. The pre-cooled A549 cells were then infected with the protein–virus mixture at 4°C for 1 h to allow viral adsorption, followed by washing with cold serum-free F-12 medium. Finally, the medium was replaced with maintenance medium containing the corresponding concentration of huCD147-Strep protein (0, 300, 600, and 1000μg/mL). After 36hpi, the virus-containing supernatants were collected to determine viral titers.

### 2.21 Antibody blocking experiment

The antibody blocking experiment was performed using an anti-CD147 rabbit monoclonal antibody targeting the ECD of CD147. A rat IgG2a monoclonal antibody was used as the isotype control. A549 cells were seeded in a 96-well plate and grown over-night. After reaching 90% confluency, cells were washed three times with serum-free F-12 medium to prepare for the experiment. The anti-CD147 antibody was adjusted to concentrations of 10, 30, and 50 μg/mL, while the IgG2a control antibody was used at 50 μg/mL. Cells were incubated with the antibodies at 4°C for 1 h. After discarding the antibodies, PR8 virus (MOI of 10) was added and allowed to adsorb at 4°C for 1 h. Un-bound virus was washed off with cold serum-free F-12 medium. Cells were then cultured in maintenance medium containing the corresponding anti-CD147 concentration at 37°C for 36 h. The supernatants were collected for virus titration.

### 2.22 Statistical analysis

Statistical analyses were performed using GraphPad Prism v6.0 software (GraphPad Prism, San Diego, CA). Unpaired Student’s t-tests or one-way analysis of variance (ANOVA) were applied, and two-way ANOVA was used when appropriate. P-values < 0.05 were considered statistically significant, using the following notation: *, P < 0.05; **, P < 0.01; ***, P < 0.001; and ****, P < 0.0001.

## 3 Results

### 3.1 Screening host membrane proteins interacting with HA and Regulating proliferation of AIV

**Figure 1:**
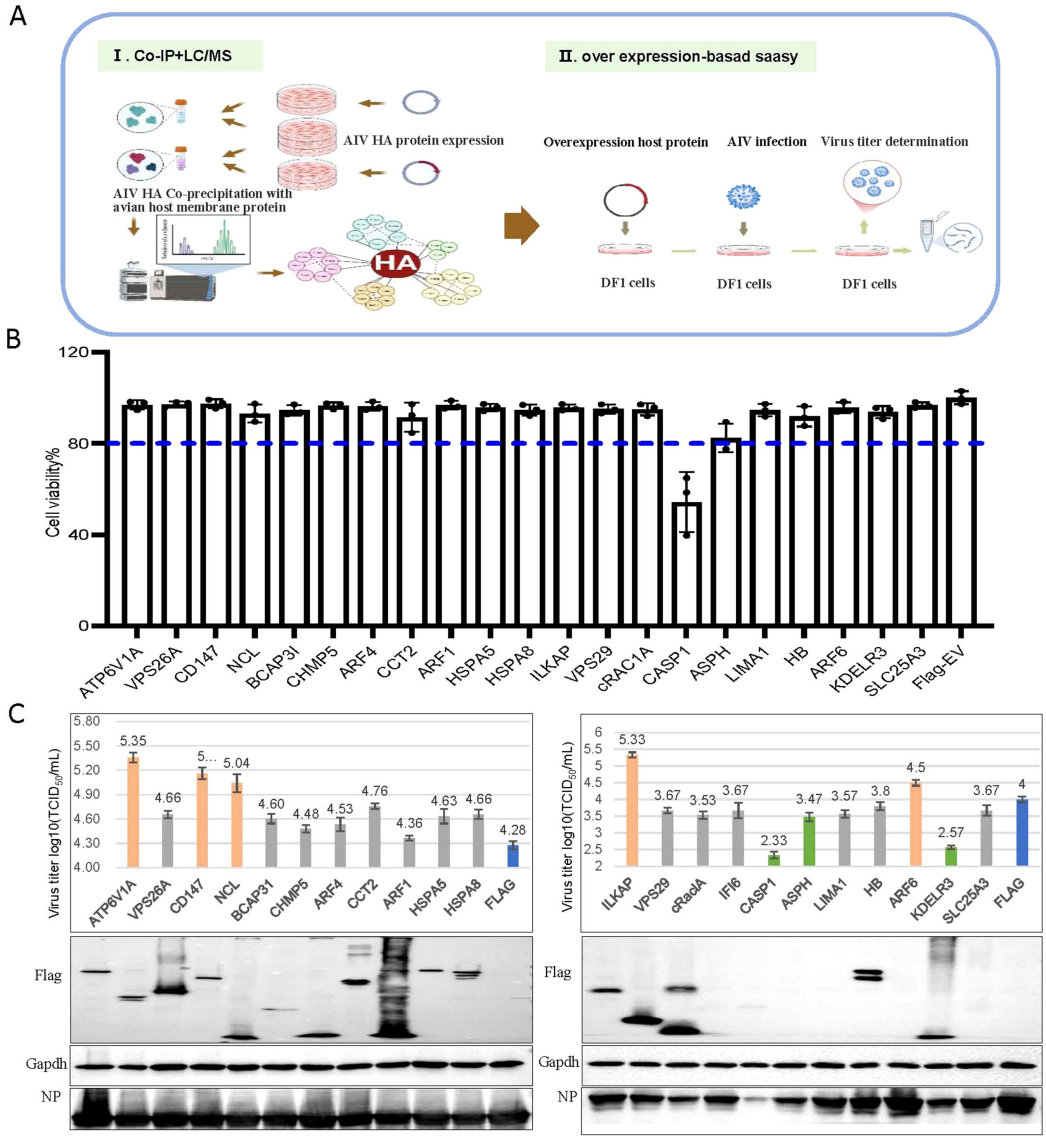
CD147 was identified to positively regulate the proliferation of the H5N6 influenza virus. **(A)** Schematic diagram of the identification of host proteins that coprecipitated with influenza A viral protein HA. (I) Mass spectrometry was used to identify host proteins that coimmunoprecipitated with Ha-tagged influenza viral protein HA. (II) To identify host factors that affected viral replication, cells were transfected with plasmids to overexpress each of the candidate host genes and then infected with influenza virus. Cell viability, Virus titers and protein were then determined. **(B)** Viability of DF1 cells transfected with the indicated plasmids. **(C)** DF1 cells were transfected with the indicated plasmids, infected with H5N6 virus at an MOI of 0.001, the supernatant was collected for viral titer measurement, and the cells were harvested and analyzed via WB.

We first investigated influenza HA–host protein interactions that are implicated in H5N6 virus replication in chicken embryo fibroblast DF-1 cells (schematic diagram in **Figure 1A**). Using site-directed mutagenesis, we replaced the basic amino acid sequence (GERRRKKR) at the HA cleavage site with neutral amino acids (GETR), thereby maintaining the HA protein in a stable prefusion trimeric conformation (Spronken et al., 2024). We expressed H5N6 HA protein with an HA tag in DF-1 cells, enriched the HA protein via anti-Ha antibody-coupled magnetic beads, and confirmed protein integrity by western blot (**Supplementary Figure S1A**). The enriched HA protein was then subjected to co-IP with DF1 cell membrane proteins, and mass spectrometry identified 25 host proteins that co-precipitated with HA (**Supplementary Figure S1B**).

We successfully cloned 20 candidate host genes and two control genes into the p3×Flag-CMV-14 vector. Notably, we selected ATP6V1A (Banerjee et al., 2013; Li et al., 2020; Haas et al., 2023) and CAPS1 (Bauer et al., 2012; Coates et al., 2014; Ren et al., 2017) as functional controls due to their reported significant effects on the proliferation of H1N1, H3N2, and H5N1 strains. After overexpressing these genes in DF1 cells, we assessed cytotoxicity using the CCK-8 assay 24 h post-transfection and infected cells with the H5N6 virus at an MOI of 0.001. At 24 hpi, we collected supernatants for virus titer determination and cell lysates for western blotting. Overexpression of the candidate genes did not affect cell viability (all above 80%) or subsequent infection experiments. As expected, overexpressing CAPS1, which is involved in inflammatory responses and apoptosis, significantly reduced cell viability, validating our approach (**Figure 1B**). Ultimately, we identified four genes (CD147, NCL, ILKAP, and ARF6) that positively regulated virus proliferation (virus titer increased by over 0.5) and two genes (KDELR3 and ASPH) that negatively regulated it (virus titer decreased by over 0.5) (**Figure 1C**). Given that CD147 is involved in the infection process of several pathogens, we further explored the regulatory mechanism of CD147 on the proliferation of influenza viruses.

### 3.2 Host membrane protein CD147 promotes the proliferation of influenza virus

The role of CD147 in influenza virus infection has not been studied. Given that we identified it as a positive regulator of influenza virus proliferation, we investigated the specific role of CD147 in AIV infection.

**Figure 2:**
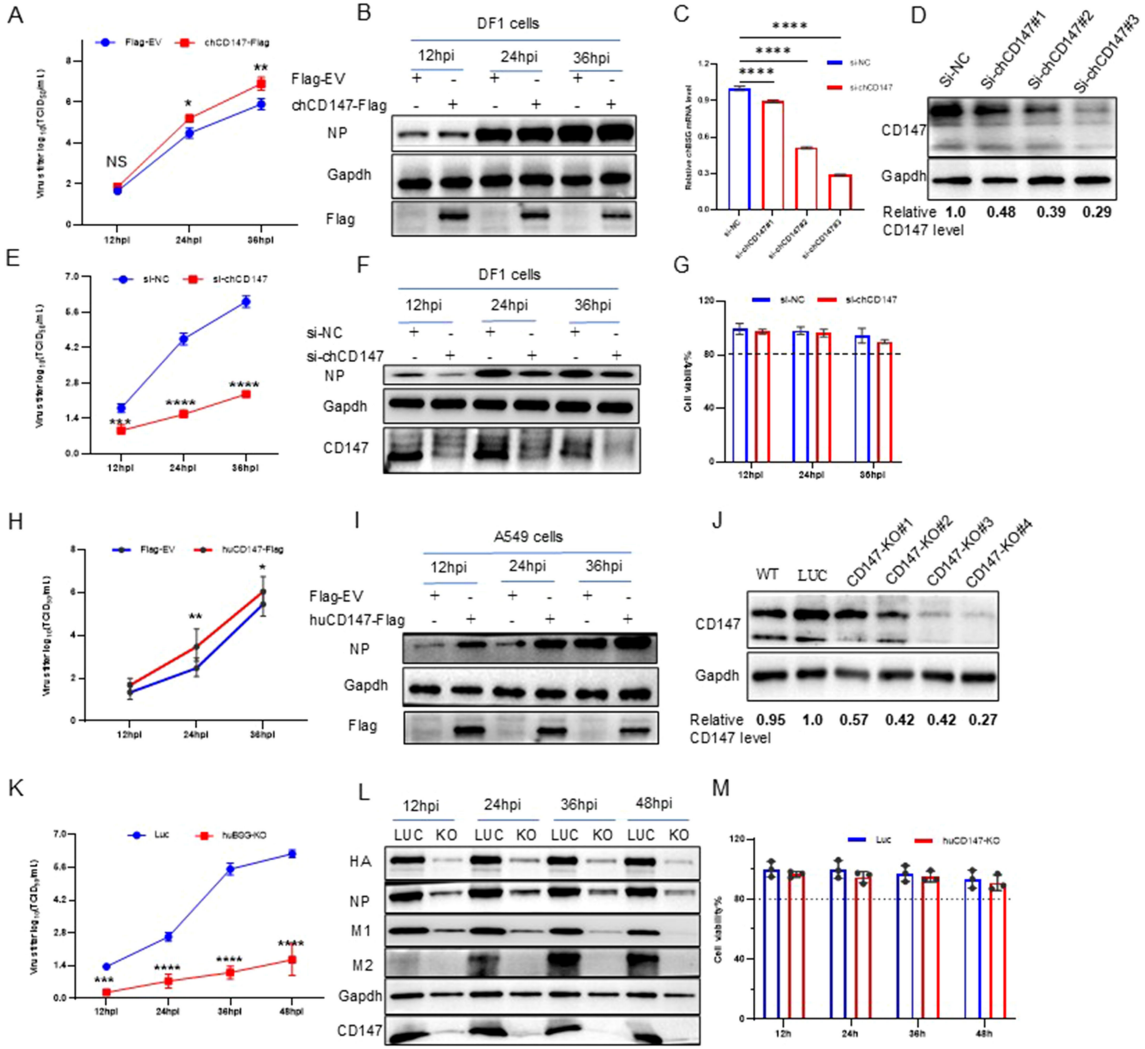
Host membrane protein CD147 promotes the proliferation of influenza virus. **(A, B)** DF1 cells were transfected with chCD147-Flag or Flag-Empty Vector (EV), and infected with H5N6 virus 24 h after transfection. Supernatant was collected at different time points for TCID_50_ measurement **(A)**, and the cells were harvested and analyzed via WB **(B)**. **(C, D)** DF1 cells were transfected with siRNA-chCD147 or siRNA-negative control (NC), and cells were harvested and analyzed via RT-qPCR **(C)** and Western blotting (WB) **(D)**. DF1 cells were infected with H5N6 virus 24 h after transfection with siRNAs. Supernatant was collected at different time points for TCID_50_ measurement **(E)**, and the cells were harvested and analyzed via WB **(F)**. Cell viability was determined at the indicated time points **(G)**. **(H, I)** A549 cells were transfected with huCD147-Flag or Flag-EV, and infected with H5N6 virus 24 h after transfection. Supernatant was collected at different time points for TCID_50_ measurement **(H)**, and the cells were harvested and analyzed via WB **(I)**. **(J)** The effect of knockdown in A549 cells was analyzed via WB. **(K–M)** CD147 knockdown A549 cells were infected with H1N1 (PR8) virus, and the supernatant was collected at different time points for TCID_50_ measurement **(K)**. Cells were harvested and analyzed via WB **(L)**. Cell viability was determined at the indicated time points **(M)**. Error bars, mean ± SD of three experiments. All comparisons were tested by two-tailed Student’s t test; *, P, 0.05; **, P, 0.01; ***, P, 0.001; ****, P, 0.001.

To verify the role of CD147 in promoting the proliferation of AIV, we examined virus replication by overexpressing (chCD147-Flag) or silencing CD147 (si-chCD147) in avian DF-1 cells. Overexpression significantly promoted the proliferation of AIV (**Figures 2A, B**). Three siRNAs were designed and synthesized to target chCD147 in DF1 cells, with knockdown efficiency confirmed via RT-qPCR and western blotting. Of the three siRNAs, si-chCD147#3 reduced chCD147 expression by more than 70% (**Figures 2C, D**). This reduction of chCD147 significantly lowered the viral titers at 12 hpi, with the effect more obvious as the virus replication cycle progressed (**Figures 2E, F**). In addition, to rule out the possibility that silencing chCD147 caused cytotoxicity in DF-1, we assessed cell viability, which showed that silencing chCD147 had no significant effect on cell viability (**Figure 2G**).

It is well established that influenza viruses are zoonotic pathogens capable of cross-species transmission (Mostafa et al., 2018). While CD147 exhibits significant sequence homology across species, the homology between human and chicken CD147 is relatively low (~55%), with only 34.7% amino acid similarity in their ECDs (**Supplementary Figure S2A**). Notably, human CD147 contains three N-glycosylation sites in its extracellular region, whereas chicken CD147 harbors five distinct N-glycosylation modifications (**Supplementary Figure S2B**). To explore the influence of human CD147 on influenza virus proliferation, we transfected human CD147 (huCD147-Flag) into A549 cells for 24 h, then infected the cells with H1N1 (PR8) virus at an MOI of 0.01. Samples were collected at different time points to determine viral titers. Overexpression of huCD147 significantly promoted virus proliferation (**Figures 2H, I**). We then constructed a CD147-KO A549 cell line using the CRISPR/Cas9 system and successfully obtained four subclonal cell lines. Western blotting revealed that the knockdown efficiency of CD147-KO#4 was over 70% (**Figure 2J**). Furthermore, huCD147 KO cell lines and control cells were infected with the H1N1 (PR8) strain at an MOI of 0.01. Knocking down huCD147 significantly reduced influenza virus proliferation (**Figures 2K, L**). In addition, continuous monitoring of cell viability showed that KO of huCD147 had no significant effect on the cell viability (**Figure 2M**). In summary, the surface membrane protein CD147, from both human and avian sources, can promote the infection and proliferation of influenza virus.

### 3.3 Host membrane protein CD147 interacts with influenza virus HA protein

**Figure 3:**
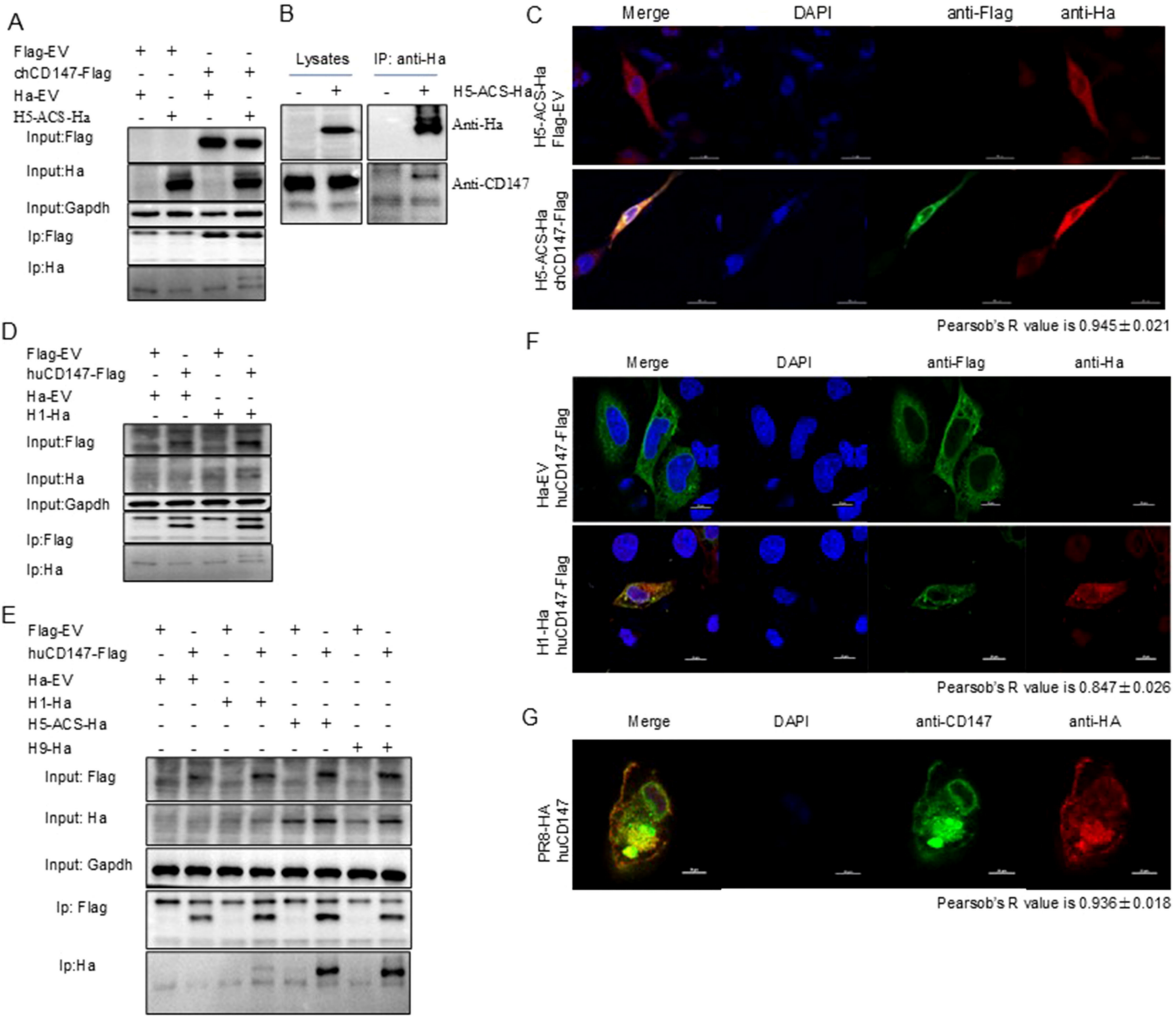
CD147 interacted with HA. **(A)** HEK293T cells were transfected with plasmids encoding chCD147-Flag and H5-ACS-Ha. The lysates were subjected to anti-Flag IP and analyzed via Western blotting (WB). **(B)** DF1 cells were transfected with plasmids encoding H5-ACS-Ha. The lysates were subjected to anti Ha IP and analyzed via WB. **(C)** DF1 cells were transfected with plasmids encoding H5-ACS-Ha (H5N6 virus) and chCD147-Flag (avian source) and analyzed for colocalization of H5-ACS-Ha and chCD147-Flag. Scale bar, 20 mm. **(D)** HEK293T cells were transfected with plasmids encoding huCD147-Flag (human source) and H1-Ha (H1N1 virus), the lysates were subjected to anti-Flag IP and analyzed via WB. **(E)** HEK293T cells were transfected with plasmids encoding huCD147-Flag (human source) and H1-Ha (H1N1 virus), H5-ACS-Ha (H5N6 virus), H9-Ha (H9N2 virus) respectively, the lysates were subjected to anti-Flag IP and analyzed via WB. **(F)** Hela cells were transfected with H1-Ha and huCD147-Flag plasmid, and analyzed for colocalization of H1-Ha and huCD147-Flag. Scale bar, 20 mm. **(G)** A549 cells were infected with H1N1 (PR8), and analyzed for colocalization of CD147 and HA of H1N1 virus. Scale bar, 20 mm.

CD147 was identified in the AP-MS results of HA, suggesting a potential interaction between the two proteins. We used Co-IP and indirect immunofluorescence experiments to explore their interaction and subcellular localization in cells. As expected, chCD147-Flag co-precipitated with H5-ACS-Ha (**Figure 3A**), and endogenous CD147 co-precipitated with H5-ACS-Ha in DF-1 cells (**Figure 3B**). In addition, confocal microscopy showed that chCD147-Flag co-localized with H5-ACS-Ha in DF-1 cells, and both were distributed in a reticular pattern within the cytoplasm (**Figure 3C**). Pearson’s r value = 0.945 ± 0.021, indicating a high degree of colocalization (Costes et al., 2004). Further supporting the interaction, chCD147-Flag and H5-ACS-Ha were confirmed to co-precipitate, indicating that the two proteins interact (**Figure 3D**). Additional studies on human CD147 further validated these findings. Co-IP experiments demonstrated that huCD147-Flag co-precipitated H1-Ha, H5-ACS-Ha, and H9-Ha in HEK293T cells (**Figures 3D, E**). Confocal microscopy showed that huCD147-Flag and H1-Ha were distributed in both the cytoplasm and the cell membrane in HeLa cells, displaying a reticular cytoplasmic pattern, and were co-localized (r = 0.847 ± 0.026) (**Figure 3F**). During the virus life cycle in A549 cells, CD147 and virus HA (PR8) were also co-localized at the cell membrane and in the cytoplasm (r = 0.936 ± 0.018) (**Figure 3G**). In summary, these results provide evidence supporting the interaction between the host cell membrane protein CD147 and influenza virus HA protein.

### 3.4 Host membrane protein CD147 promotes the adsorption of influenza virus

**Figure 4:**
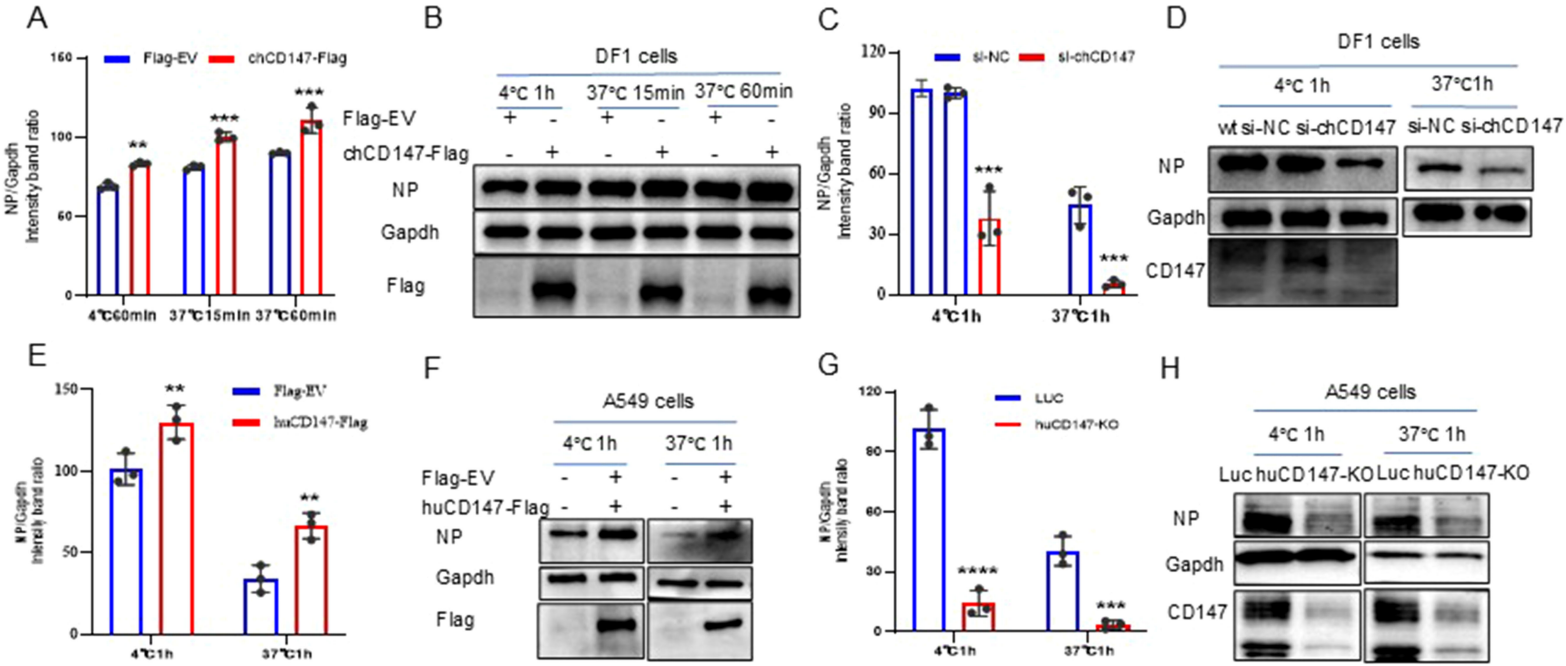
CD147 promotes the absorption/endocytosis of influenza virus. **(A, B)** DF1 cells were transfected with chCD147-Flag and infected with H9N2 virus (MOI 50) 24 h after transfection at 4°C, after 1h of adsorption, they were transferred to 37°C for 15min and 60min. Samples of adsorbed and internalized cells were collected, vRNA was quantitatively analyzed by RT-qPCR **(A)** and Western blotting (WB) **(B)**. **(C, D)** DF1 cells were transfected with siRNA-chCD147 or siRNA-NC, and infected with H9N2 virus (MOI 50), washed with cold serum-free DMEM after 1 hour, the cells were cultured at 37°C for 1h, and then washed with acid PBS (pH 1.3), the vRNA was quantitatively analyzed by RT-qPCR **(C)** and Western blotting (WB) **(D)**. **(E–H)** A549 cells were transfected with huCD147-Flag or Flag-EV and CD147 knockdown A549 cells, infected with H1N1 (PR8) virus at 4°C, washed with cold serum-free Ham’s F-12 after 1 hour, the cells were cultured at 37°C for 1h, and then washed with acid PBS (pH 1.3), the vRNA was quantitatively analyzed by RT-qPCR **(E, G)** and Western blotting (WB) **(F, H)**. Error bars, mean ± SD of three experiments. All comparisons were tested by two-tailed Student’s t test; **, P, 0.01; ***, P, 0.001; ****, P, 0.001.

To explore the role of CD147 in the process of influenza virus infection, we over-expressed (chCD147-Flag) and knocked down (si-chCD147) CD147 expression in DF-1 cells and analyzed the levels of vRNA adsorbed on the cell membrane surface and internalized into cells via RT-qPCR and western blotting. Overexpression of chCD147 significantly enhanced the adsorption of the H9N2 virus on the surface of DF-1 cells and markedly increased the number of virus particles entering the cells (**Figures 4A, B**). In contrast, knocking down CD147 significantly inhibited both the adsorption and entry of the virus into DF-1 cells (**Figures 4C, D**). Similarly, CD147 expression in A549 cells sig-nificantly promoted the adsorption and endocytosis of H1N1 (PR8) virus particles (**Figures 4E, F**), whereas CD147 knockdown significantly reduced the adsorption and endocytosis of virus particles (**Figures 4G, H**). In summary, the host surface membrane protein CD147 plays an important role during the adsorption stage of influenza virus infection.

### 3.5 Role of CD147 glycosylation in influenza virus infection and adsorption

**Figure 5:**
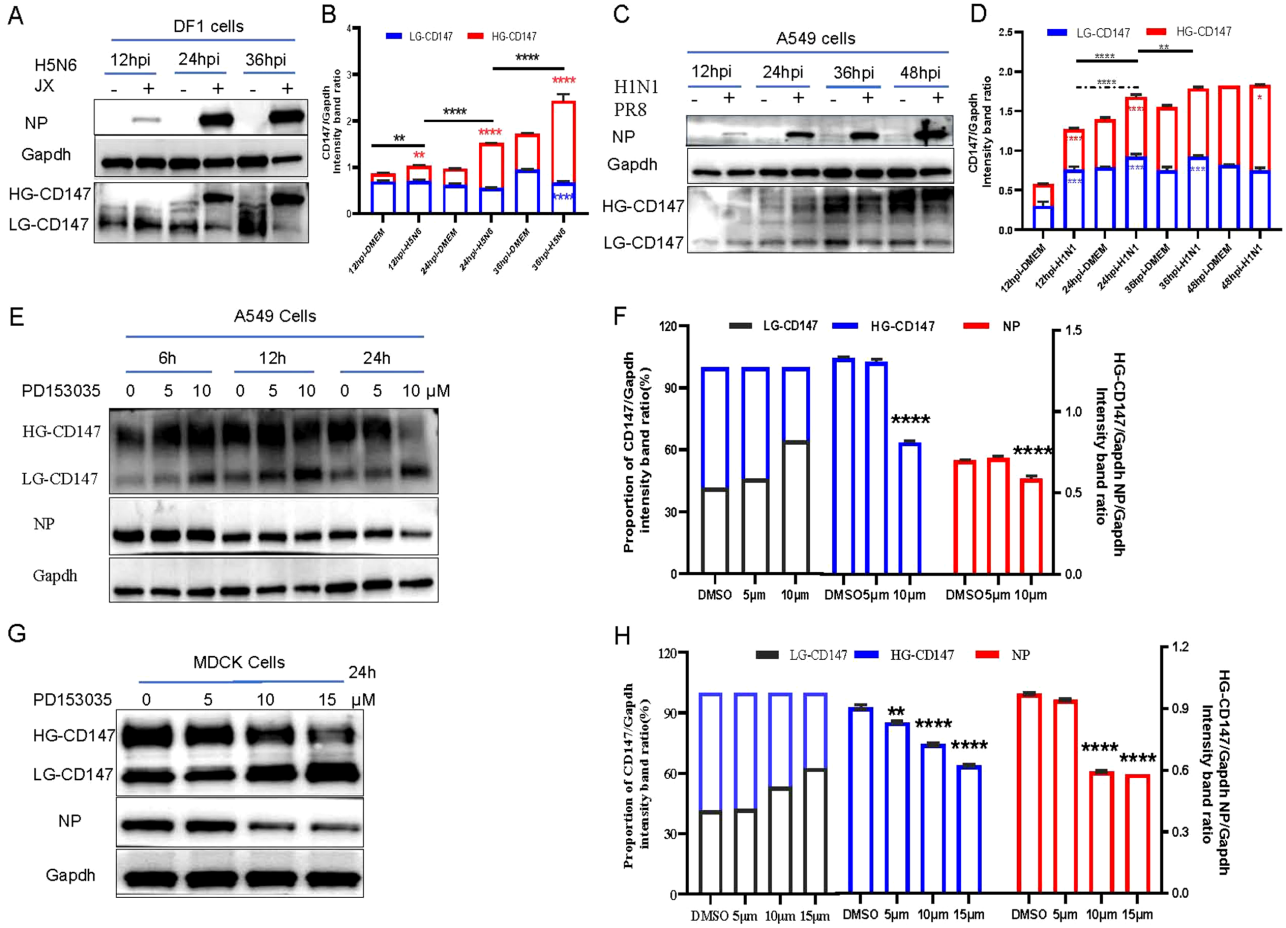
Virus infection, adsorption and glycosylation modified form of CD147. **(A)** DF1 cells were infected with H5N6 (JX) virus (MOI 0.001), and the cells were harvested and analyzed the change of protein level via WB. **(C)** A549 cells were infected with H1N1 (PR8) virus (MOI 0.01), and the cells were harvested and analyzed the change of protein level via WB. **(E)** A549 cells were exposed to PD1530356h, 6h 12h and 24h with different concentrations, and infected with PR8(MOI 10) at 4°C for 1h, the cells were harvested and analyzed the change of protein level via WB. **(G)** MDCK cells were exposed to PD1530356h 24 hours, and infected with H9N2 (MOI 10) at 4°C for 1h, the cells were harvested and analyzed the change of protein level via WB. **(B, D, F, H)** Gapdh was used as the control, Image J(NIH) was used to quantitatively analyze the gray level of protein bands, and CD147/Gapdh indicated the relative level of CD147. All comparisons were tested by two-tailed Student’s t test, the solid line represents the difference of HG-CD147 in different infection periods, and the dotted line represents the difference of LG-CD147 in different infection periods, blue * represents the difference of LG-CD147 in the same infection period, red * represents the difference of HG-CD147 in the same infection period, and black * represents the difference of total protein of CD147. *, P, 0.05; **, P, 0.01; ***, P, 0.001; ****, P, 0.001.

To explore the function of CD147 in the life cycle of the influenza virus, we further studied the dynamic changes of CD147 expression during virus infection. We infected DF-1 and A549 cells with H5N6 (MOI of 0.001) and H1N1 (PR8 MOI of 0.01), respectively. Samples were collected at different time points to detect CD147 protein levels via western blotting. The overall level of CD147 protein in cells increased significantly with the infection and proliferation of both H5N6 (**Figures 5A, B**) and H1N1 (**Figures 5C, D**) viruses.

CD147 exists in two glycosylated forms: hyperglycosylated form (HG-CD147) and low-glycosylated CD147 (LG-CD147). To further explore the effects of HG-CD147 and LG-CD147 on influenza virus adsorption, we explored the mechanism by which CD147 glycosylation regulates virus adhesion. Glycosylation of CD147 is regulated by the inhibitor PD153035 (Grass and Toole, 2015). Western blotting results showed that while the total amount of CD147 protein in cells remained largely unchanged with prolonged inhibitor treatment, LG-CD147 levels gradually increased and HG-CD147 levels significantly decreased due to glycosylation inhibition (**Figures 5E-H**). Treatment of A549 and MDCK cells with different concentrations of PD153035 showed that 10 µM PD153035 significantly reduced HG-CD147 protein levels in both A549 (**Figures 5E, F**) and MDCK (**Figures 5G, H**) cells. Meanwhile, the amount of NP protein adsorbed from the virus was also significantly reduced, indicating that the number of virus particles adsorbed onto the cell surface decreased markedly. In summary, glycosylation of the host cell CD147 protein facilitates virus adsorption and proliferation.

### 3.6 The interaction between CD147 and HA is independent of CD147 glycosylation

**Figure 6:**
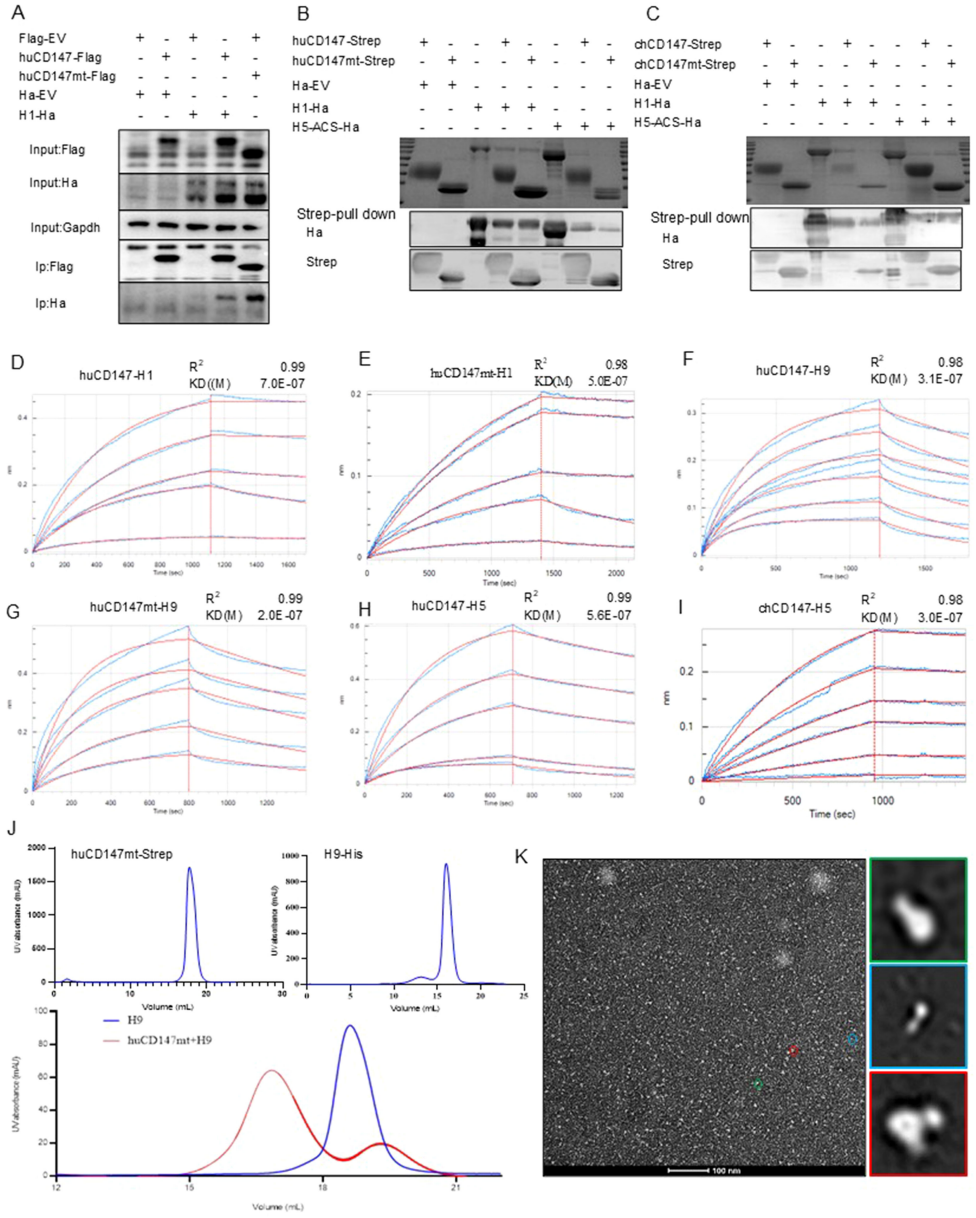
The direct interaction between CD147 and influenza HA proteins is independent of the glycosylation modification of CD147. **(A)** HEK293T cells were co-transfected with huCD147-Flag and H1-Ha (PR8) plasmids, respectively, the lysates were subjected to anti-Flag IP and analyzed via WB. **(B, C)** Eukaryotic expression plasmids huCD147-strep, huCD147mt-strep, chCD147-strep, chCD147mt-strep, H1-His (H1N1) and H5-ACS-His were transfected into HEK293F suspension cells for expression, and the soluble proteins were purified by the coupled Strep tag and Ha tag, then the pull-down experiment was carried out. **(D–I)** The BLI data is analyzed by the software of Octet data analysis HT12.0, the reference sample and reference sensor are subtracted, and the KD value is analyzed by using the 1:1 global fitting model. **(D)** the binding dynamic curve of huCD147 and H1 (H1N1), **(E)** Dynamic curve of the combination of huCD147mt and H1 (H1N1), **(F)** Dynamic curve of the combination of huCD147 and H9 (H9N2), **(G)** Dynamic curve of the combination of huCD147mt and H9 (H9N2), **(H)** The binding dynamic curve of huCD147 and H5 (H5N6), **(I)** The binding dynamic curve of chCD147 and H5(H5N6). **(J)** The OpNS EM images of huCD147mt-Strep protein, H9-his (H9N2) protein and their co-incubation products passing through the molecular sieve respectively. **(K)** shows an overview view of the micrograph. The 2D class average of more than 50,000 particles selected from 50 micrographs, which are calculated by HA (H9N2) particles (green), CD147 (huCD147) particles (green) and HA-CD147 complex (red) respectively. Scale: 100nm.

Host factor CD147 is a highly N-glycosylated protein (Yoshida et al., 2000), with SA residues at the termini of its sugar chains (Bai et al., 2014). To further investigate the specific mechanism by which CD147 influences influenza virus adsorption, we mutated the asparagine at the N-glycosylation site of human CD147 to glutamine, creating a glycosylation-deficient mutant expression plasmid, huCD147mt-Flag. The H1-HA (PR8-derived) plasmid was co-transfected with huCD147-Flag or huCD147mt-Flag into HEK293T cells. Both huCD147-Flag and huCD147mt-Flag co-precipitated with H1-HA, indicating a physical interaction between them (**Figure 6A**).

To further verify this result, we constructed eukaryotic expression plasmids: huCD147-Strep, huCD147mt-Strep, chCD147-Strep, chCD147mt-Strep, H1-His, H5-ACS-His, and H9-His. Soluble proteins were expressed and purified in HEK293F suspension cells. The effect of CD147 glycosylation on its interaction with virus HA proteins was assessed using Streptavidin pull-down and BLI experiments. The Streptavidin pull-down assay showed that both huCD147-Strep and huCD147mt-Strep proteins could precipitate H1-His and H5-ACS-His (**Figure 6B**), and similarly, chCD147-Strep and chCD147mt-Strep could precipitate H1-His and H5-ACS-His (**Figure 6C**). These results demonstrate that the host CD147 interacts directly with influenza virus HA proteins and that glycosylation-deficient CD147 mutants can also interact directly with HA proteins. BLI experiments revealed that compared to huCD147 (**Figure 6D**), the glycosylation-deficient huCD147mt (**Figure 6E**) exhibited stronger binding to H1 HA protein, with KD of 7.0 × 10–^7^ and 5.0 × 10–^7^ for huCD147 and huCD147mt, respectively. Similarly, the binding of huCD147mt to H9 HA protein (**Figure 6G**) was slightly stronger than that of huCD147 (**Figure 6F**), with KD values of 2.0 × 10–^7^ and 3.1 × 10–^7^ for huCD147mt and huCD147, respectively. These findings suggest that CD147 glycosylation does not promote binding to influenza virus HA proteins; rather, the LG-CD147 exhibits enhanced binding.

Next, to explore the binding affinity of CD147 with different influenza virus sub-types, we compared the KD values of huCD147 with H1 (H1N1), H5 (H5N6), and H9 (H9N2). The data showed that huCD147 had a stronger binding to avian H5 (**Figure 6H**) and H9 HA proteins, with KD values of 5.6 × 10–^7^ and 3.1 × 10^-7^, respectively, compared to H1 HA. The binding affinity of H5 HA and CD147 from a different host (chicken) was ananlyzed. The KD value of H5 HA and chCD147 was 3.0 × 10^-7^ (**Figure 6I**), and this binding was stronger than that of H5 and huCD147.

To visually characterize the interaction between CD147 and HA protein and assess the morphological features of protein complexes, purified huCD147mt-Strep and H9-His proteins were incubated at an approximate 1:1 molar ratio for 30 min, followed by SEC. Compared to individual proteins, the elution peak of the protein complex shifted toward an earlier retention volume (**Figure 6J**), indicating that the two secreted proteins assembled into larger macromolecular complexes. Negative-stain electron microscopy of the complex revealed distinct structural features: cone-shaped and trimerically symmetric H9-ECD particles (green), rod-shaped CD147 particles (green), and additional rod-shaped density (red) localized at the head region of H9-ECD particles, likely representing the CD147-HA interaction complex (**Figure 6K**). In summary, the host cell membrane protein CD147 interacts directly with influenza virus HA protein independently of its glycosylation.

### 3.7 Two extracellular immunoglobulin-like domains of CD147 participate in interaction with the RBD of HA

**Figure 7:**
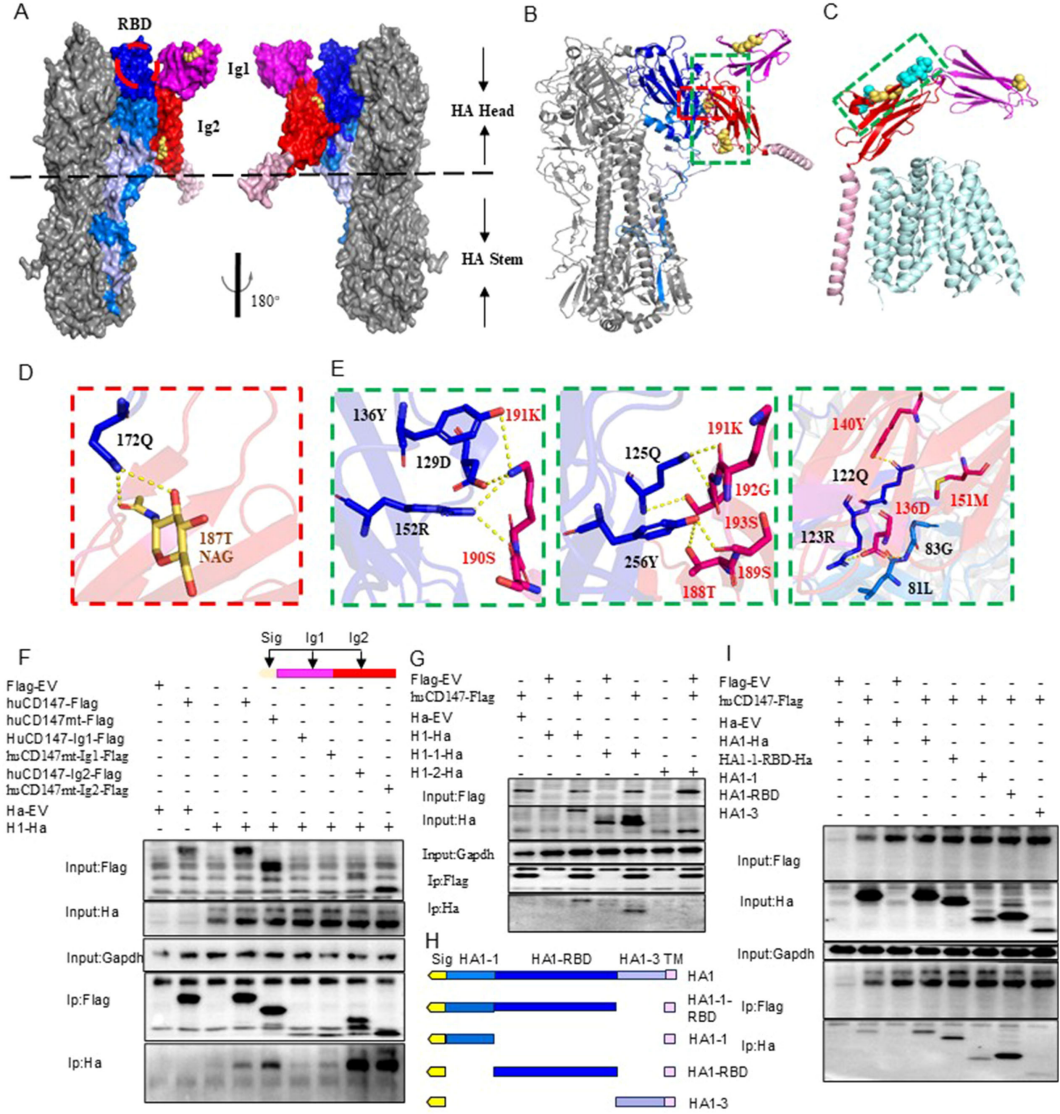
Structural analysis of interaction between CD147 and HA protein. **(A, B)** are the surface display of the binding domain structure of CD147 and HA protein extracellular domain, respectively, showing two top views. CD147 binds to a region very close to the receptor binding domain (RBD) of HA, which is surrounded by a red dotted line. HA is shown in gray. Dark blue, blue and light blue respectively represent HA1-1, HA1-RBD and HA1-3. CD147-Ig1, CD147-Ig2 and CD147-TM are represented by magenta, red and pink respectively. N-glycosylation sites are shown in yellow. **(B, D, E)** The docking interface between HA and CD147 is determined, and the residues are displayed as rods. The overall structural model of the HA-CD 147 binding region **(B)**. The area surrounded by red and green dotted lines is shown in detail in **(D, E)**. **(C)** The key residues of the interface between HA and CD147 are displayed in the structure of the complex of human MCT1 and CD147 (PDB 6LZ0) through spheres, and the interface between HA and CD147 is analyzed in detail. The polarity of the interaction is indicated by the yellow dotted line. **(F)** HEK293T cells were co-transfected H1-Ha (PR8) plasmids with huCD147-Flag, huCD147-Ig1-Flag and huCD147-Ig2, respectively, the lysates were subjected to anti-Flag IP and analyzed via WB. **(G)** HEK293T cells were co-transfected huCD147-Flag plasmids with H1-Ha (PR8) H1-Ha, H1-1-Ha and H1-2-Ha, respectively, the lysates were subjected to anti-Flag IP and analyzed via WB. **(H)** Schematic diagram of truncated HA1(HIN1) sequence. **(I)** HEK293T cells were co-transfected huCD147-Flag plasmids with HA1-1-ha, HA1-1-RBD-ha, HA1-RBD-ha and HA1-3-ha, respectively, the lysates were subjected to anti-Flag IP and analyzed via WB.

To further investigate the molecular details of the interaction, we predicted the structures of the trimeric HA protein (H1N1) and human CD147 (huCD147) protein using AlphaFold3. Compared with previously published structures, the predicted models showed no significant deviations (**Supplementary Figures S3A, B**). To enhance prediction accuracy, N-acetylglucosamine molecules were added at three N-glycosylation sites of CD147 (N44, N152, and N186) to model the potential complex structure formed between CD147 and HA protein. The results indicated that two immunoglobulin-like domains in the extracellular region of CD147 interact with regions of the RBD on the head and face of HA (**Figures 7A, B**). At the interaction interface, nine residues of CD147 were involved: the side chains of D136, Y140, T188, S189, S190, K191, and S193, and the main chains of M151 and G192. On the HA side, six residues (Q125, R126, Q128, D132, Y139, and R155) located near the 130-loop and 150-loop of the RBD binding pocket in the head and face of HA and three residues (G86, 84L, and Y259) in the highly conserved VE region at the base participated in the interaction. By comparing the interface between HA and CD147 with the published complex structure of human MCT1 and CD147 (PDB 6LZ0) (Wang N. et al., 2021), we found that the key amino acid residues involved in HA–CD147 binding are located in solvent-accessible external regions of CD147 (**Figure 7C**).

To verify the interaction domains between CD147 and HA, we truncated the extracellular regions of CD147 and HA based on their functional domains and performed Co-IP combined with structural predictions. Co-IP results showed that full-length huCD147-Flag, as well as truncated huCD147-Ig1-Flag and huCD147-Ig2-Flag, co-precipitated with H1-Ha, with the Ig2 domain showing the strongest interaction (**Figure 7F**). Additionally, compared with wild-type protein, CD147 lacking glycosylation (huCD147mt-Flag, huCD147mt-Ig1-Flag, and huCD147mt-Ig2-Flag) co-precipitated more HA, consistent with the Streptavidin pull-down (**Figure 6B**) and BLI (**Figures 6D, E**) results. HA (H1N1) was then truncated into HA1 (H1-1-Ha) and HA2 (H1-2-Ha). Co-IP experiments showed that huCD147-Flag co-precipitated with both full-length H1-Ha and HA1, but not with HA2 (**Figure 7G**), suggesting the interaction occurs in the HA1 region. Furthermore, HA1 was truncated into four segments based on its three domains (**Figure 7H**). huCD147-Flag co-precipitated with all four truncated proteins (HA1-1-Ha, HA1-1-RBD-Ha, HA1-RBD-Ha, and HA1-3-Ha), with the strongest binding seen in the HA1-RBD region (**Figure 7I**). In summary, these results confirm that the two immunoglobulin-like domains in the extracellular region of CD147 directly interact with the RBD on the head of HA, consistent with structural modeling predictions.

### 3.8 Disrupting the CD147–HA interaction inhibits AIV adsorption and replication

**Figure 8:**
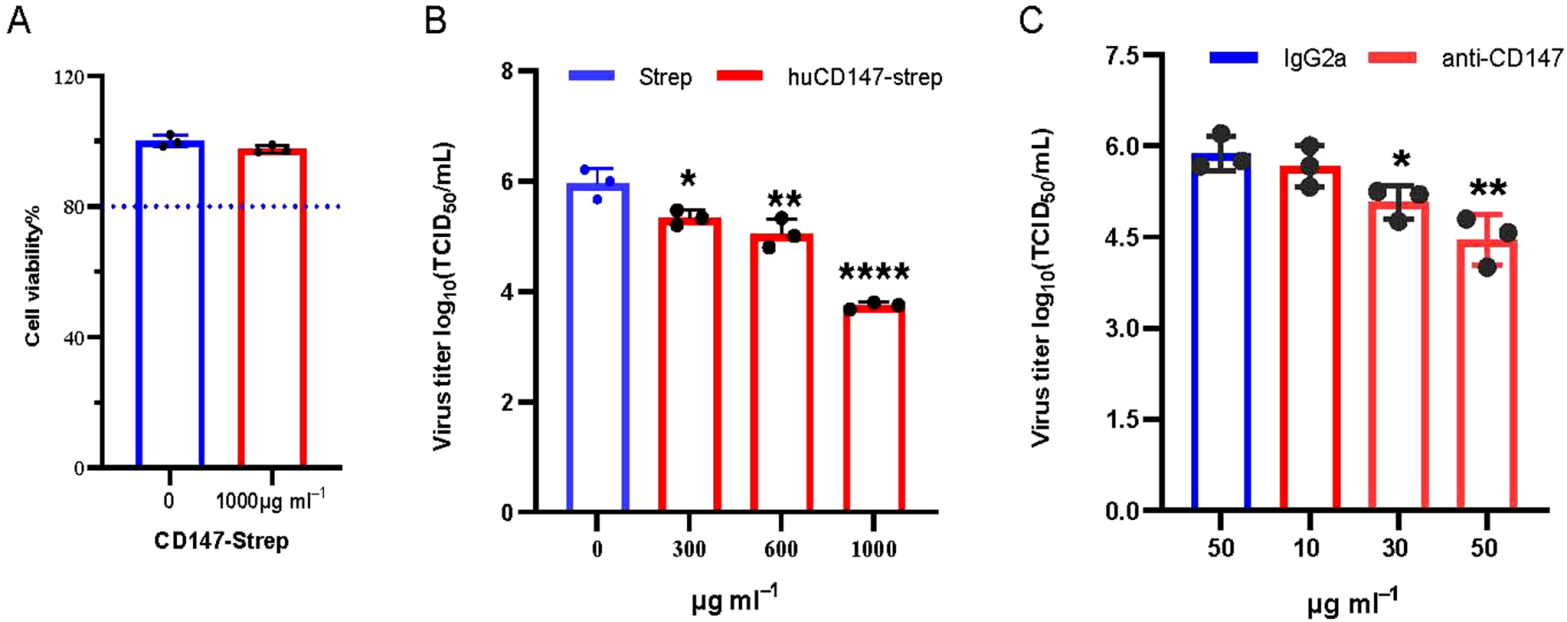
Blocking the interaction between cell surface CD147 and influenza virus HA inhibits the proliferation of influenza virus. **(A)** 1000μg/mL CD147-Strep protein did not affect the viability of A549 cells. **(B)** Soluble protein CD147-Strep inhibited the replication of H1N1 in A549 cells. The CD147-Strep protein was incubated with the virus at the concentration gradient of 0,300,600 and 1000μg/mL for 1 hour at 4°C, and the A549 cells were infected with the protein-virus mixture. After the virus proliferated for 36 hours, the supernatant virus solution was collected to determine the TCID50. **(C)** Monoclonal antibody against CD147 extracellular domain significantly reduced the adsorption of H1N1 on A549 cells. Anti-CD147 and IgG2a with concentrations of 10, 30, 50μg/mL and 50μg/mL were used to incubate A549 cells at 4°C for 1 hour, discarded the antibody and infected with PR8 (MOI 10) at 4°C, washed with cold serum-free Ham’s F-12 after 1 hour. The virus proliferated for 36hpi, and the virus supernatant was collected to determine TCID50. *, P, 0.05; **, P, 0.01; ****, P, 0.001.

The preceding experiments demonstrated that influenza virus HA directly interacts with host membrane protein CD147. We hypothesize that this interaction facilitates the adsorption of influenza virus particles onto the host cell surface. To verify the functional significance of the CD147–HA interaction during virus adsorption, we designed two complementary experimental approaches. First, we used a soluble CD147 extracellular domain protein to competitively bind viral HA protein, thereby preventing the virus particles from attaching to host cells. HuCD147-Strep protein showed no cytotoxicity at the highest tested concentration (**Figure 8A**). The huCD147-Strep protein (concentrations 0, 300, 600, and 1000μg/mL) was incubated with PR8 (H1N1) virus at an MOI of 10 at 4°C for 1 h, followed by viral adsorption under cold conditions. The results showed that huCD147-Strep effectively inhibited viral infection of A549 cells, and the effect was enhanced with increasing protein concentration (**Figure 8B**). Second, we performed an antibody block assay using anti-CD147, a monoclonal antibody targeting the ECD of CD147. Treatment with 30μg/mL anti-CD147 significantly reduced PR8 adsorption on A549 cells, and the effect increased with higher antibody concentrations (**Figure 8C**). Together, these results suggest that both soluble huCD147-Strep and an-ti-CD147 block influenza virus adsorption to host cells by disrupting the CD147–HA interaction in a dose-dependent manner and significantly reducing virus proliferation at later stages of infection.

## 4 Discussion

### 4.1 CD147 as a novel host factor for influenza virus attachment

The influenza virus HA, a class I fusion protein, plays a central role in viral recognition, attachment, and membrane fusion (Samji, 2009; Borau and Stertz, 2021). The polybasic sequence (GERRRKKR) at the HA cleavage site of H5 subtype avian influenza viruses can be cleaved by host proteases (such as furin), triggering dynamic conformational changes in HA (Spronken et al., 2024). Coupled with technical limitations in membrane protein interaction detection, these factors collectively impede the screening of host interaction proteins. To address this, we mutated this site to a neutral sequence (GETR). This mutation blocks protease cleavage, preserving the intact HA conformation and eliminating structural instability during protein processing. By combining membrane-specific extraction reagents and non-denaturing technologies, we utilized the intact HA as a stable bait to significantly enhance capture efficiency of the host membrane protein interaction network.

In this study, we identified CD147 as a novel host interactor of AIV HA through membrane protein isolation combined with AP-MS (**Figure 1**). CD147, a transmembrane glycoprotein, has been reported to act as a receptor or co-receptor for pathogens such as *Plasmodium* (Zhang et al., 2018), bacteria (dos Santos Souza et al., 2021), and viruses including SARS-CoV-2 (Pushkarsky et al., 2001; Wang K. et al., 2021). Our findings demonstrate that CD147 knockdown significantly inhibits influenza virus attachment in both avian DF1 and human A549 cells (**Figure 4**), while soluble CD147 protein or monoclonal antibodies blocking CD147–HA interaction markedly reduce viral replication (**Figure 8**). BLI assays further reveal that the binding affinity between CD147 and HA (KD ~ 2.0 × 10^-7^–7.0 × 10^-7^ M) far exceeds that between SA and HA (KD ~ 3 mM) (**Figure 7**), suggesting that CD147 may serve as a high-affinity receptor for viral attachment. This discovery challenges the traditional paradigm of SA-dominated influenza virus attachment (Skehel and Wiley, 2000) and highlights the diversified roles of host membrane proteins in viral entry.

### 4.2 Dual roles of CD147 glycosylation

The N-glycosylation of CD147 appears to exert dual regulatory effects. First, Subcellular localization: HG-CD147 is transported to the cell membrane via glycan-mediated trafficking (Bai et al., 2014), directly participating in viral attachment. Virus-induced upregulation of HG-CD147 (**Figures 5A, B**) underscores its functional necessity during infection. Second, Core protein interaction: Mutagenesis removing glycosylation sites confirms that LG-CD147 retains HA-binding capability, with even stronger affinity (**Figures 6D–G**), indicating that CD147’s immunoglobulin-like domains (particularly the HA1 RBD) mediate direct interaction (**Figure 7**). This mechanism mirrors the ACE2–SARS-CoV-2 S protein interaction, where glycosylation regulates receptor localization but does not hinder core binding (Rowland and Brandariz-Nuñez, 2021).

AlphaFold 3 predictions suggest proximity between the N186 glycosylation site of CD147 and HA-Q175 (**Figures 7A–E**), indicating that glycans may indirectly enhance viral attachment via steric or charge effects rather than direct binding. This finding contrasts with Neisseria meningitidis, which exploits CD147 glycans (Asn186-sialylated tri-antennary N-glycans) for endothelial colonization (Bernard et al., 2014), underscoring the diversity of pathogen–host interaction mechanisms.

### 4.3 Glycosylation-driven “two-step” viral entry strategy

We propose a sequential model in which influenza virus exploits the glycan landscape for optimal docking. First, Low-affinity scan: SA receptors facilitate viral enrichment on cell surfaces (Lehmann et al., 2005), with HA and neuraminidase (NA) synergistically promoting directional viral movement (Steele et al., 2021). Second, High-affinity lock-in: core proteins, such CD147, establish stable attachment points, while glycans may optimize the binding interfaces via conformational adjustments or electrostatic modulation (Li et al., 2024). This hierarchical use of glycan-mediated interactions maximizes entry efficiency while minimizing premature release, offering a rational framework for glyco-targeted antivirals.

### 4.4 Therapeutic implications and future directions

CD147’s broad-spectrum antiviral activity and its role in cross-species transmission position it as a promising therapeutic target to block influenza virus entry. The anti-CD147 monoclonal antibody meplazumab, already shown to suppress SARS-CoV-2 infection, now emerges as a repurposable therapeutic for influenza (Geng et al., 2021), and our findings extend its potential application to influenza virus inhibition (**Figure 8**). Future studies should focus on validating CD147-targeted strategies in animal models and elucidating the dynamic mechanism of the CD147–HA, which could guide small-molecule inhibitor or vaccine design. Additionally, interspecies functional differences in CD147 could serve as predictive biomarkers for predicting zoonotic risk of emerging influence viruses.

A limitation of this study is that all findings were based on *in vitro* experiments; therefore, further *in vivo* studies are necessary to validate the role of CD147 in influenza virus infection and cross-species transmission.

## 5 Conclusions

The host membrane protein CD147 mediates the adsorption of influenza virus on the host cell surface by directly interacting with the head domain RBD of the viral HA protein through its extracellular immunoglobulin-like structure. This interaction facilitates viral attachment, enhances subsequent infection, and promotes viral proliferation within host cells. In summary, CD147 emerges as a critical host factor in influenza virus pathogenesis, offering new avenues for antiviral development and insights into viral adaptation across species barriers.

## Data Availability

The datasets presented in this study can be found in online repositories. The names of the repository/repositories and accession numbers can be found in the article/**Supplementary Material**.
