# Supplementary figures and images for: CD147 mediates the adsorption of influenza A virus on the cell surface through direct interaction with HA (part 1 of 2)
Source: Front Cell Infect Microbiol. 2025 Aug 29;15:1647283. doi: 10.3389/fcimb.2025.1647283 (PMC12426278; doi:10.3389/fcimb.2025.1647283)

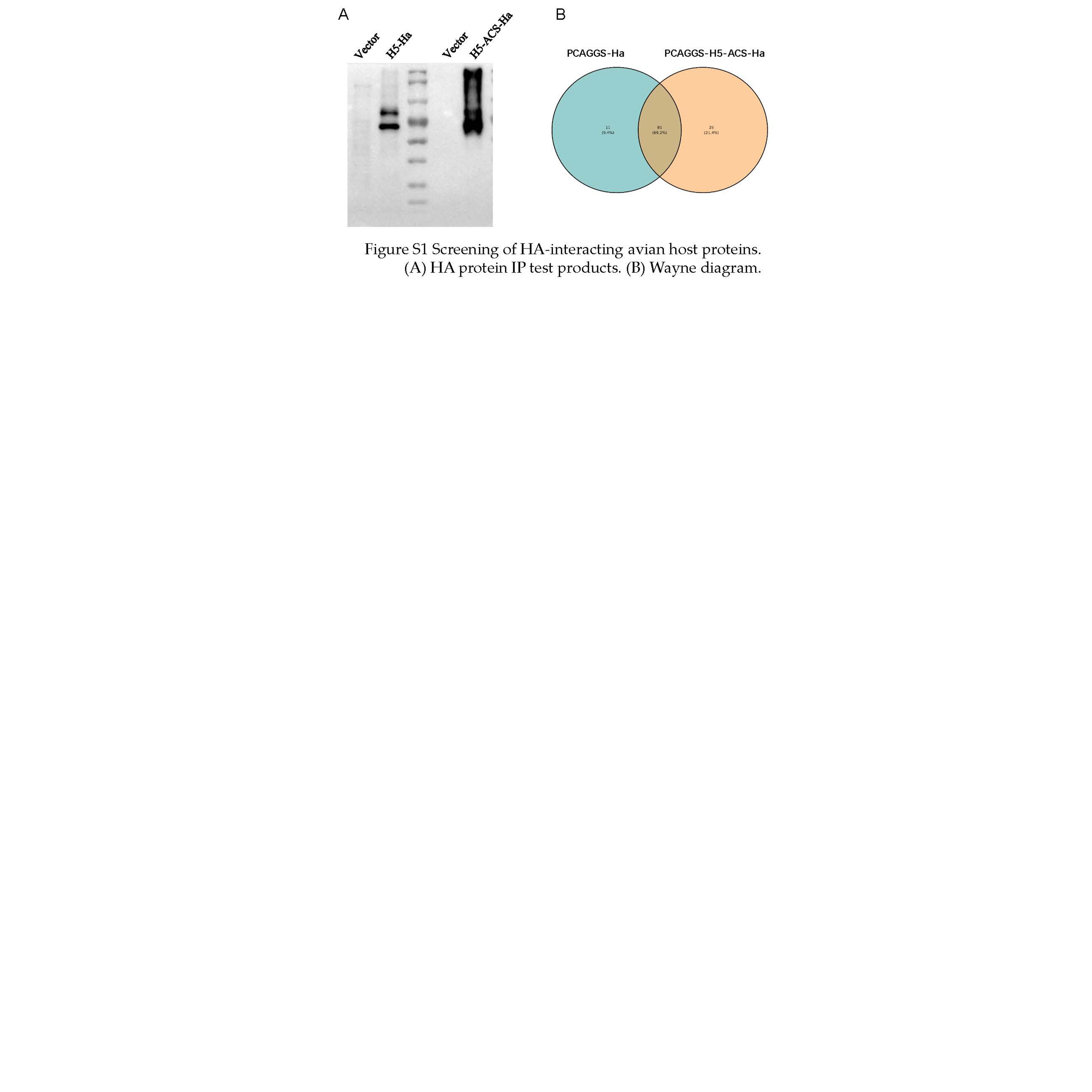

Supplement: Supplementary file 1 [file Image1.jpeg]

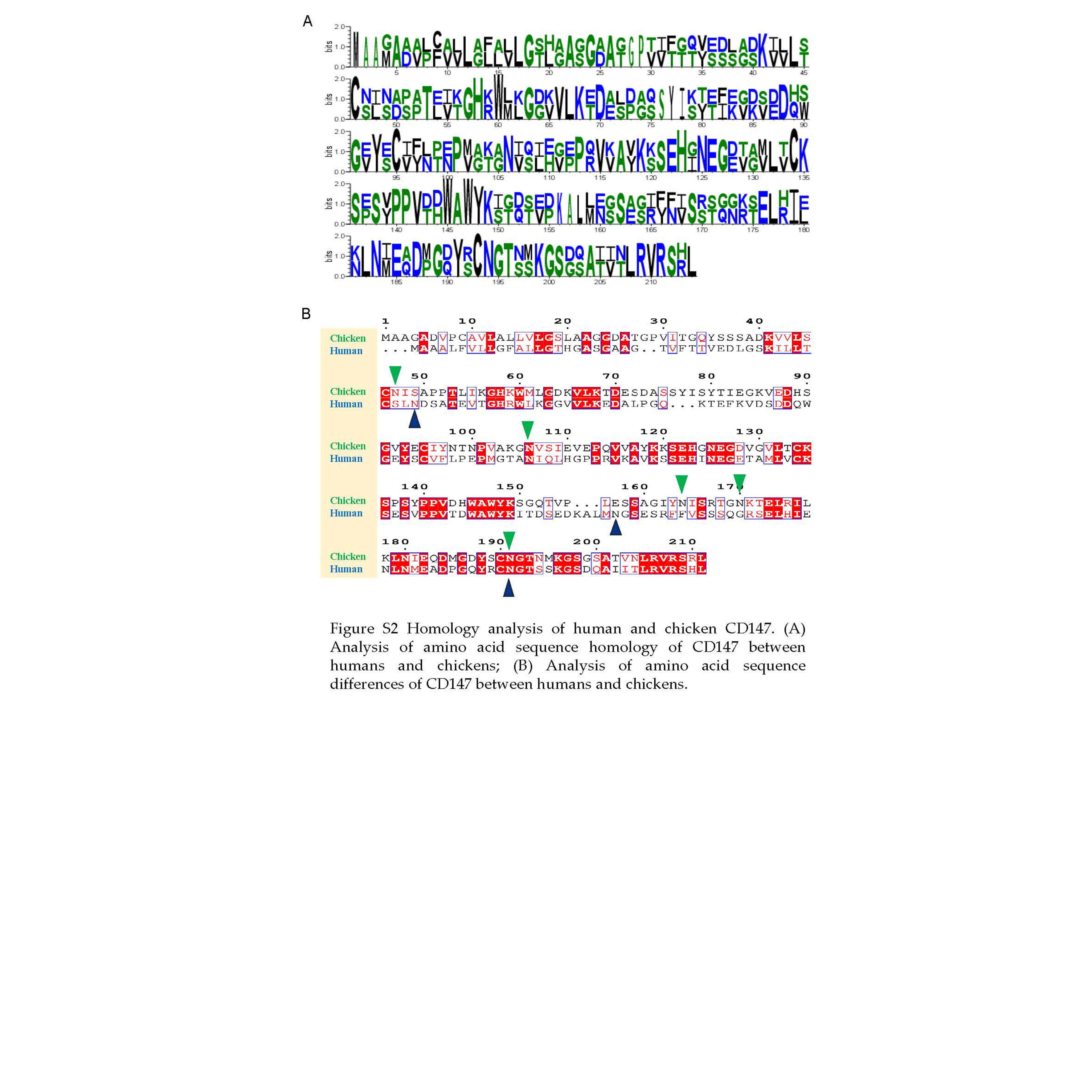

Supplement: Supplementary file 2 [file Image2.jpeg]

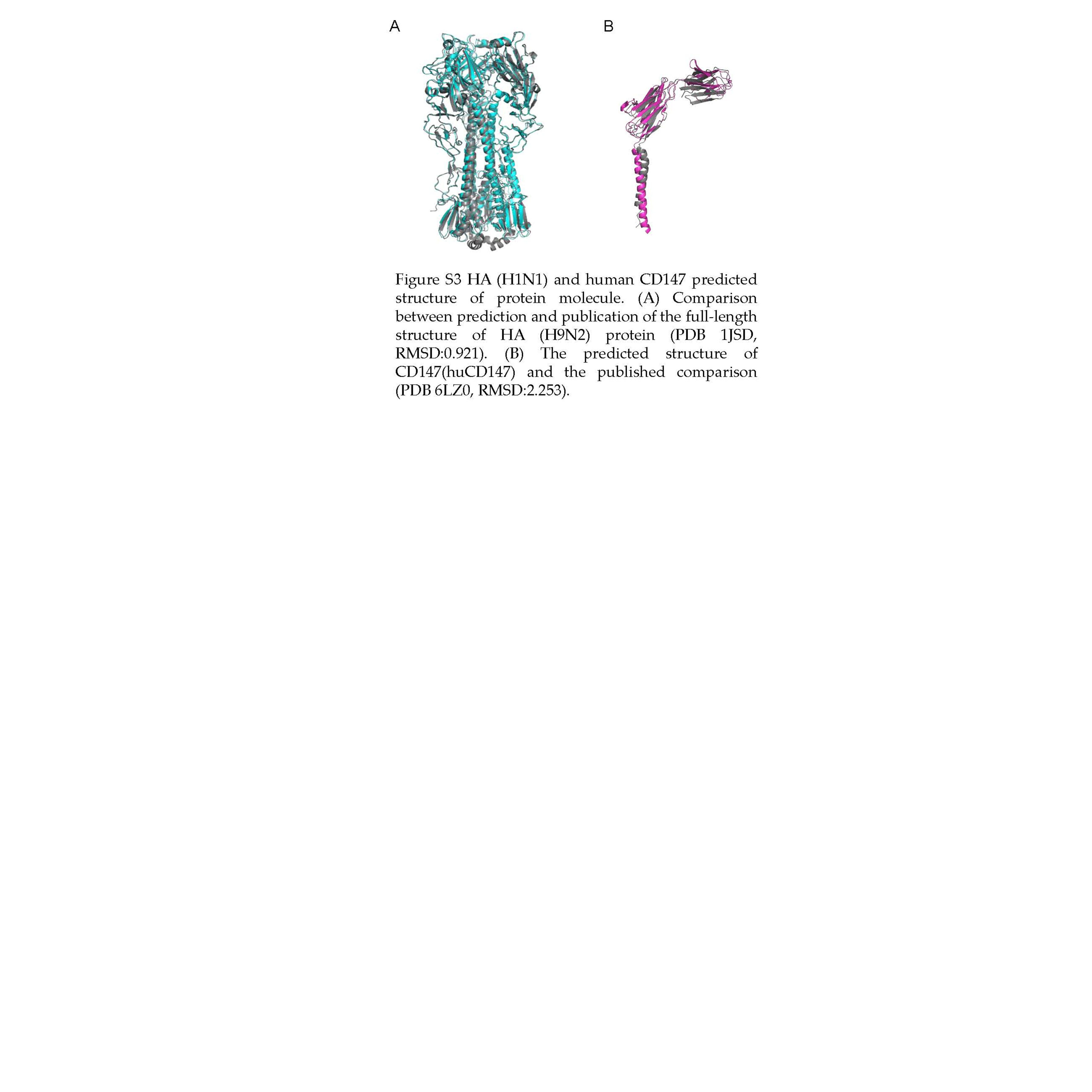

Supplement: Supplementary file 3 [file Image3.jpeg]

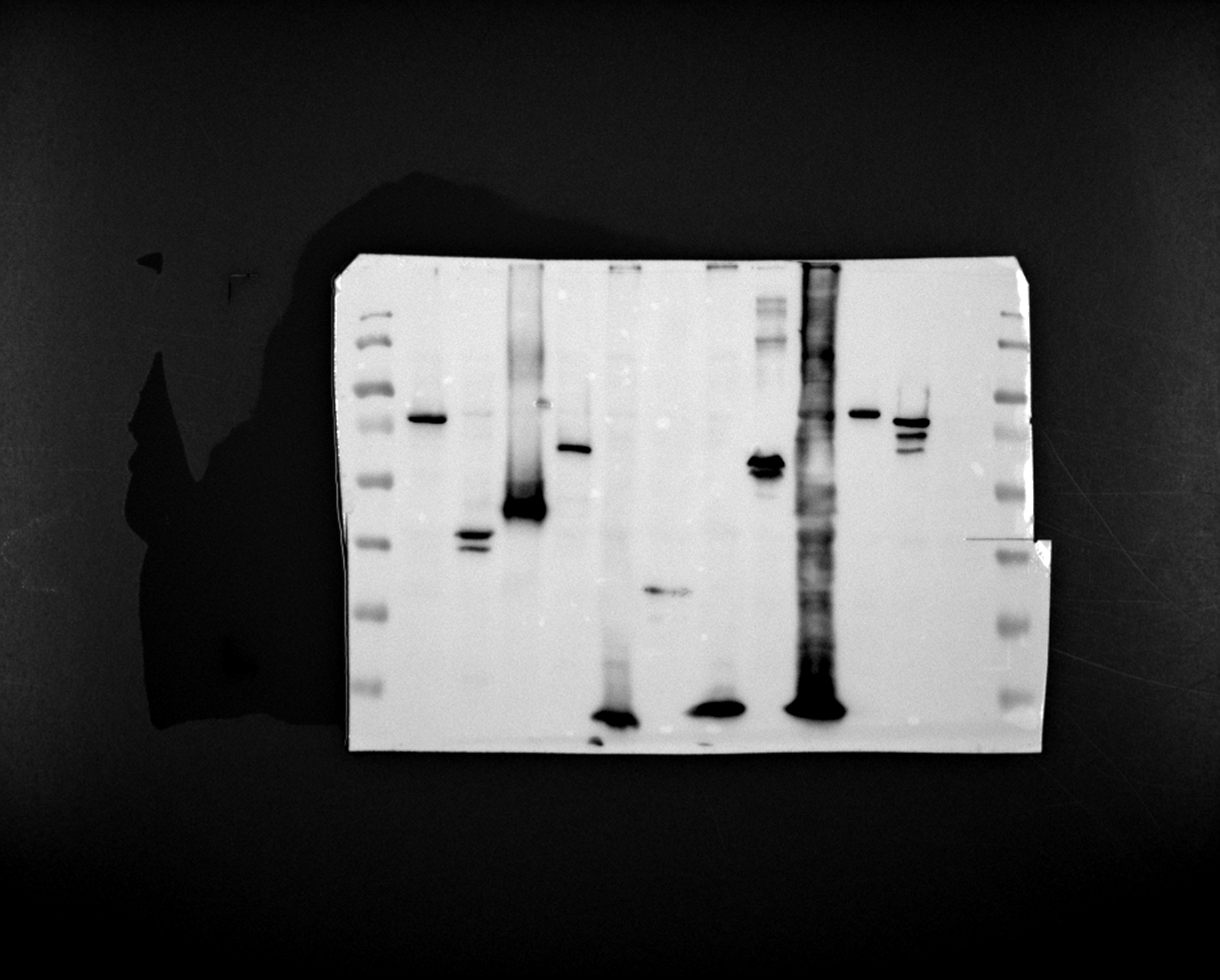

Supplement: Supplementary file 7 [file DataSheet1.zip › raw data-1/Figure 1/Fingure 1C/FLAG 1.Tif]

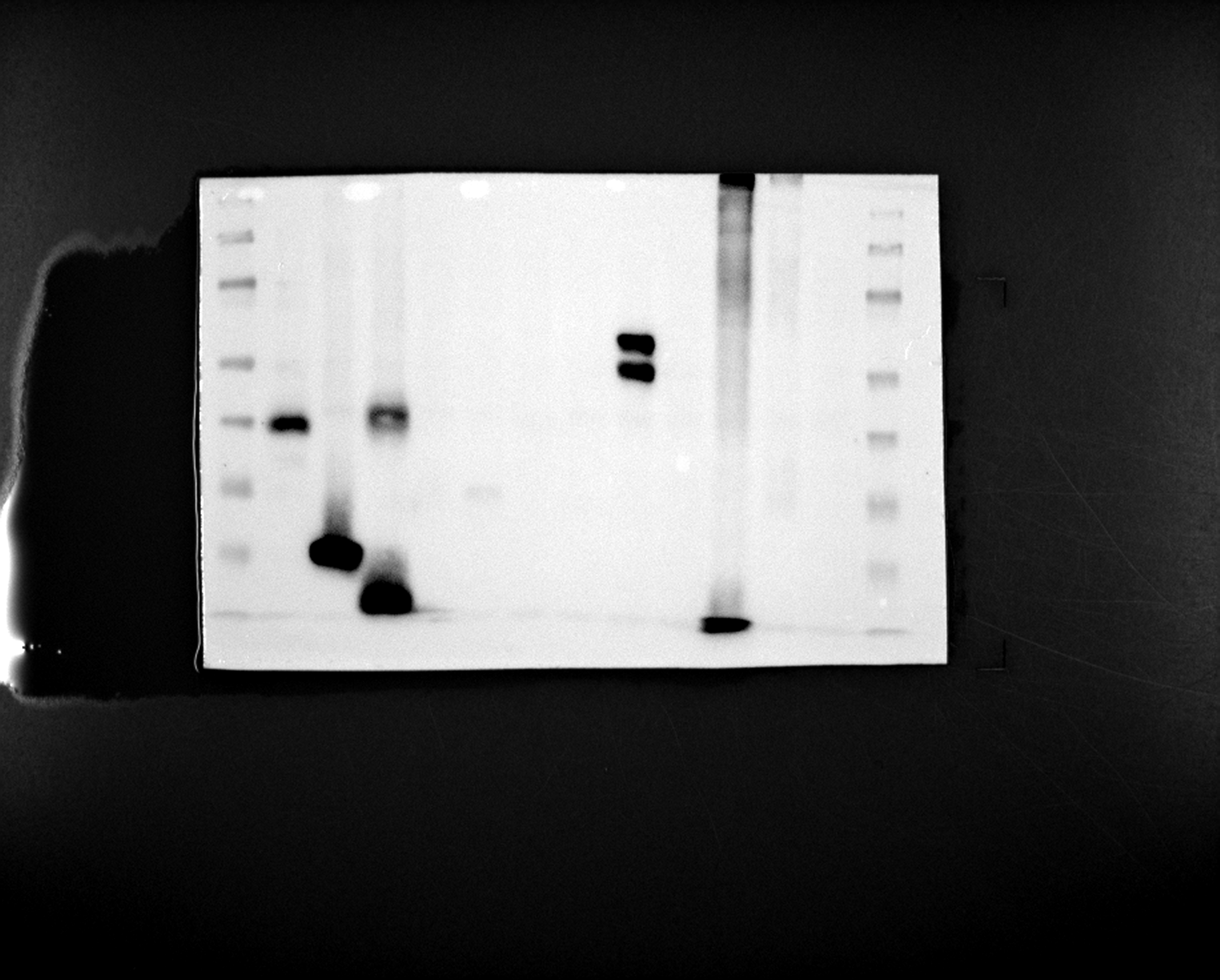

Supplement: Supplementary file 7 [file DataSheet1.zip › raw data-1/Figure 1/Fingure 1C/FLAG 2.Tif]

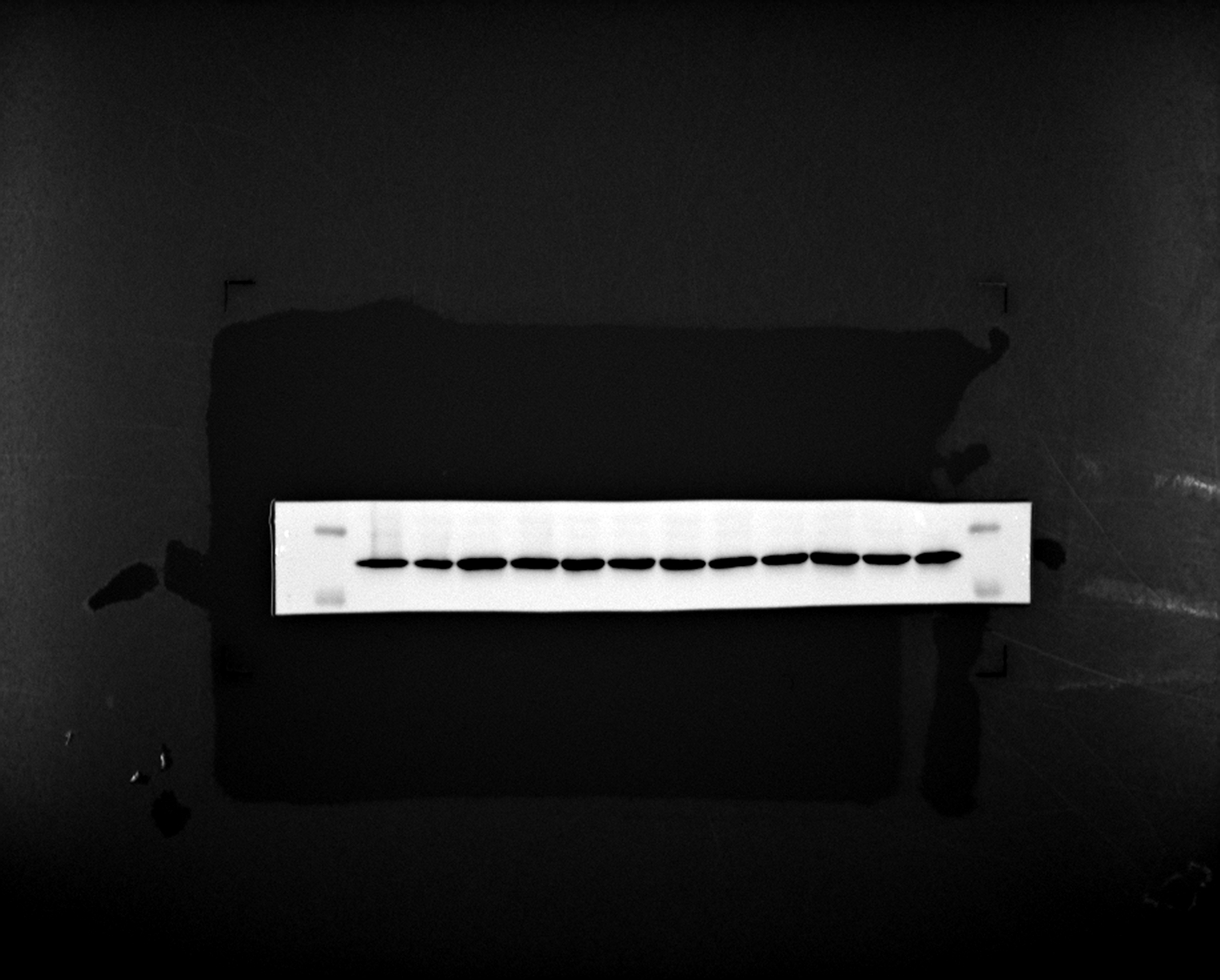

Supplement: Supplementary file 7 [file DataSheet1.zip › raw data-1/Figure 1/Fingure 1C/GAPDH 1.Tif]

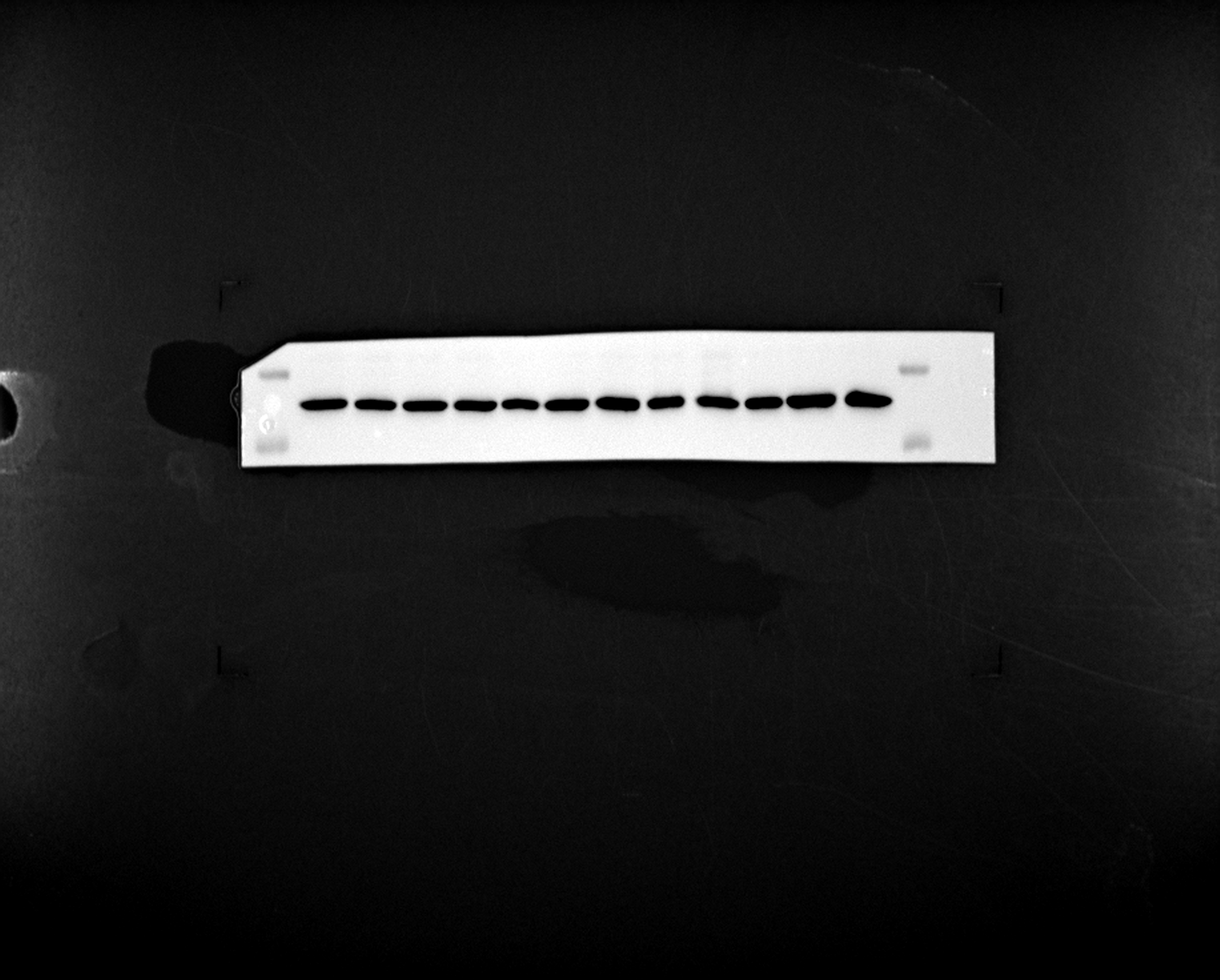

Supplement: Supplementary file 7 [file DataSheet1.zip › raw data-1/Figure 1/Fingure 1C/GAPDH 2.Tif]

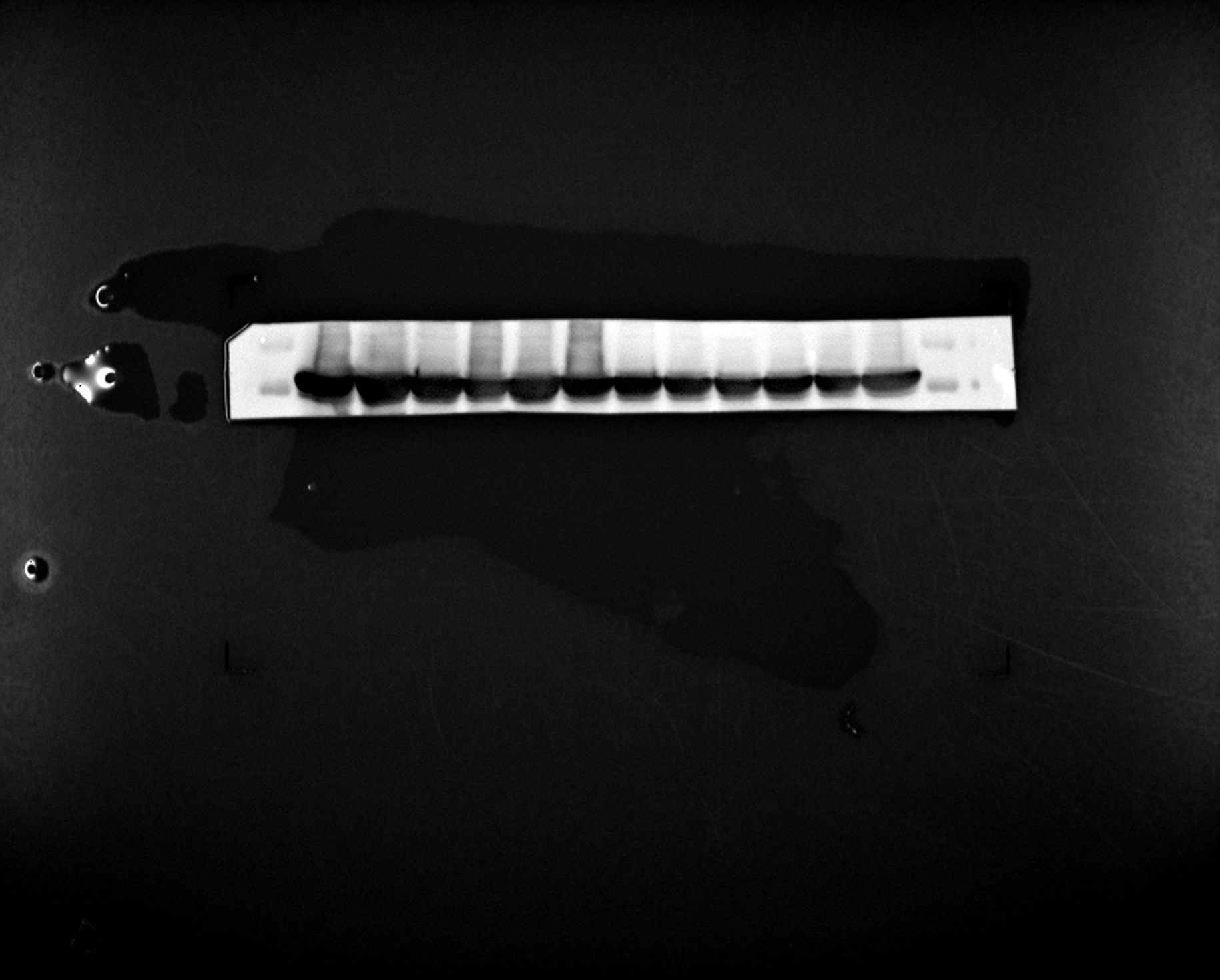

Supplement: Supplementary file 7 [file DataSheet1.zip › raw data-1/Figure 1/Fingure 1C/NP 1.Tif]

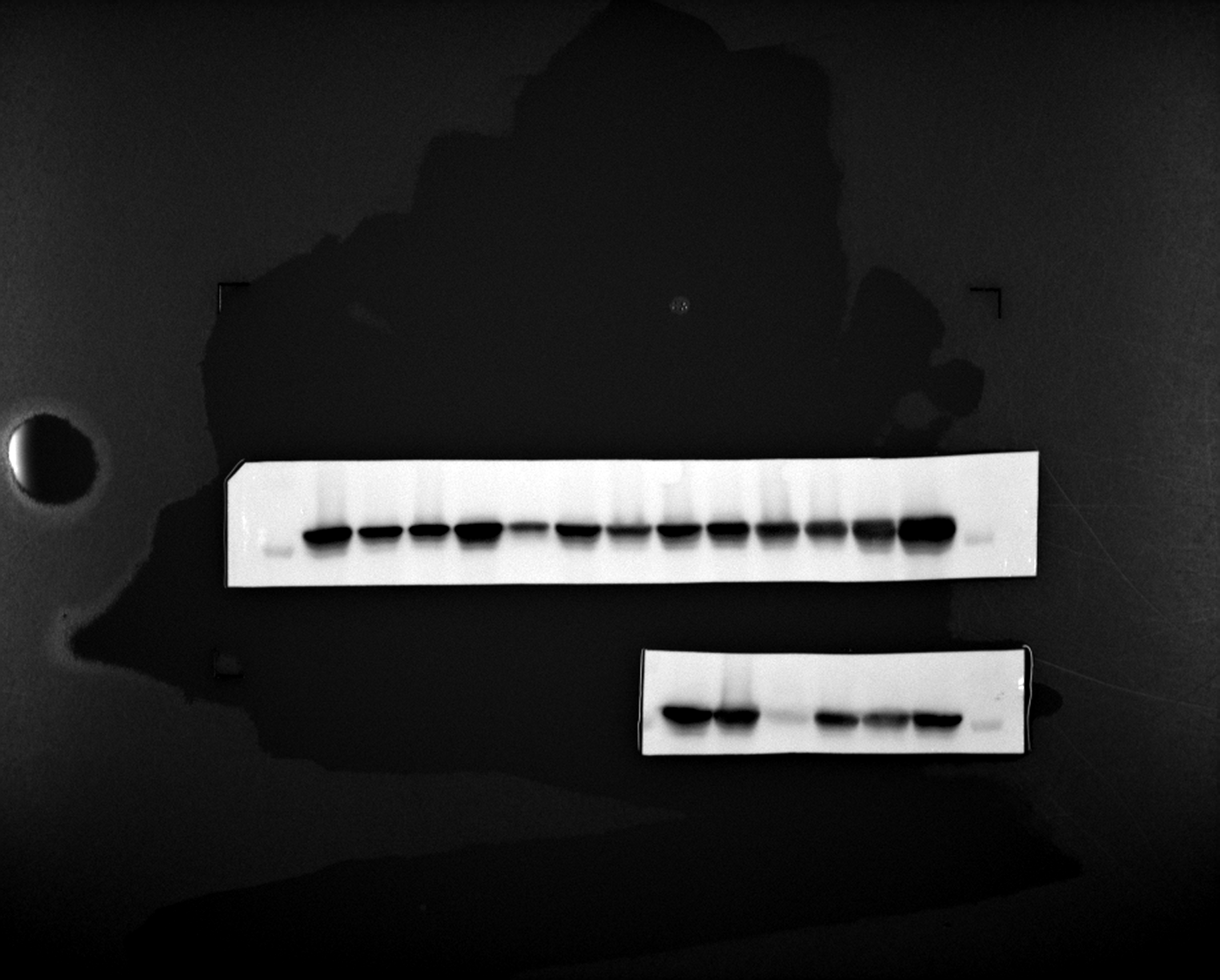

Supplement: Supplementary file 7 [file DataSheet1.zip › raw data-1/Figure 1/Fingure 1C/NP 2.Tif]

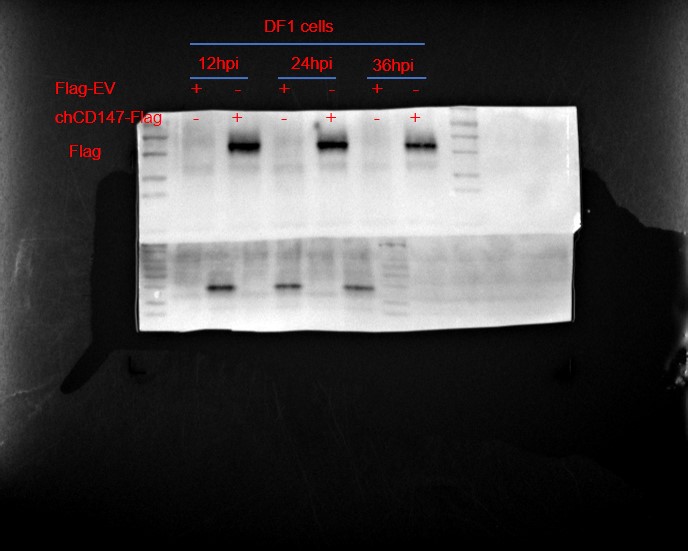

Supplement: Supplementary file 7 [file DataSheet1.zip › raw data-1/Figure 2/2-B DF1-CD147-JX/Flag.jpg]

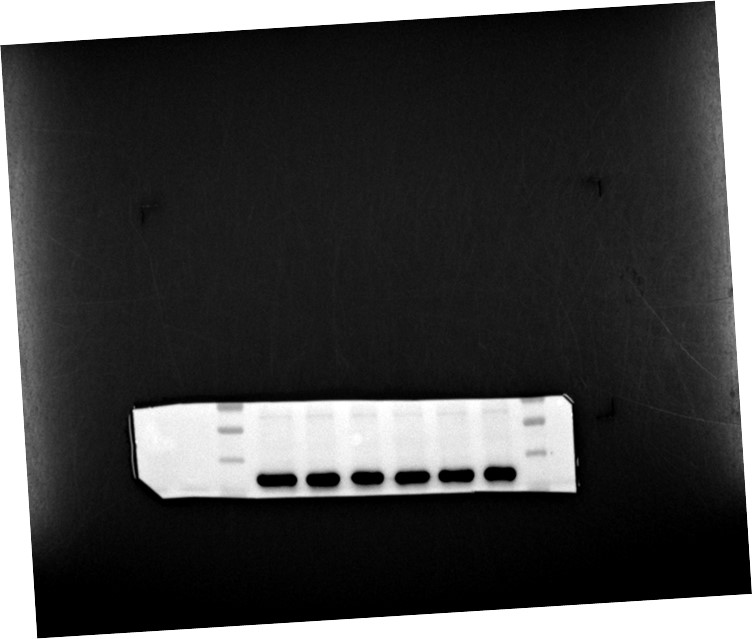

Supplement: Supplementary file 7 [file DataSheet1.zip › raw data-1/Figure 2/2-B DF1-CD147-JX/gapdh.jpg]

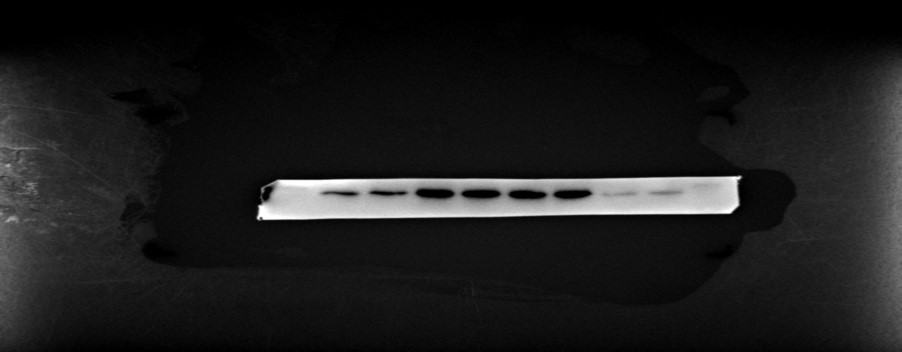

Supplement: Supplementary file 7 [file DataSheet1.zip › raw data-1/Figure 2/2-B DF1-CD147-JX/NP.jpg]

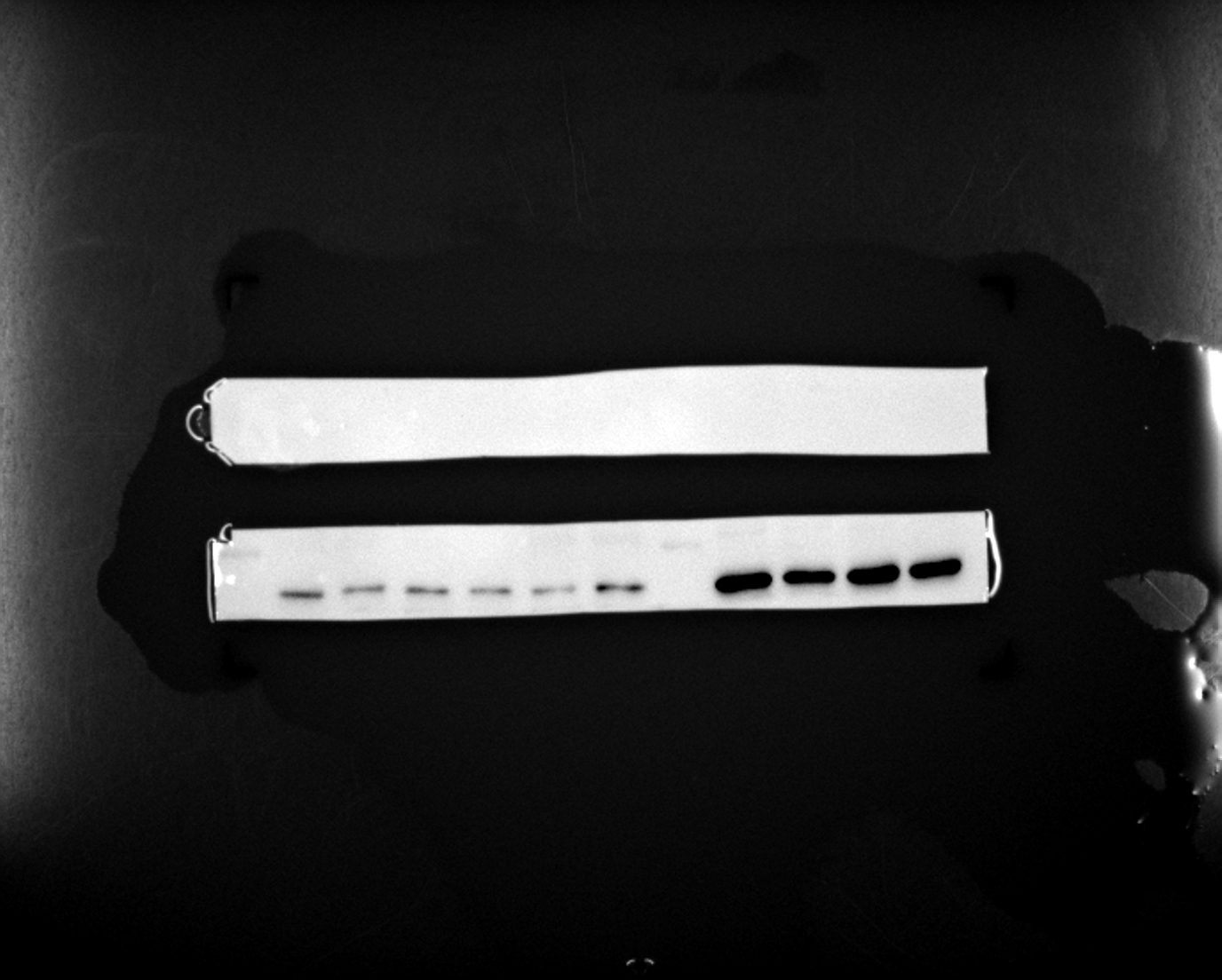

Supplement: Supplementary file 7 [file DataSheet1.zip › raw data-1/Figure 2/2-D DF1-Si/A549-HB2-4 37=gapdh.jpg]

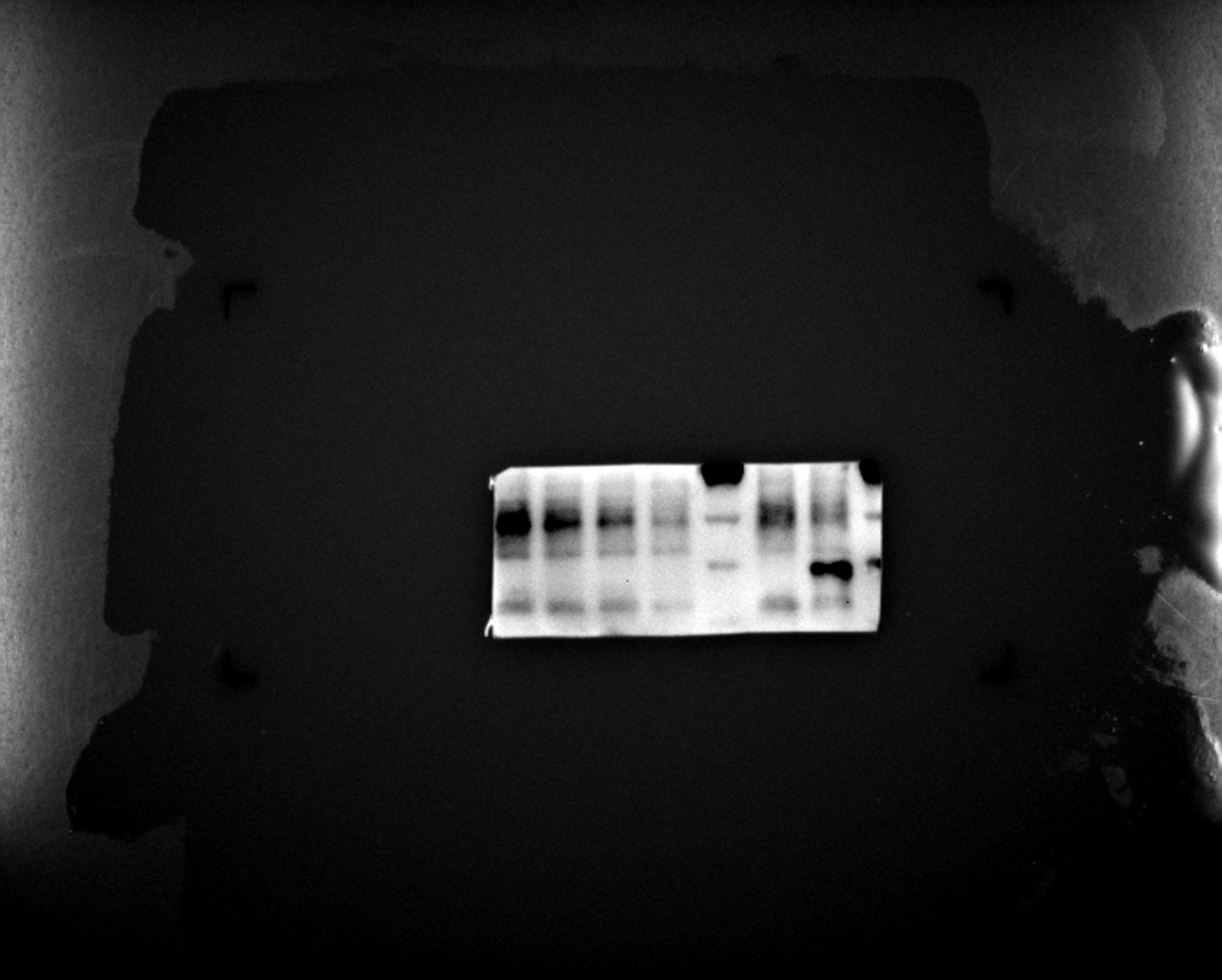

Supplement: Supplementary file 7 [file DataSheet1.zip › raw data-1/Figure 2/2-D DF1-Si/A549.jpg]

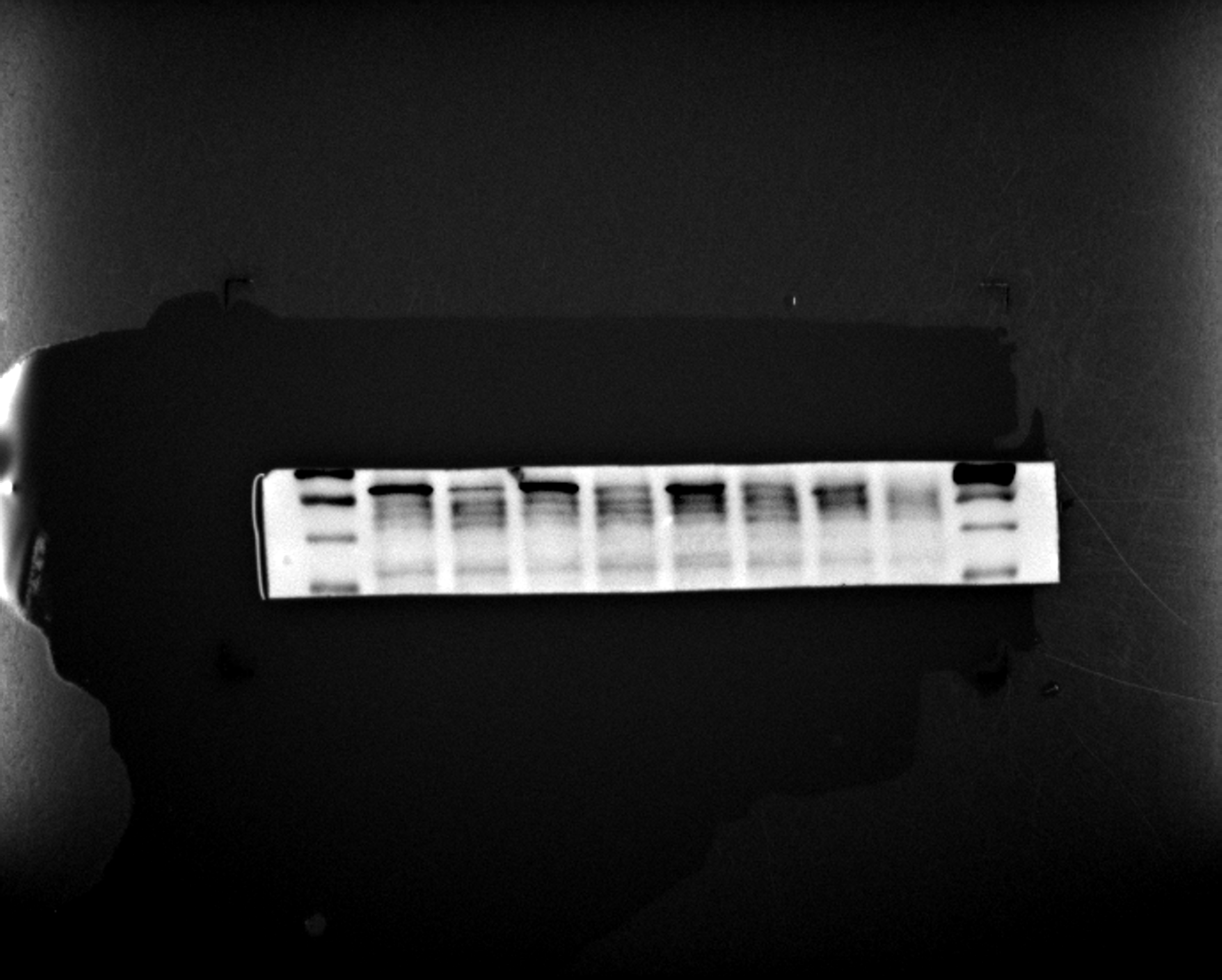

Supplement: Supplementary file 7 [file DataSheet1.zip › raw data-1/Figure 2/2-F DF1-siRNA-CD147-JX/DF1-siRNA-CD147-JX-CD147.Tif]

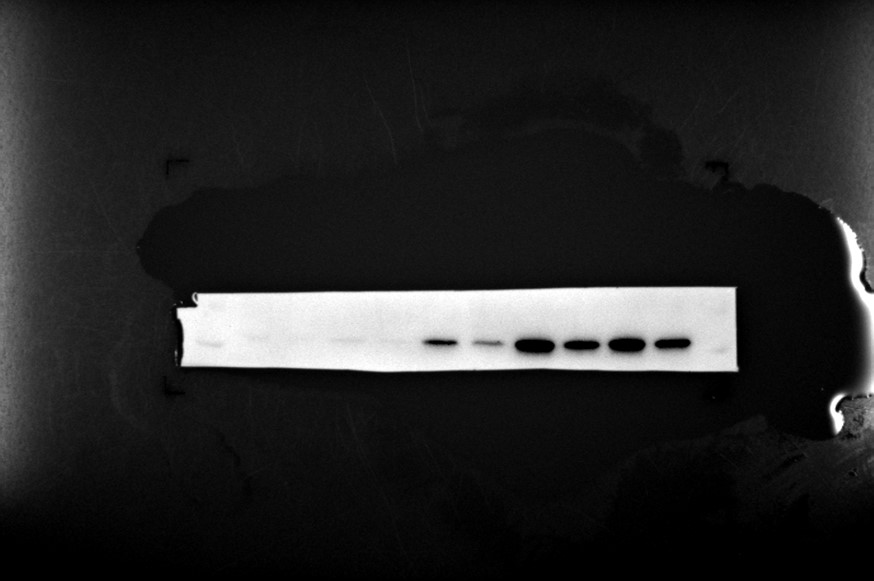

Supplement: Supplementary file 7 [file DataSheet1.zip › raw data-1/Figure 2/2-F DF1-siRNA-CD147-JX/NP.jpg]

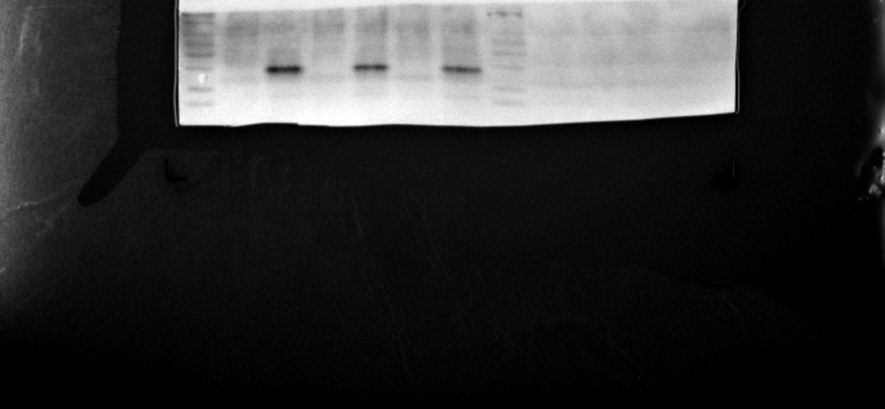

Supplement: Supplementary file 7 [file DataSheet1.zip › raw data-1/Figure 2/2-I A549-CD147-PR8/Flag.jpg]

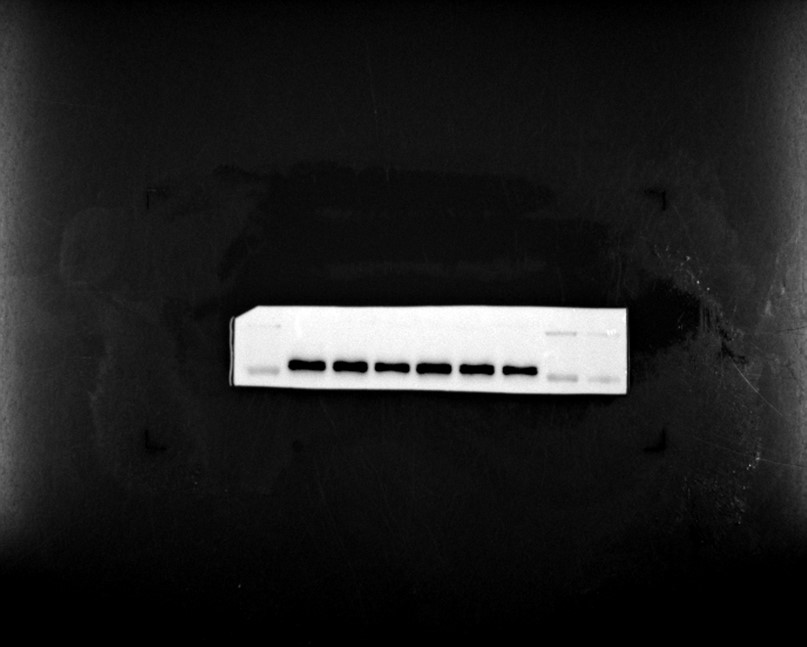

Supplement: Supplementary file 7 [file DataSheet1.zip › raw data-1/Figure 2/2-I A549-CD147-PR8/Gapdh.jpg]

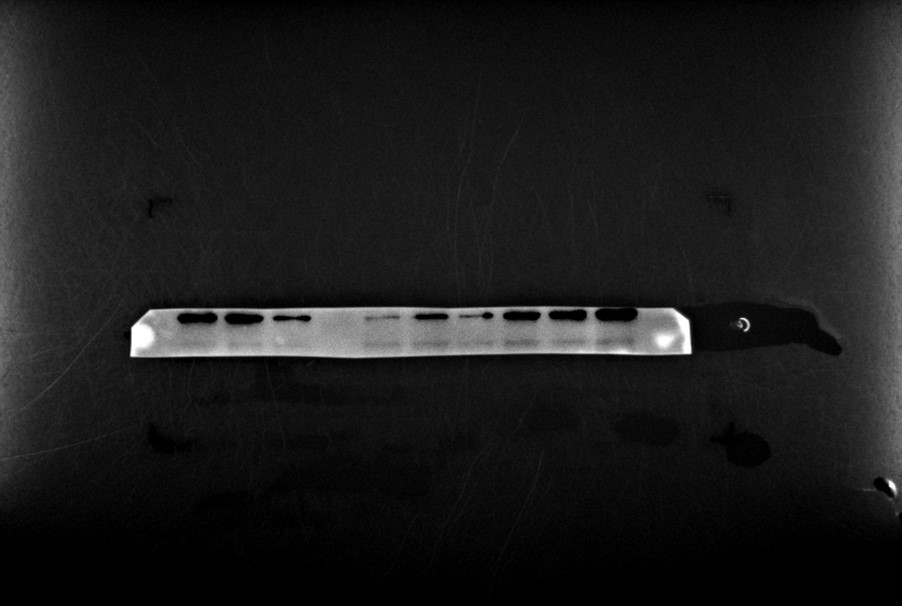

Supplement: Supplementary file 7 [file DataSheet1.zip › raw data-1/Figure 2/2-I A549-CD147-PR8/NP.jpg]

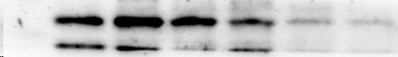

Supplement: Supplementary file 7 [file DataSheet1.zip › raw data-1/Figure 2/2-J A549-CD147-KO/CD147.jpg]

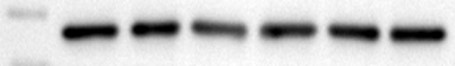

Supplement: Supplementary file 7 [file DataSheet1.zip › raw data-1/Figure 2/2-J A549-CD147-KO/Gapdh.jpg]

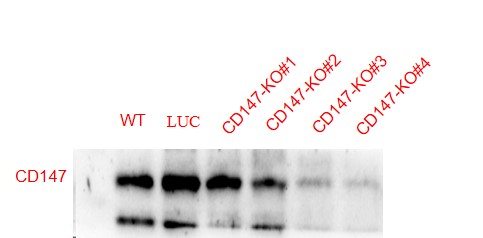

Supplement: Supplementary file 7 [file DataSheet1.zip › raw data-1/Figure 2/2-J A549-KO/CD147.jpg]

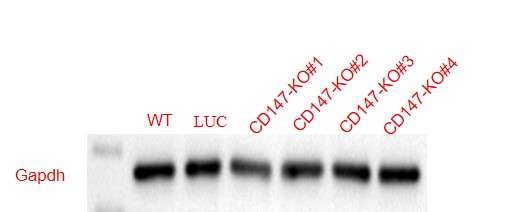

Supplement: Supplementary file 7 [file DataSheet1.zip › raw data-1/Figure 2/2-J A549-KO/Gapdh.jpg]

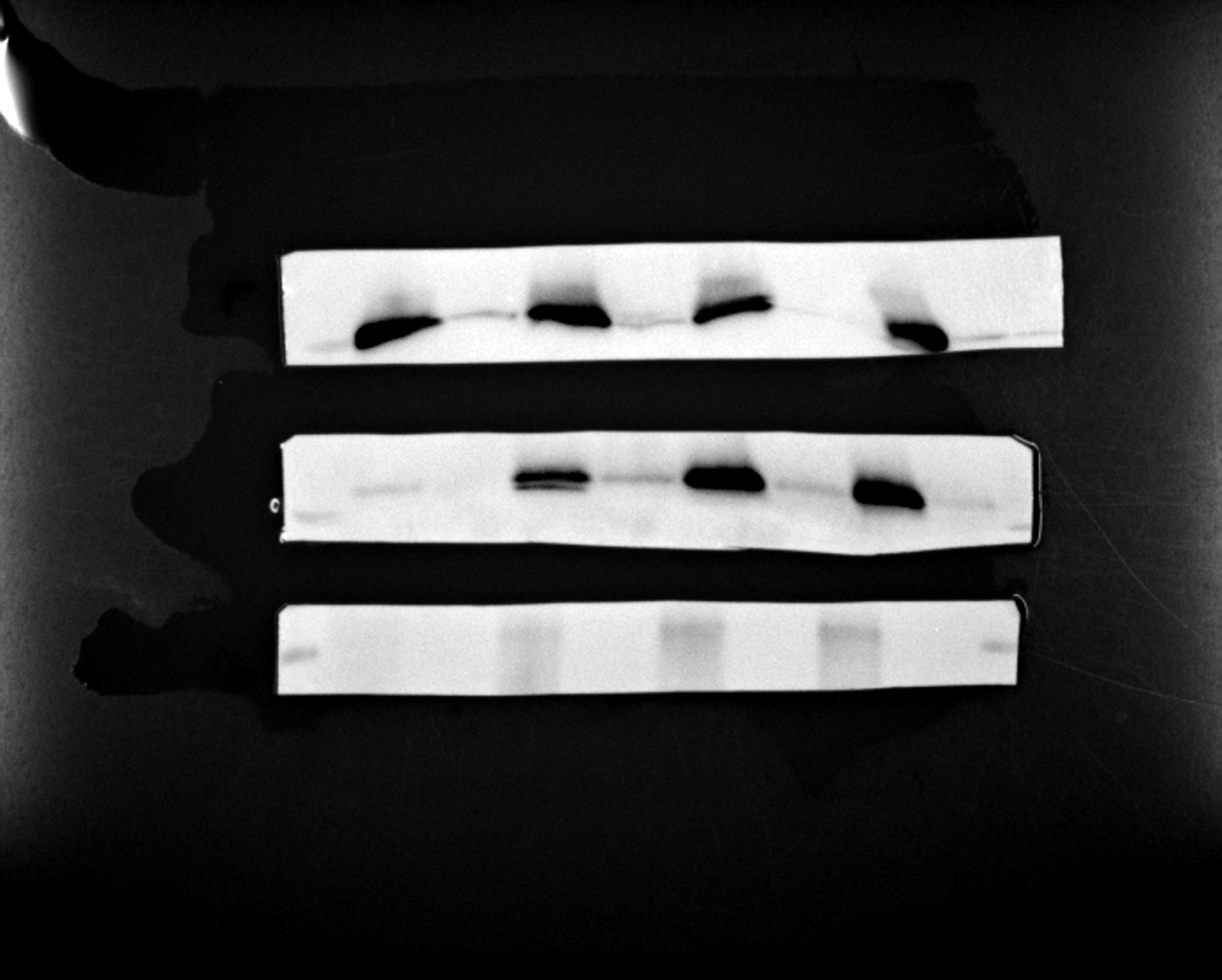

Supplement: Supplementary file 7 [file DataSheet1.zip › raw data-1/Figure 2/2-L A549-CD147-KO-PR8/A549-KB-2-105-PR8-CD147.jpg]

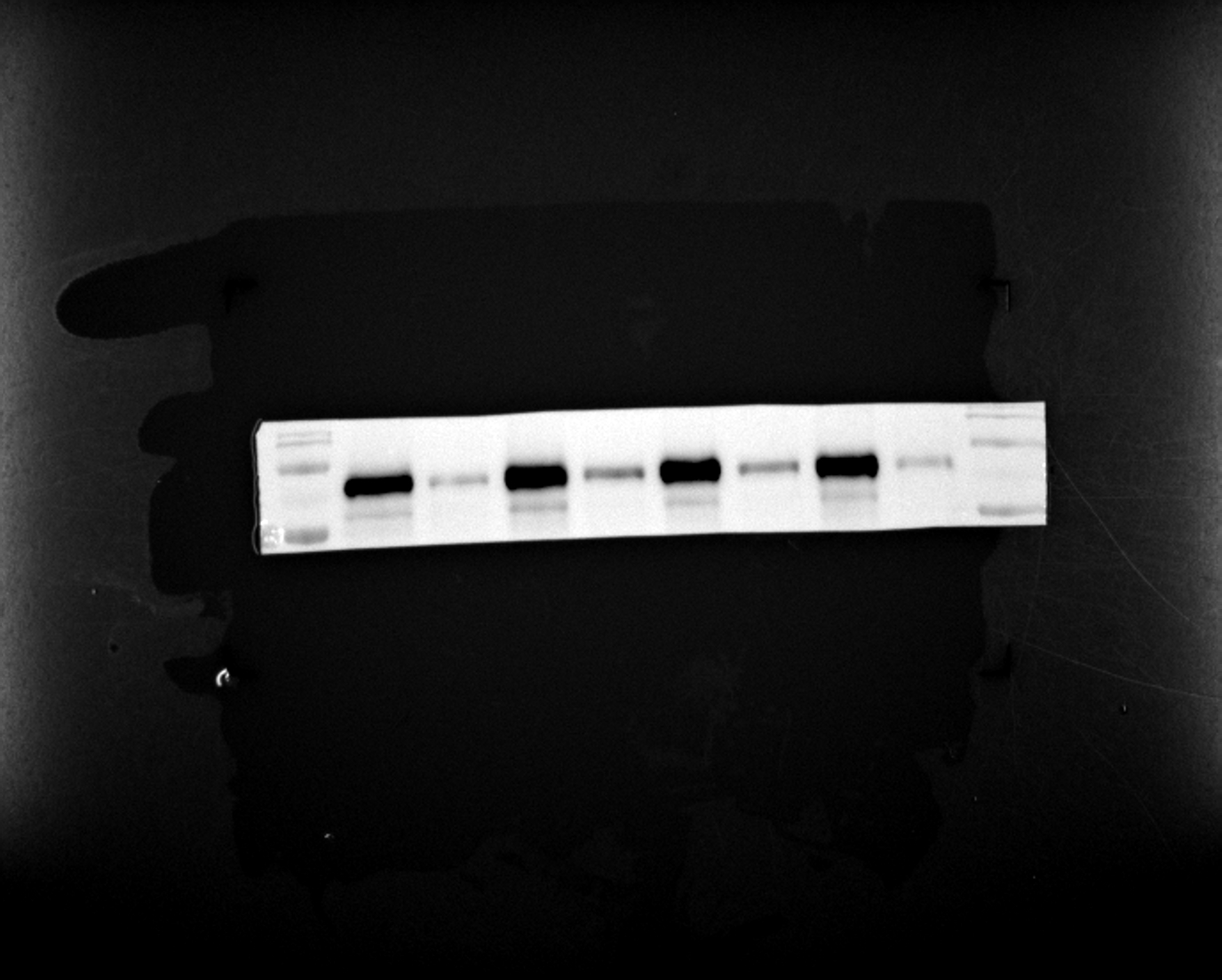

Supplement: Supplementary file 7 [file DataSheet1.zip › raw data-1/Figure 2/2-L A549-CD147-KO-PR8/A549-KB-2-105-PR8-HA.jpg]

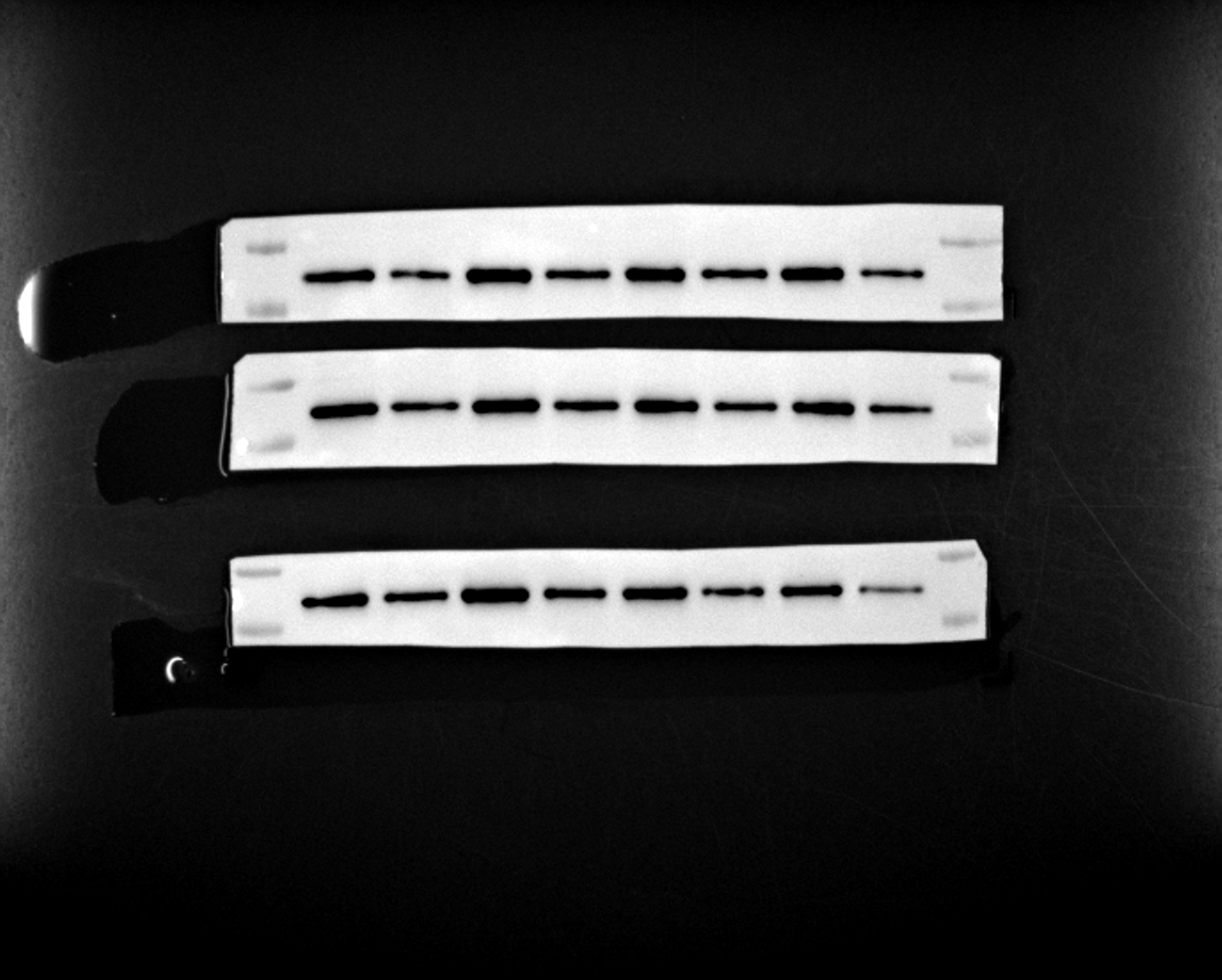

Supplement: Supplementary file 7 [file DataSheet1.zip › raw data-1/Figure 2/2-L A549-CD147-KO-PR8/A549-KB-2-105-PR8-JX GAPDH.jpg]

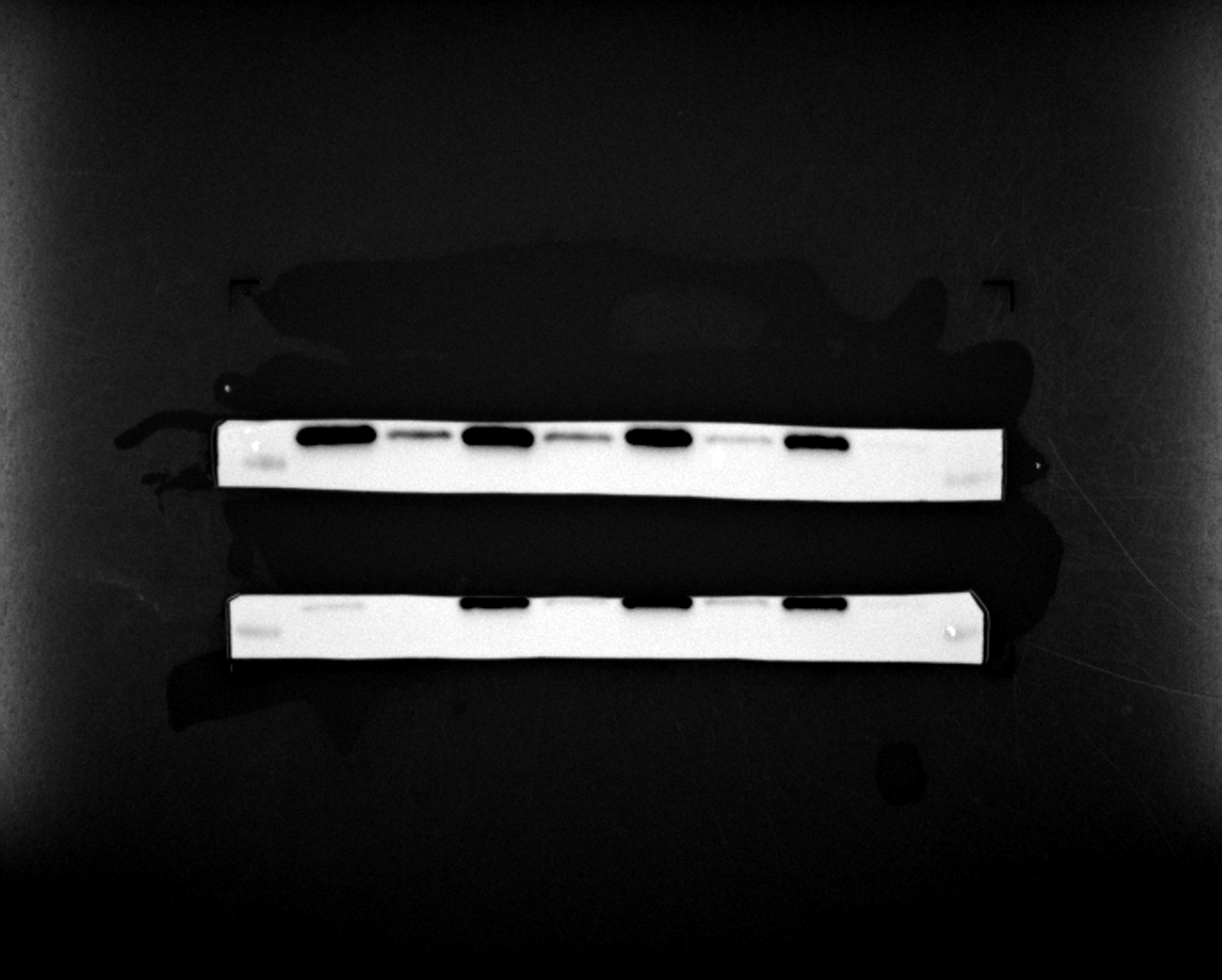

Supplement: Supplementary file 7 [file DataSheet1.zip › raw data-1/Figure 2/2-L A549-CD147-KO-PR8/A549-KB-2-105-PR8-M1.jpg]

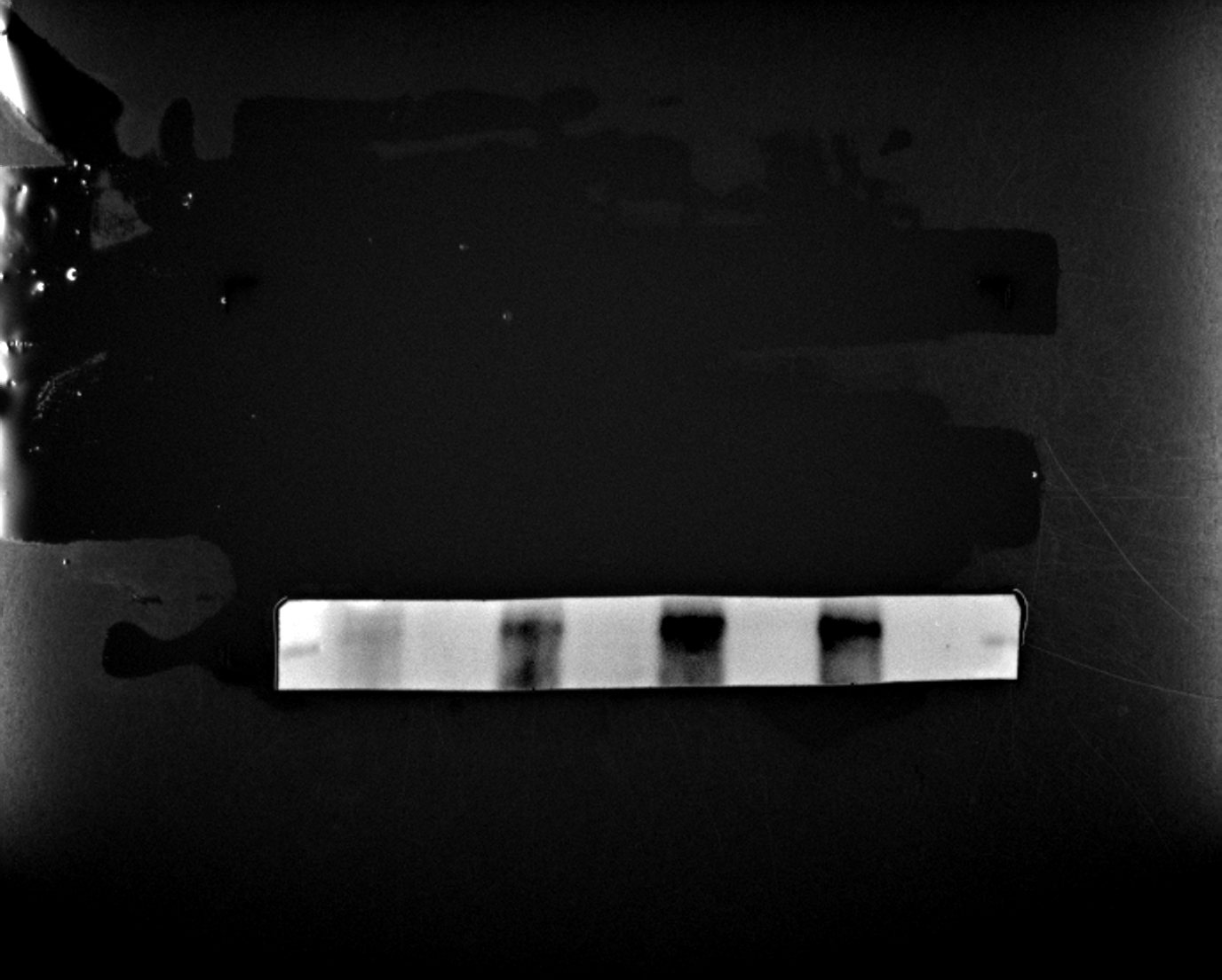

Supplement: Supplementary file 7 [file DataSheet1.zip › raw data-1/Figure 2/2-L A549-CD147-KO-PR8/A549-KB-2-105-PR8-M2.jpg]

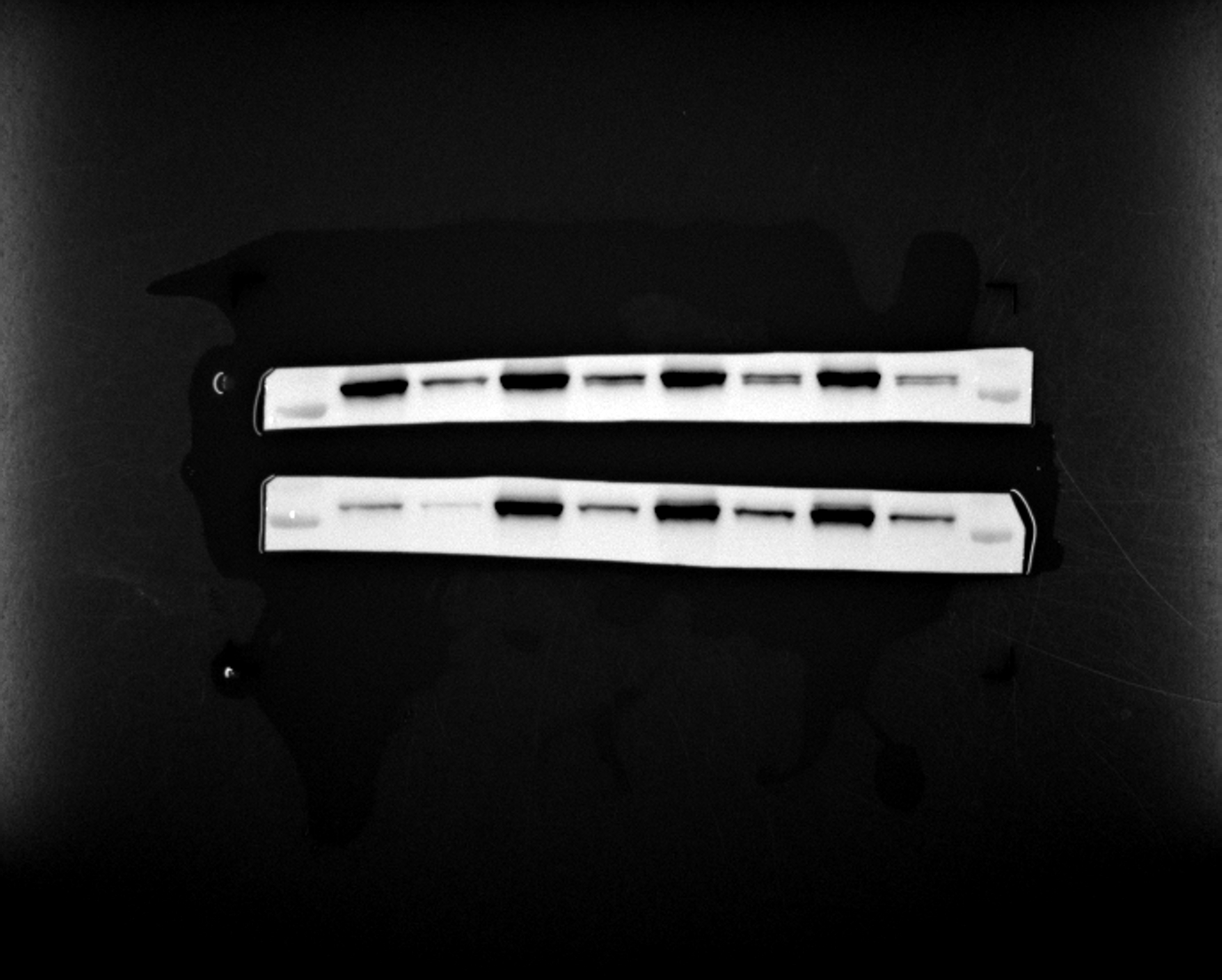

Supplement: Supplementary file 7 [file DataSheet1.zip › raw data-1/Figure 2/2-L A549-CD147-KO-PR8/A549-KB-2-105-PR8-NP-2.jpg]

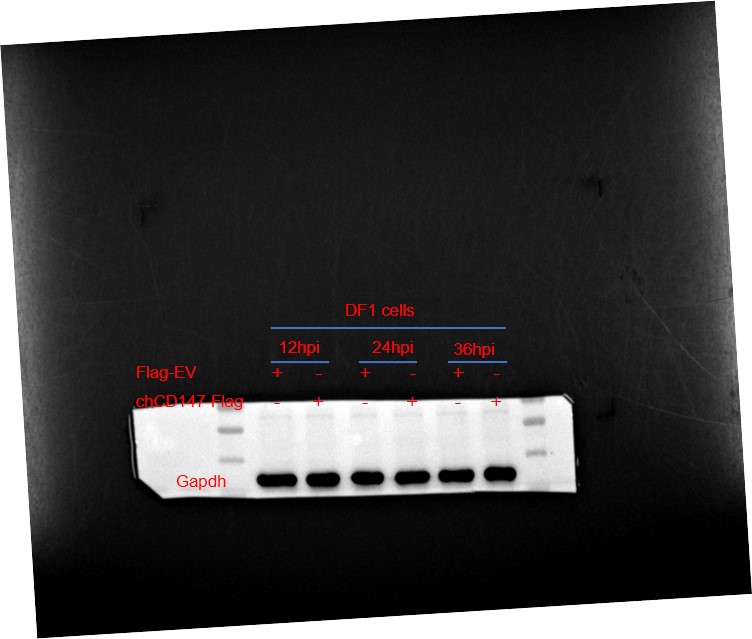

Supplement: Supplementary file 7 [file DataSheet1.zip › raw data-1/Figure 2/Figure 2B/Gapdh.jpg]

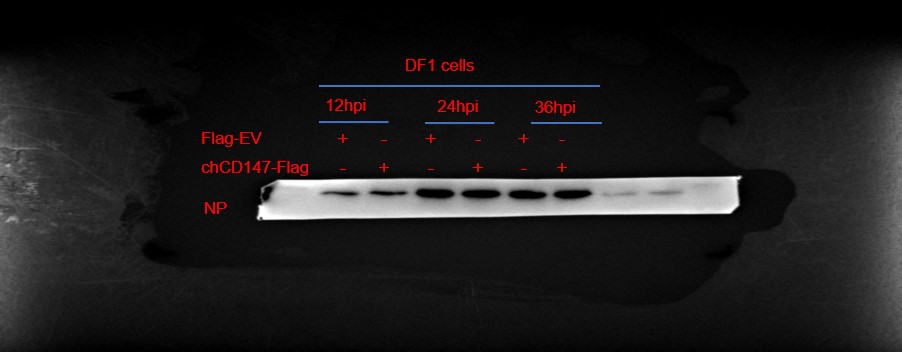

Supplement: Supplementary file 7 [file DataSheet1.zip › raw data-1/Figure 2/Figure 2B/NP.jpg]

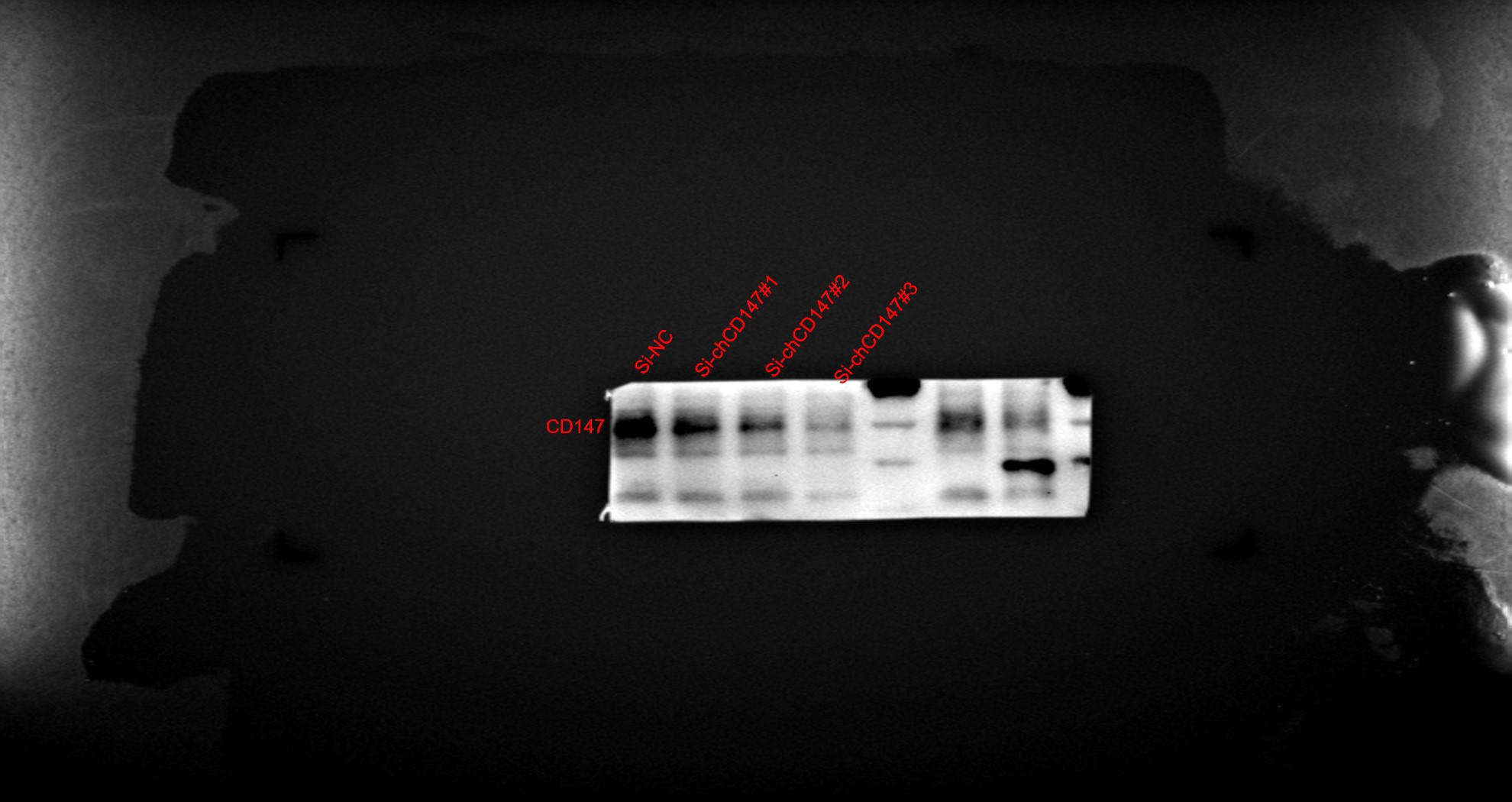

Supplement: Supplementary file 7 [file DataSheet1.zip › raw data-1/Figure 2/Figure 2D/CD147.jpg]

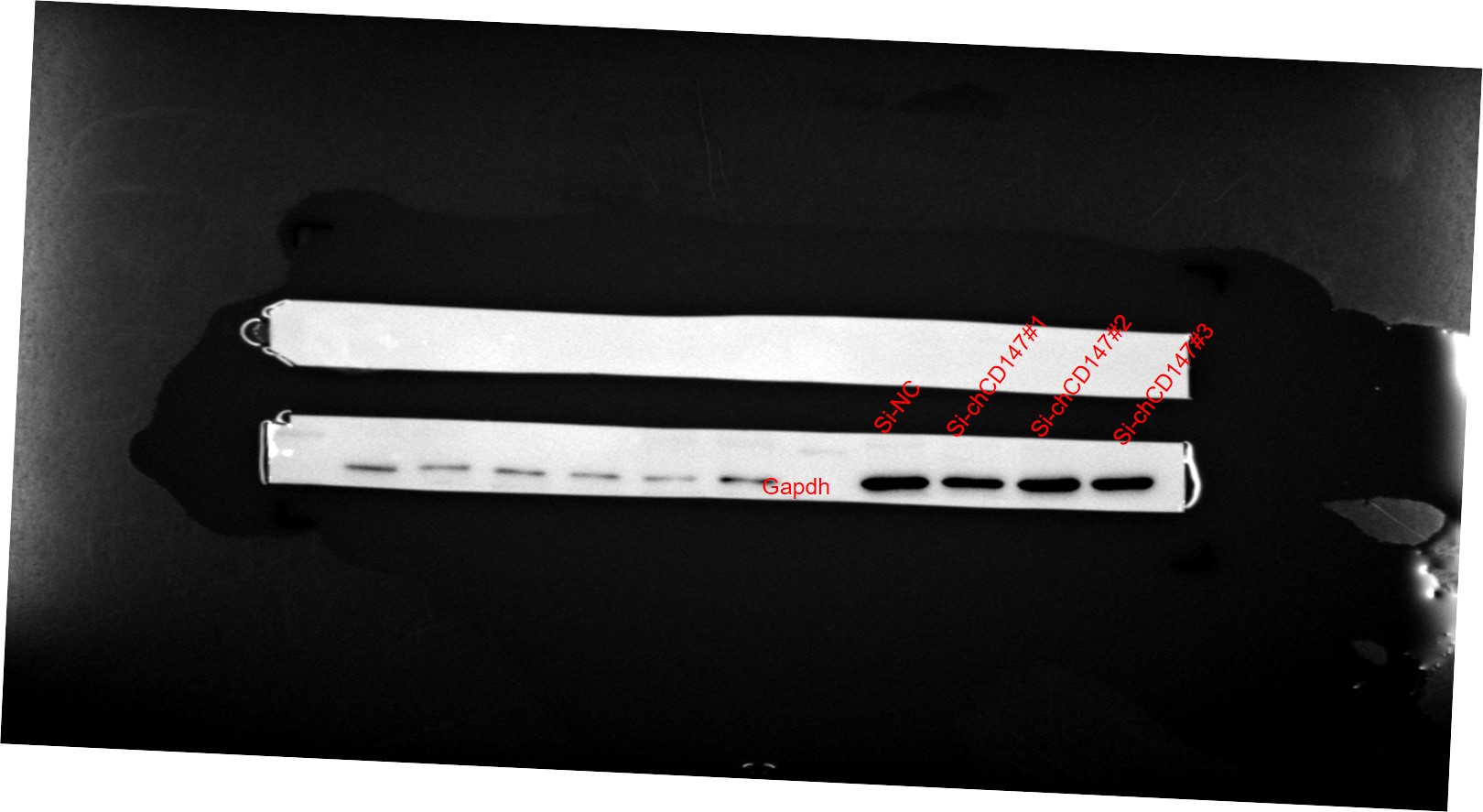

Supplement: Supplementary file 7 [file DataSheet1.zip › raw data-1/Figure 2/Figure 2D/Gapdh.jpg]

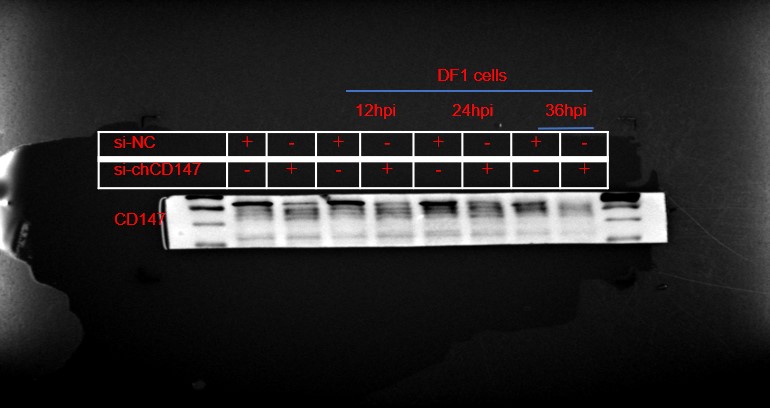

Supplement: Supplementary file 7 [file DataSheet1.zip › raw data-1/Figure 2/Figure 2F/CD147.jpg]

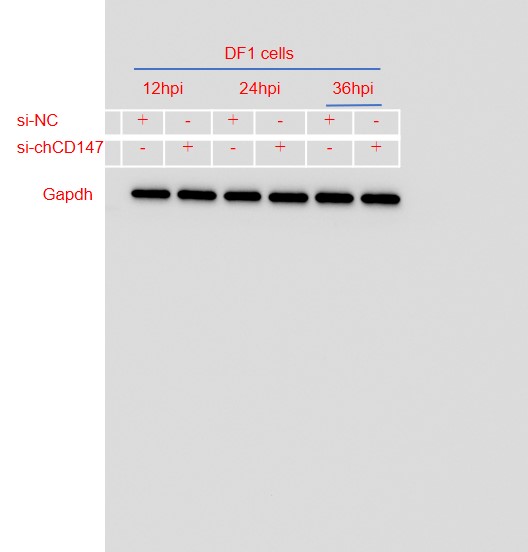

Supplement: Supplementary file 7 [file DataSheet1.zip › raw data-1/Figure 2/Figure 2F/Gapdh.jpg]

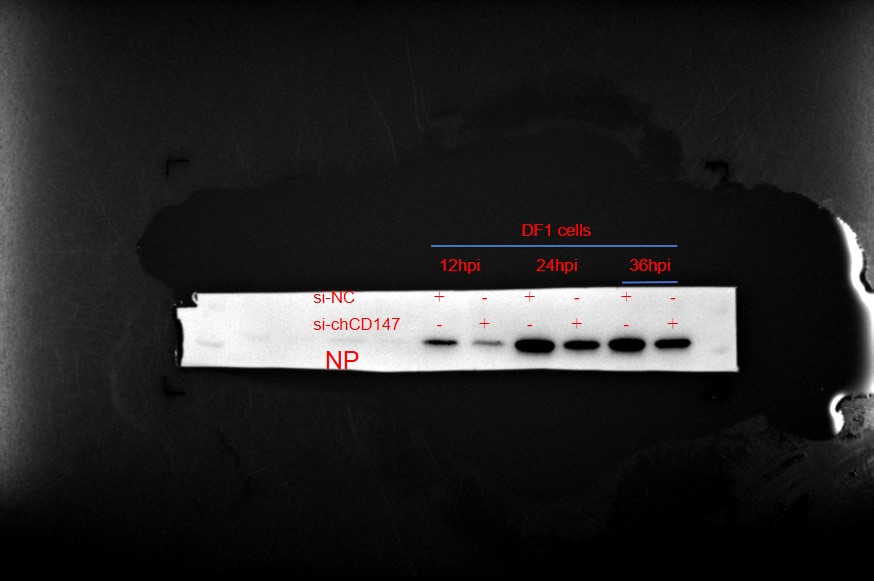

Supplement: Supplementary file 7 [file DataSheet1.zip › raw data-1/Figure 2/Figure 2F/NP.jpg]

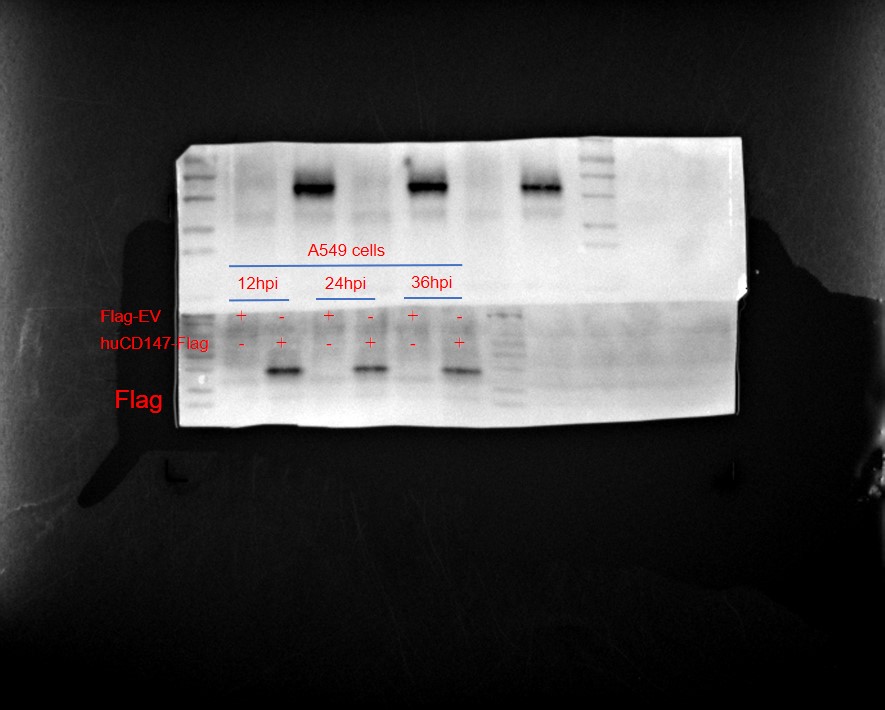

Supplement: Supplementary file 7 [file DataSheet1.zip › raw data-1/Figure 2/Figure 2I/Flag.jpg]

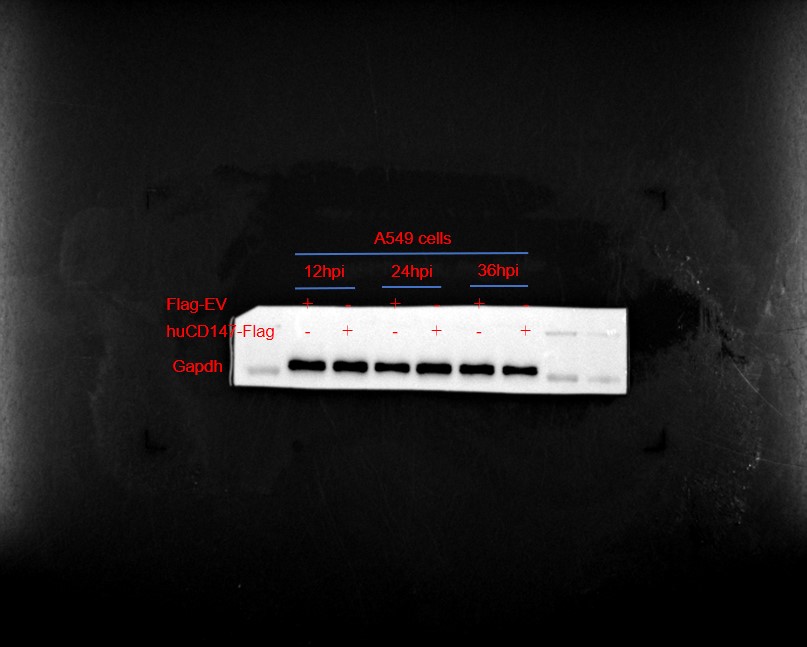

Supplement: Supplementary file 7 [file DataSheet1.zip › raw data-1/Figure 2/Figure 2I/Gapdh.jpg]

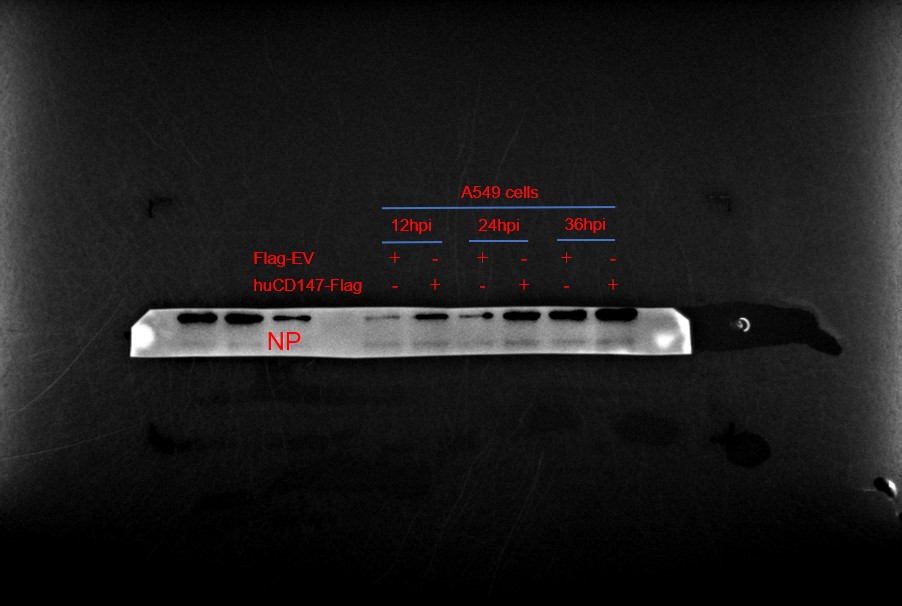

Supplement: Supplementary file 7 [file DataSheet1.zip › raw data-1/Figure 2/Figure 2I/NP.jpg]

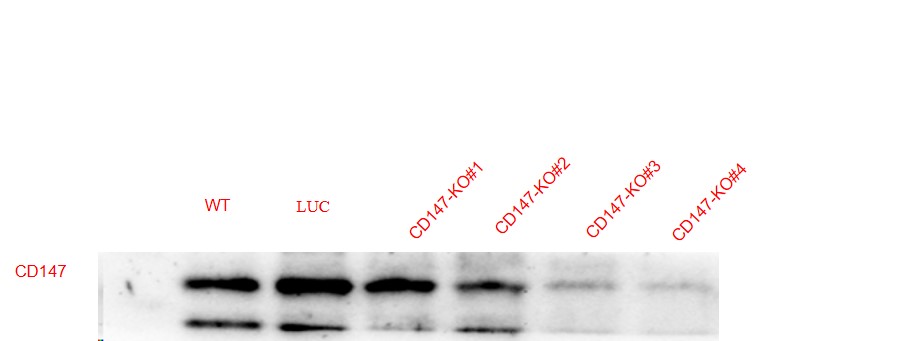

Supplement: Supplementary file 7 [file DataSheet1.zip › raw data-1/Figure 2/Figure 2J/CD147.jpg]

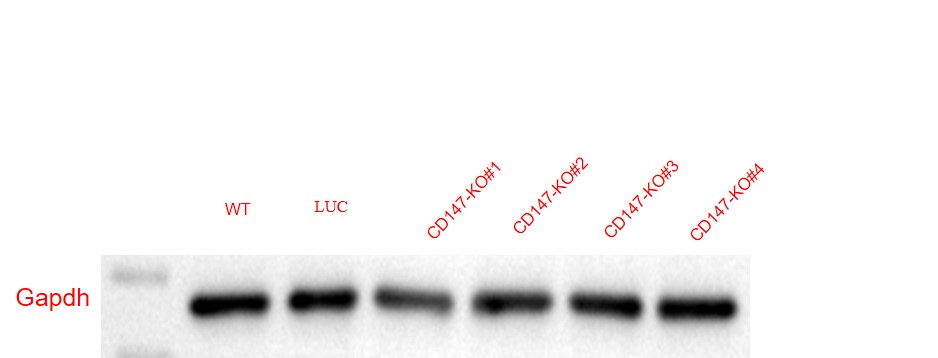

Supplement: Supplementary file 7 [file DataSheet1.zip › raw data-1/Figure 2/Figure 2J/Gapdh.jpg]

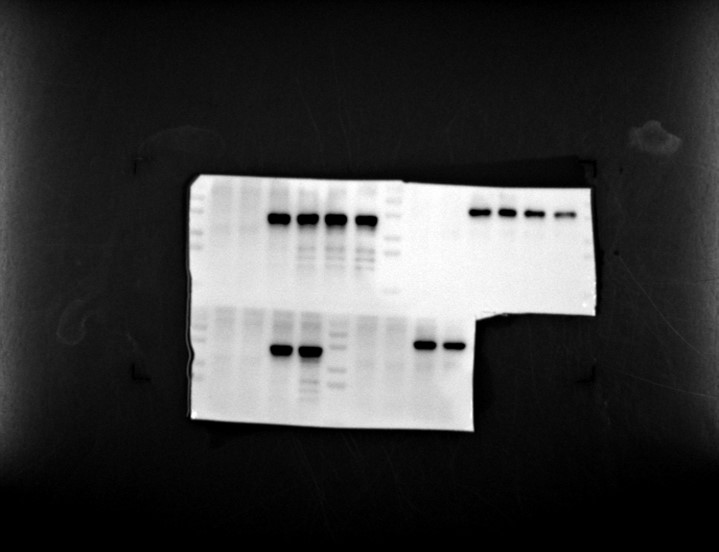

Supplement: Supplementary file 7 [file DataSheet1.zip › raw data-1/Figure 3/3-A chCD147-H5 Co-IP/INPUT Flag.jpg]

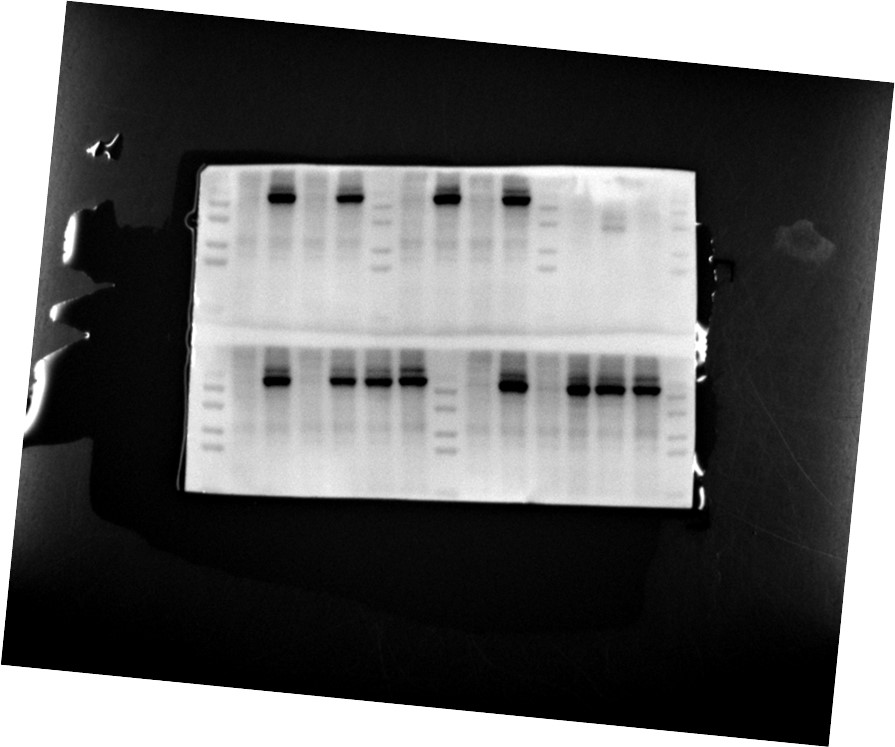

Supplement: Supplementary file 7 [file DataSheet1.zip › raw data-1/Figure 3/3-A chCD147-H5 Co-IP/INPUT ha.jpg]

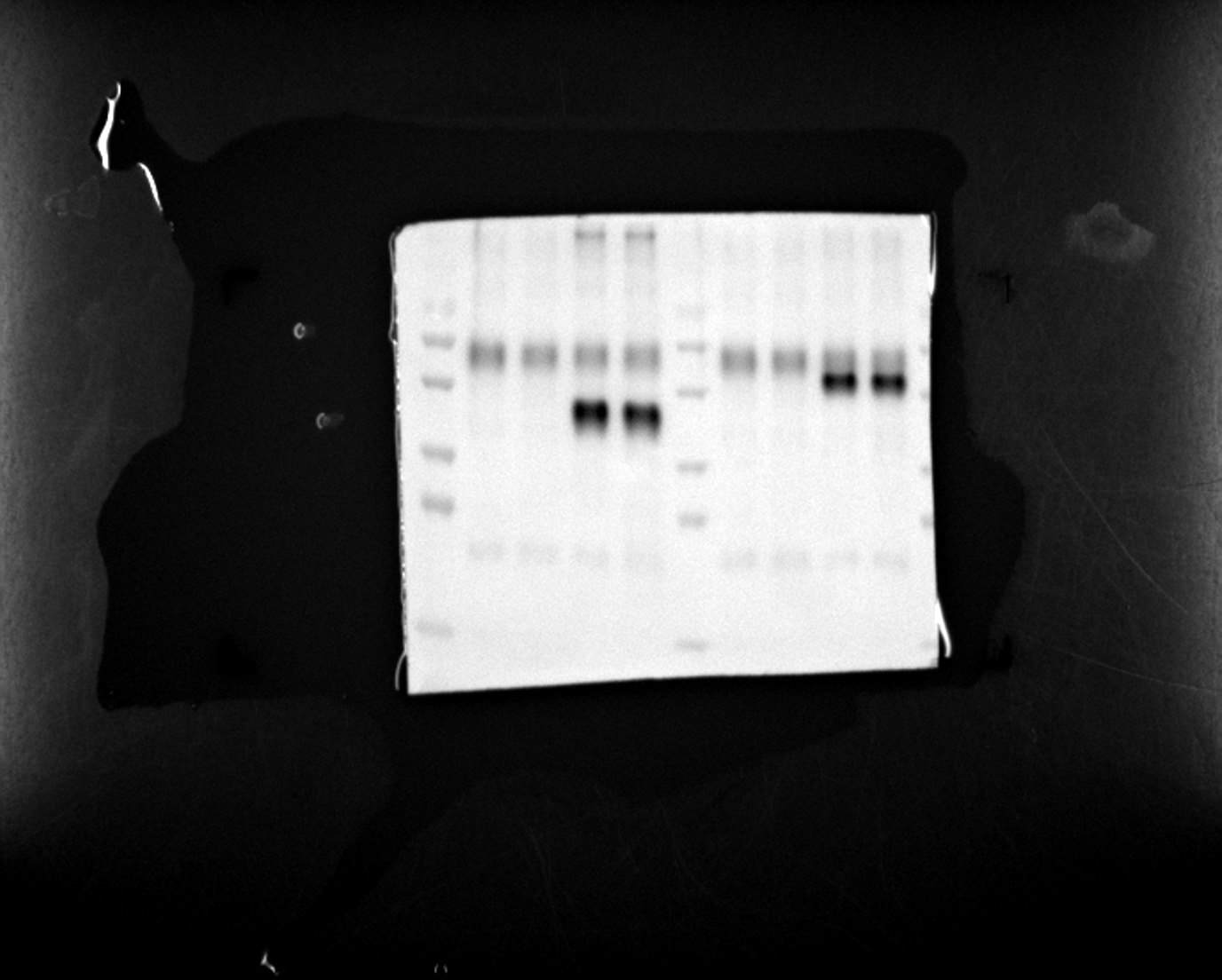

Supplement: Supplementary file 7 [file DataSheet1.zip › raw data-1/Figure 3/3-A chCD147-H5 Co-IP/IP FLAG.jpg]

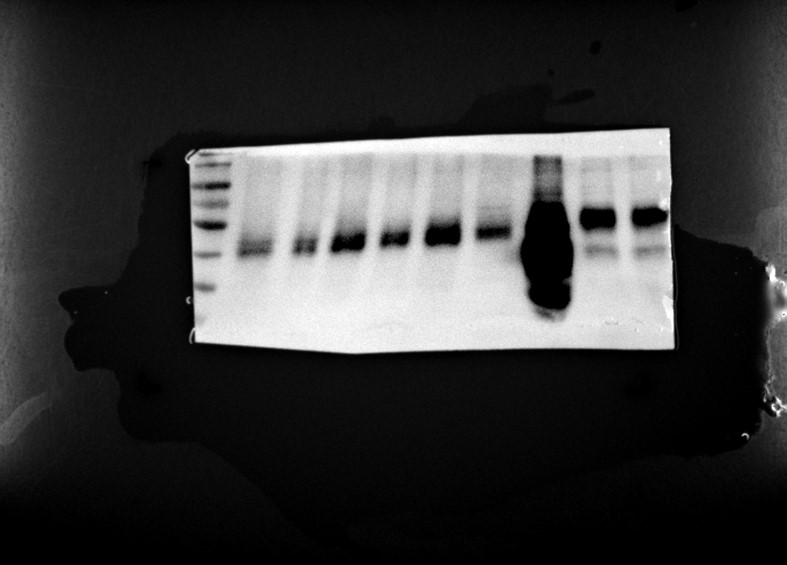

Supplement: Supplementary file 7 [file DataSheet1.zip › raw data-1/Figure 3/3-B DF1 H5内源性/CD147-1.jpg]

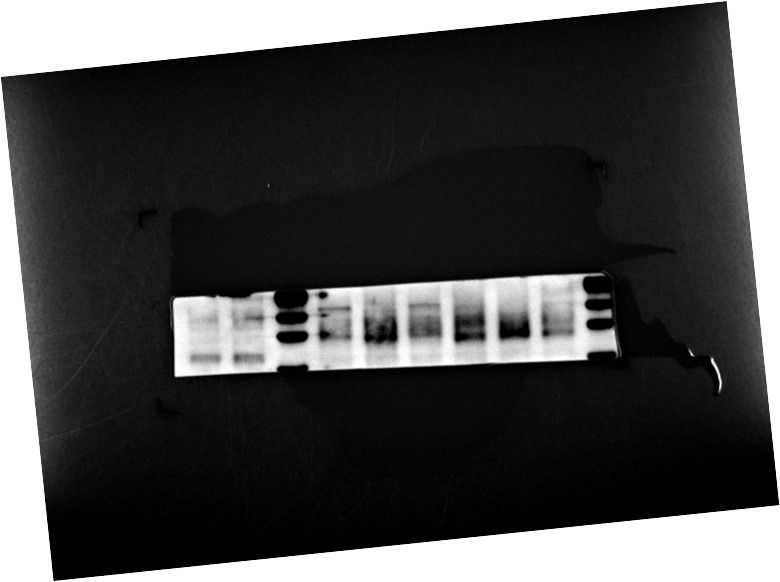

Supplement: Supplementary file 7 [file DataSheet1.zip › raw data-1/Figure 3/3-B DF1 H5内源性/CD147-2.jpg]

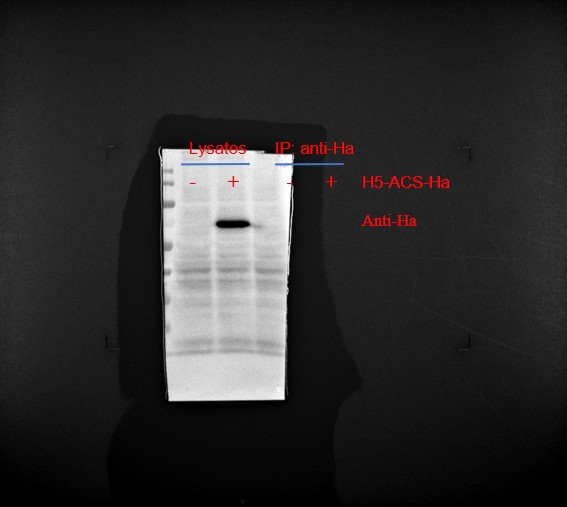

Supplement: Supplementary file 7 [file DataSheet1.zip › raw data-1/Figure 3/3-B DF1 H5内源性/H5-1.jpg]

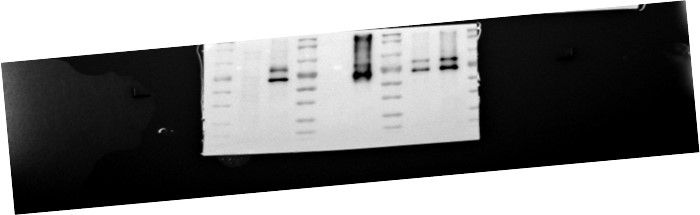

Supplement: Supplementary file 7 [file DataSheet1.zip › raw data-1/Figure 3/3-B DF1 H5内源性/Ha 2.jpg]

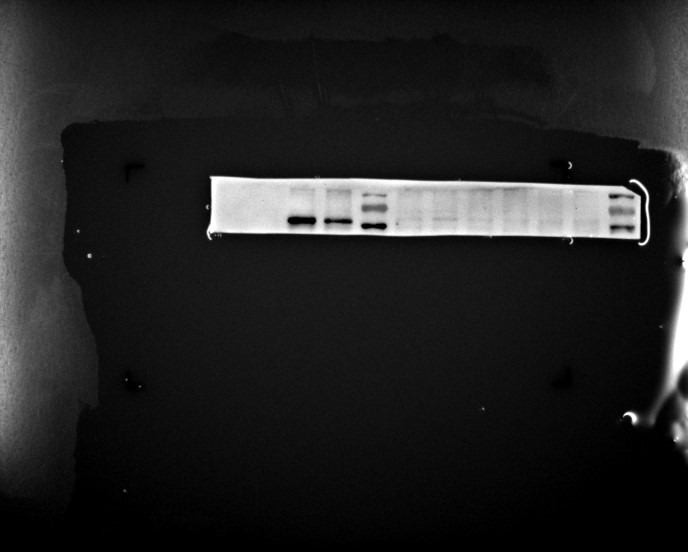

Supplement: Supplementary file 7 [file DataSheet1.zip › raw data-1/Figure 3/3-D huCD147-H1 Co-IP/Input Ha.jpg]

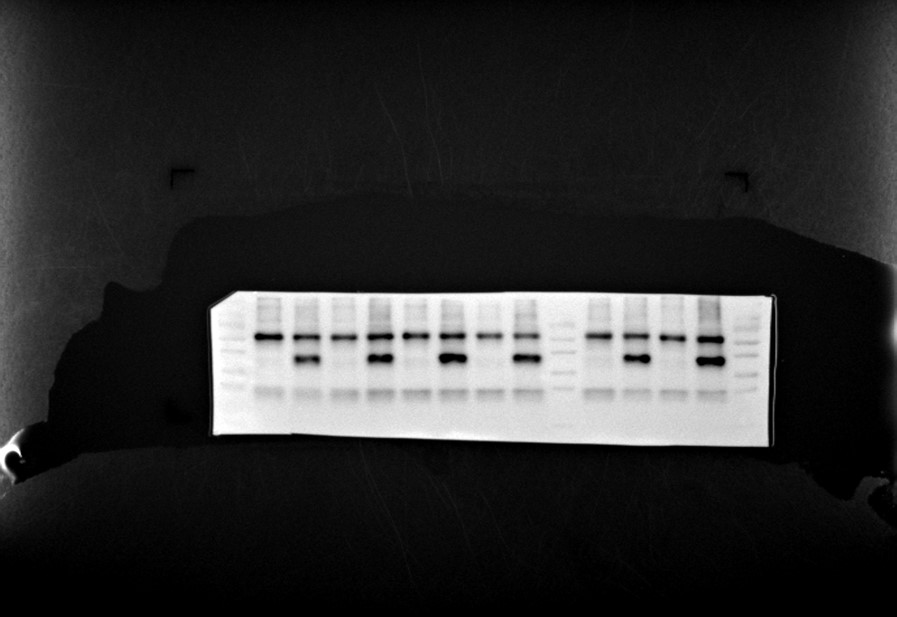

Supplement: Supplementary file 7 [file DataSheet1.zip › raw data-1/Figure 3/3-D huCD147-H1 Co-IP/IP Flag.jpg]

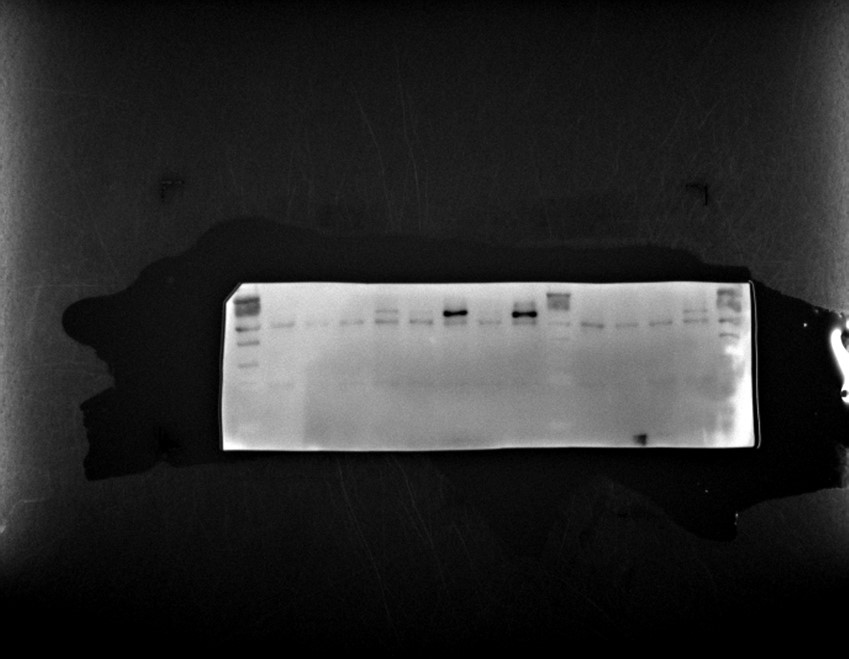

Supplement: Supplementary file 7 [file DataSheet1.zip › raw data-1/Figure 3/3-D huCD147-H1 Co-IP/IP Ha.jpg]

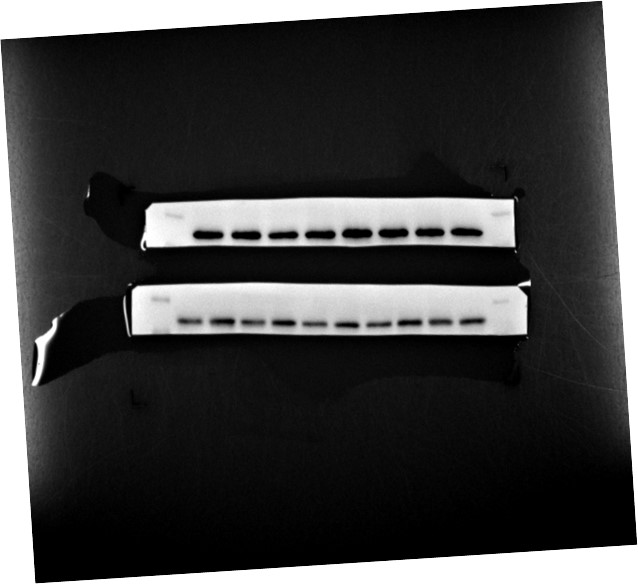

Supplement: Supplementary file 7 [file DataSheet1.zip › raw data-1/Figure 3/3-E HuCD147-H1H5H9 Co-IP/gapdh.jpg]

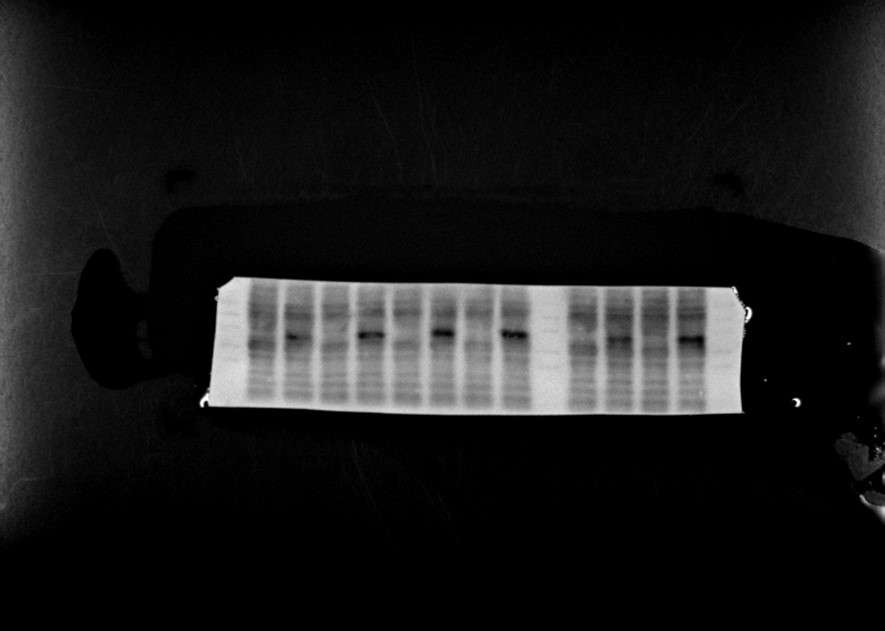

Supplement: Supplementary file 7 [file DataSheet1.zip › raw data-1/Figure 3/3-E HuCD147-H1H5H9 Co-IP/Input FLAG.jpg]

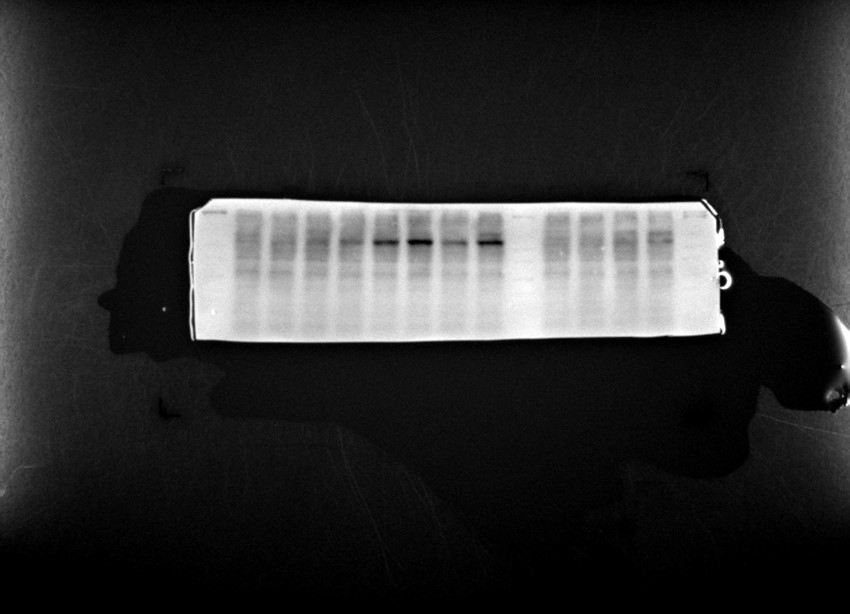

Supplement: Supplementary file 7 [file DataSheet1.zip › raw data-1/Figure 3/3-E HuCD147-H1H5H9 Co-IP/Input Ha.jpg]

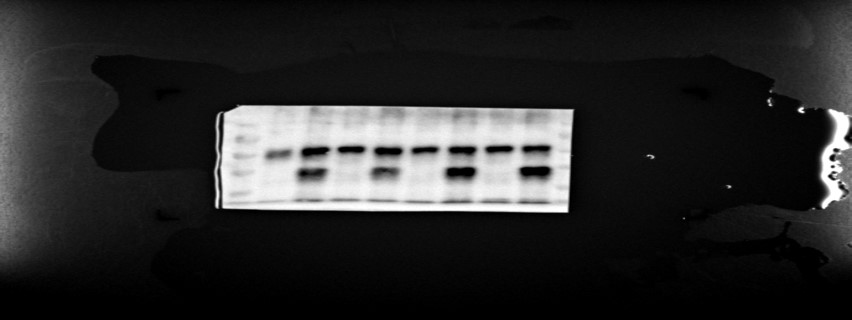

Supplement: Supplementary file 7 [file DataSheet1.zip › raw data-1/Figure 3/3-E HuCD147-H1H5H9 Co-IP/IP Flag.jpg]

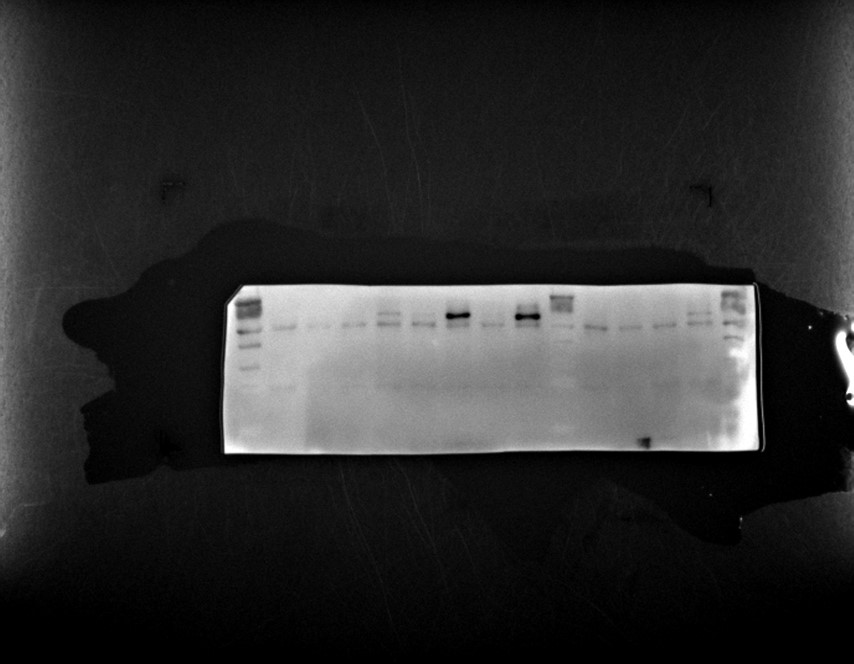

Supplement: Supplementary file 7 [file DataSheet1.zip › raw data-1/Figure 3/3-E HuCD147-H1H5H9 Co-IP/IP Ha.jpg]

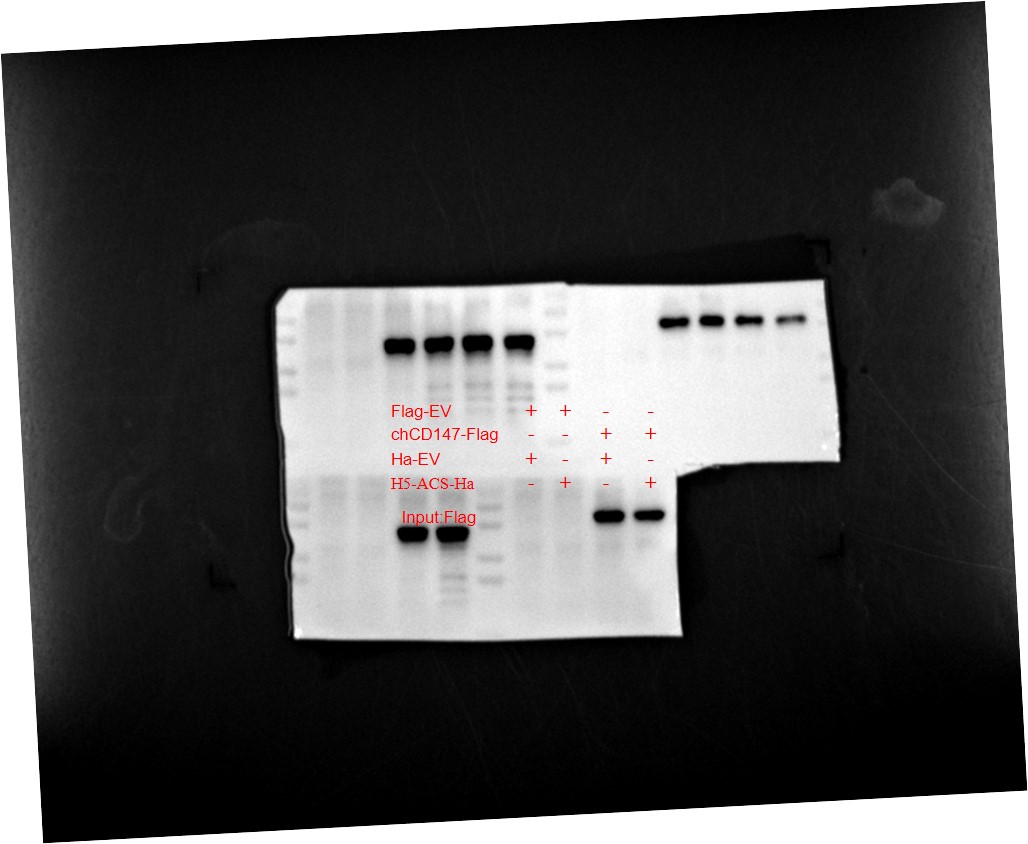

Supplement: Supplementary file 7 [file DataSheet1.zip › raw data-1/Figure 3/Figure3 A/INPUT Flag.jpg]

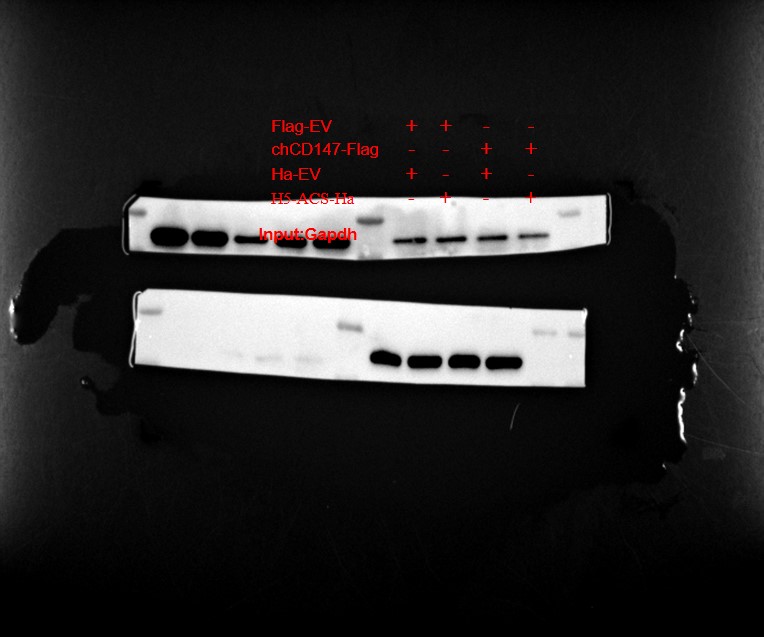

Supplement: Supplementary file 7 [file DataSheet1.zip › raw data-1/Figure 3/Figure3 A/INPUT Gapdh.jpg]

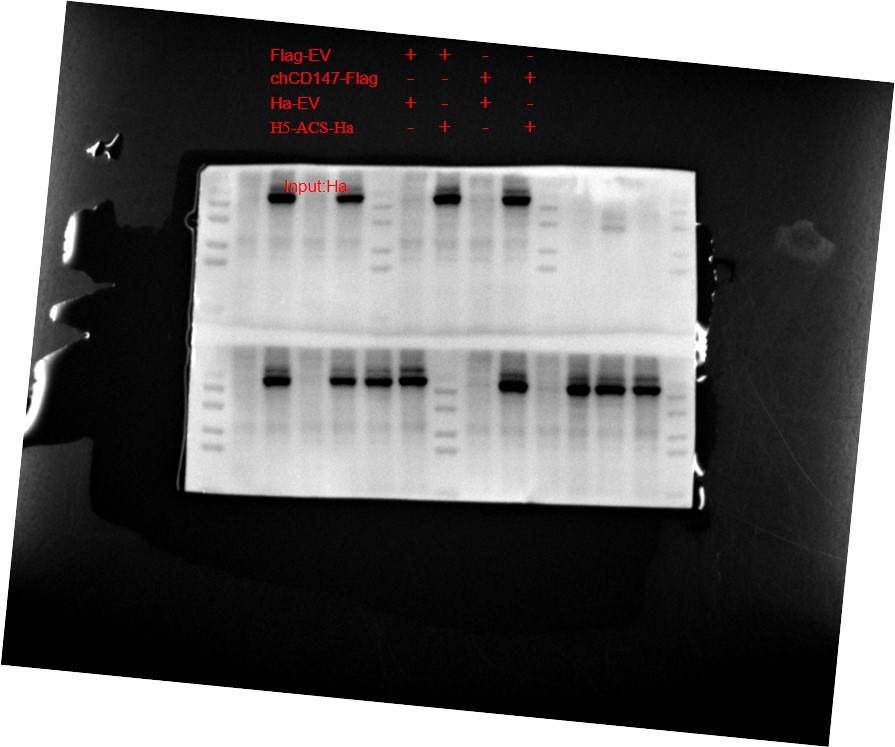

Supplement: Supplementary file 7 [file DataSheet1.zip › raw data-1/Figure 3/Figure3 A/INPUT Ha.jpg]

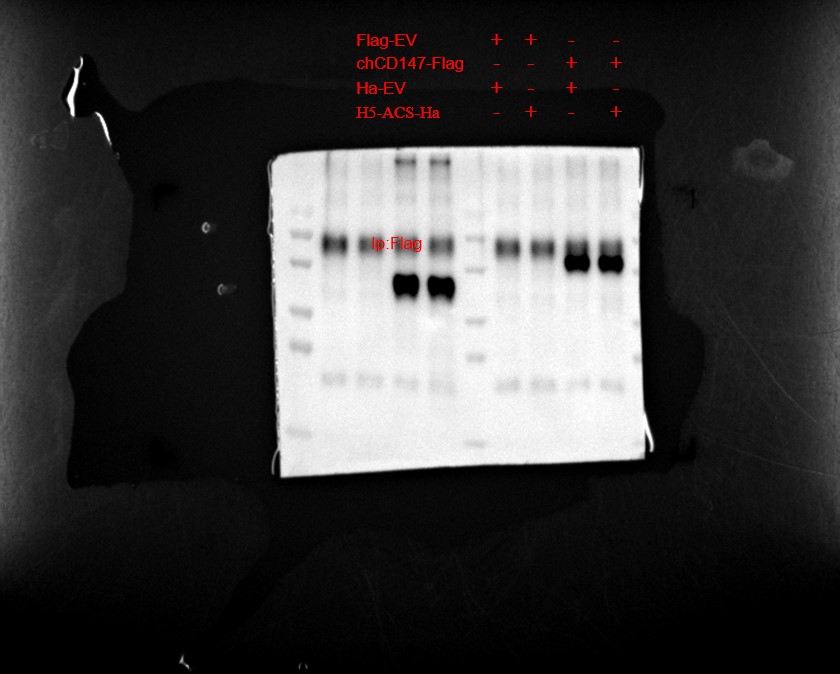

Supplement: Supplementary file 7 [file DataSheet1.zip › raw data-1/Figure 3/Figure3 A/IP Flag.jpg]

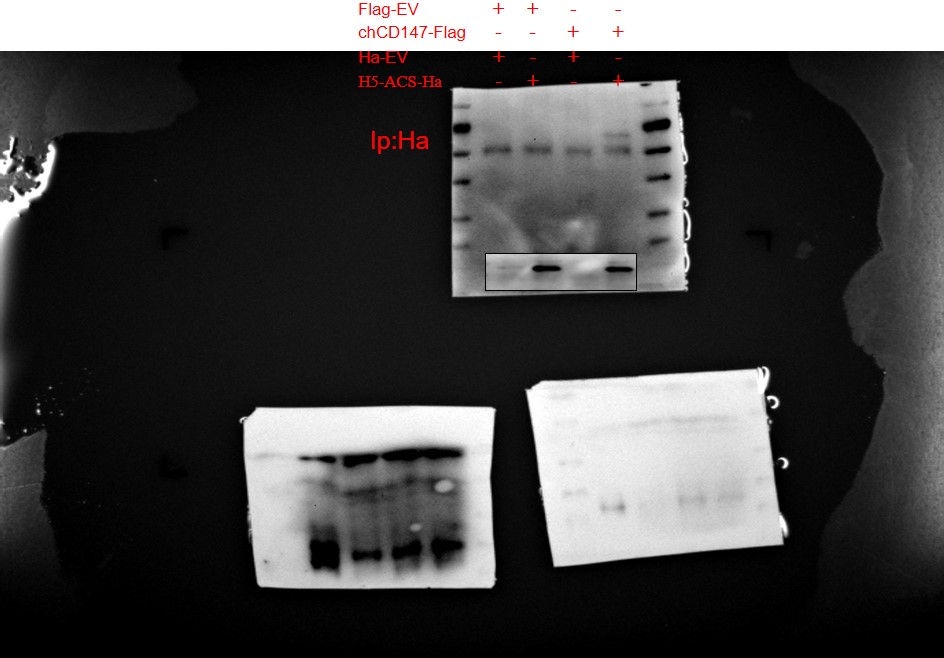

Supplement: Supplementary file 7 [file DataSheet1.zip › raw data-1/Figure 3/Figure3 A/IP Ha.jpg]

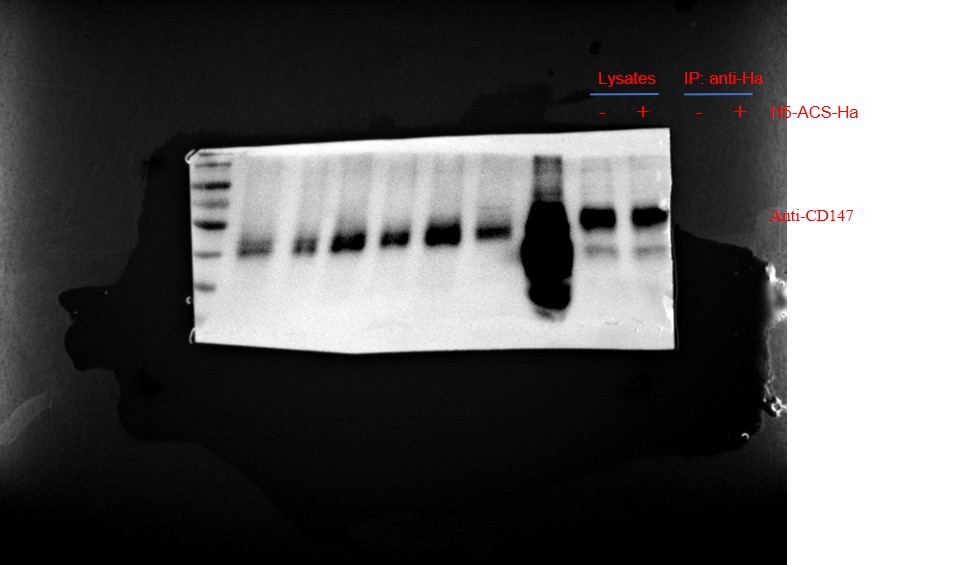

Supplement: Supplementary file 7 [file DataSheet1.zip › raw data-1/Figure 3/Figure3 B/CD147-1.jpg]

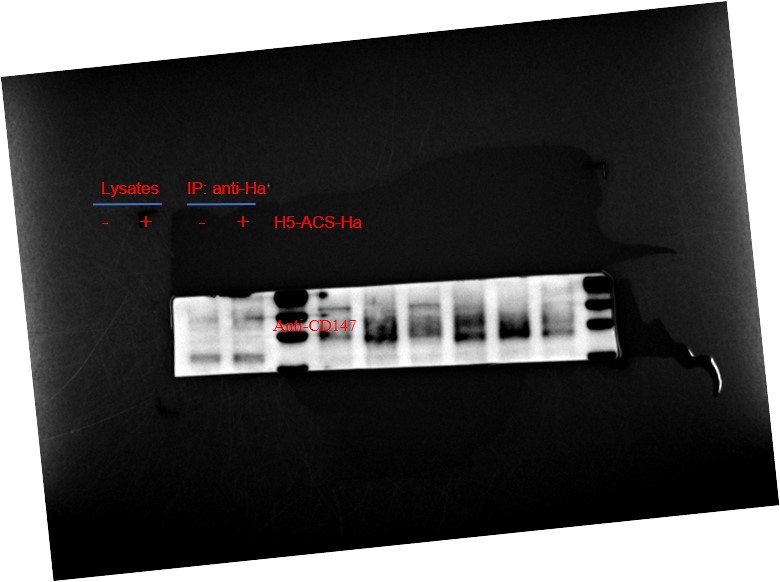

Supplement: Supplementary file 7 [file DataSheet1.zip › raw data-1/Figure 3/Figure3 B/CD147-2.jpg]

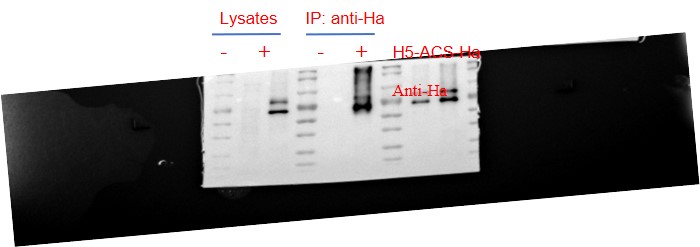

Supplement: Supplementary file 7 [file DataSheet1.zip › raw data-1/Figure 3/Figure3 B/Ha 2.jpg]

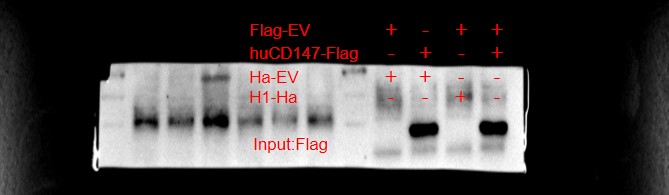

Supplement: Supplementary file 7 [file DataSheet1.zip › raw data-1/Figure 3/Figure3 D/INPUT Flag.jpg]

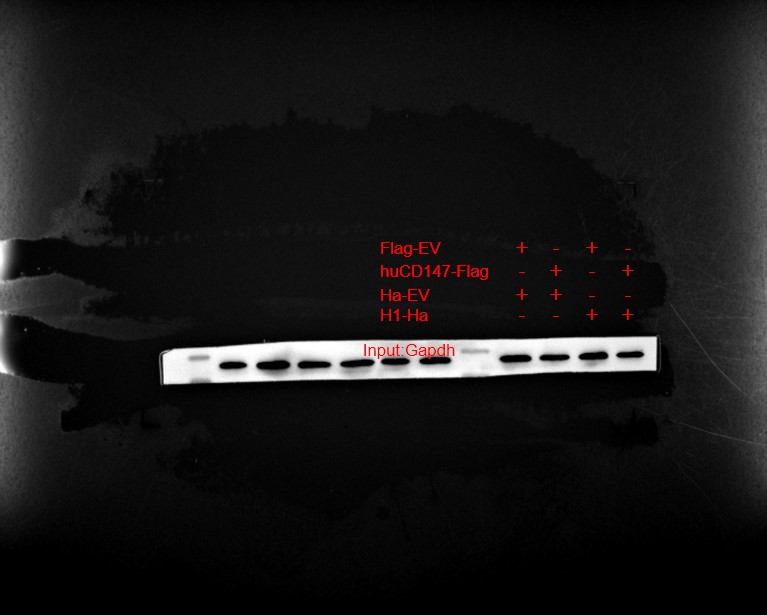

Supplement: Supplementary file 7 [file DataSheet1.zip › raw data-1/Figure 3/Figure3 D/Input Gapdh.jpg]

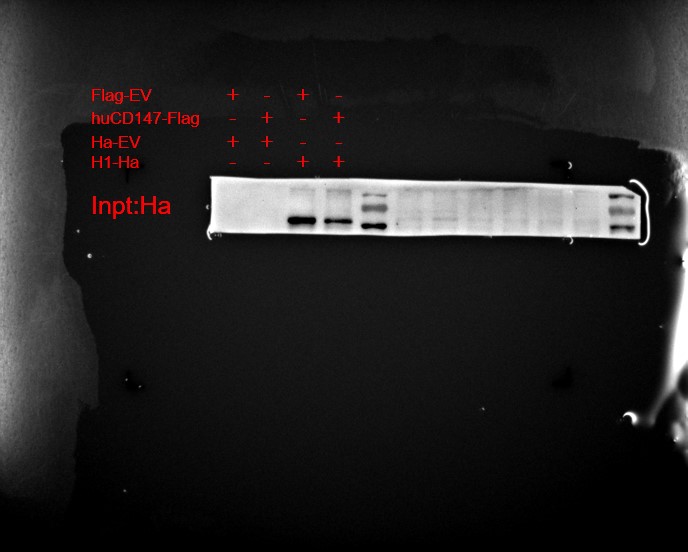

Supplement: Supplementary file 7 [file DataSheet1.zip › raw data-1/Figure 3/Figure3 D/Input Ha.jpg]

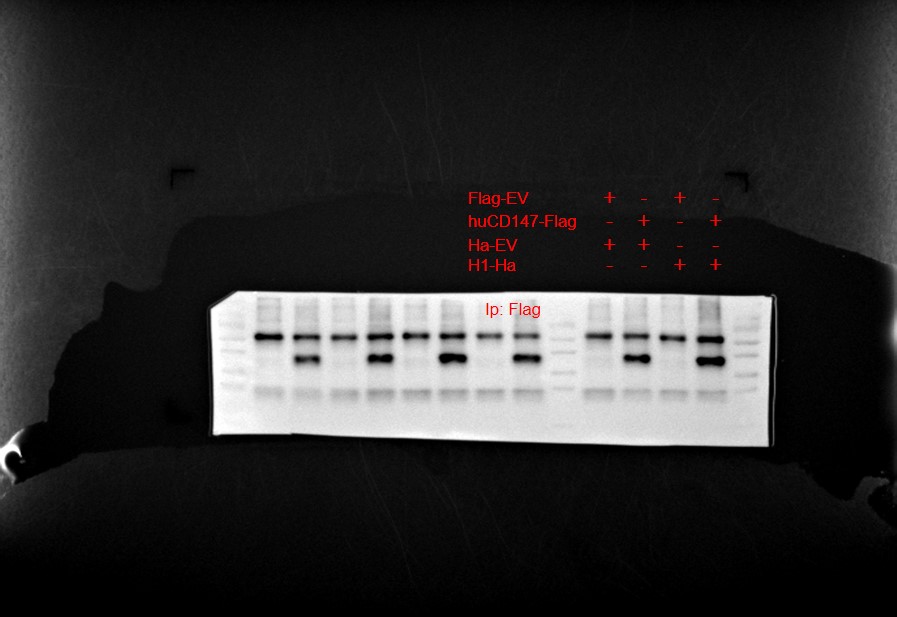

Supplement: Supplementary file 7 [file DataSheet1.zip › raw data-1/Figure 3/Figure3 D/IP Flag.jpg]

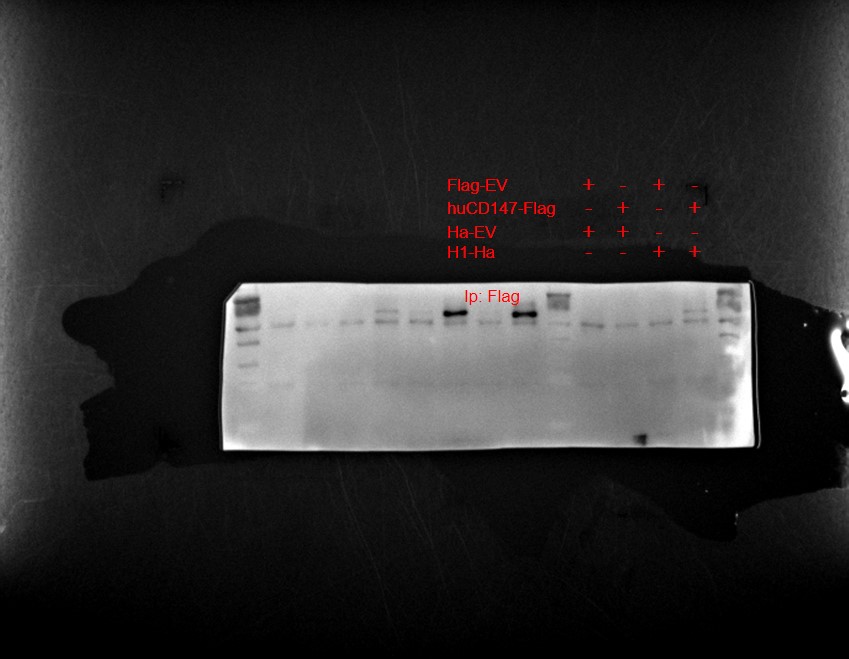

Supplement: Supplementary file 7 [file DataSheet1.zip › raw data-1/Figure 3/Figure3 D/IP ha2.jpg]

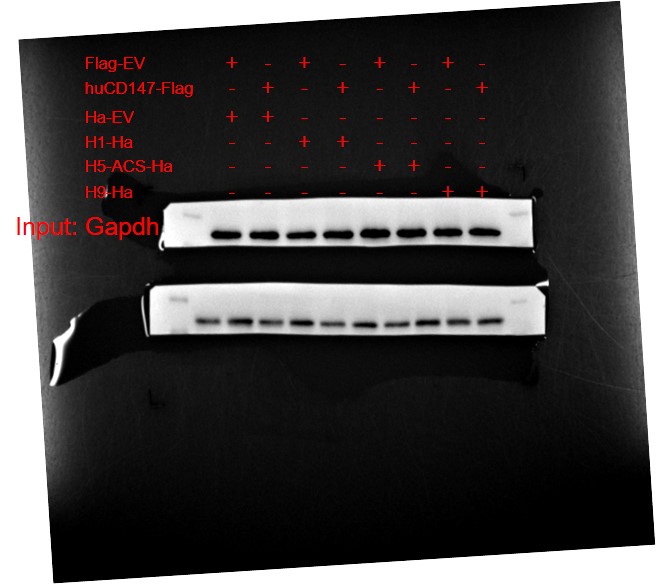

Supplement: Supplementary file 7 [file DataSheet1.zip › raw data-1/Figure 3/Figure3 E/Gapdh.jpg]

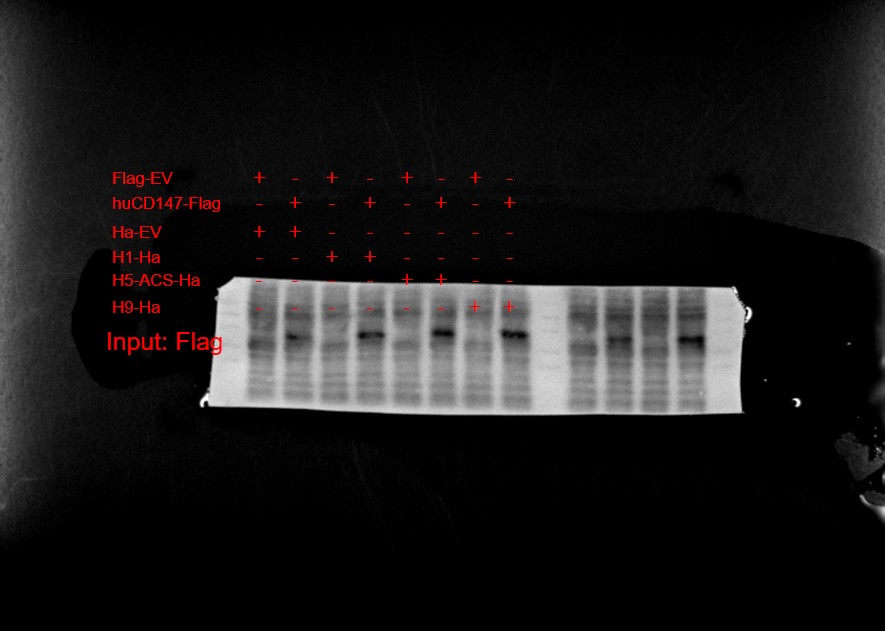

Supplement: Supplementary file 7 [file DataSheet1.zip › raw data-1/Figure 3/Figure3 E/Input Flag.jpg]

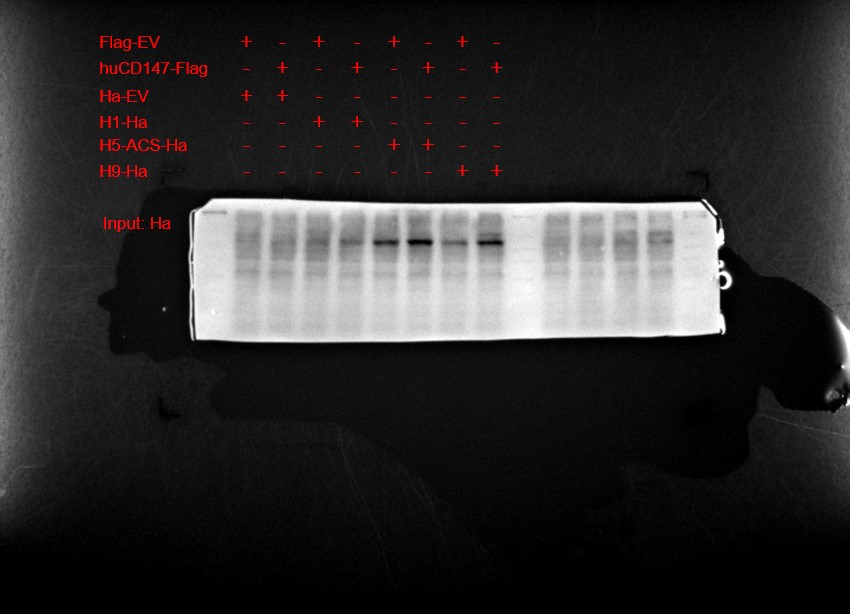

Supplement: Supplementary file 7 [file DataSheet1.zip › raw data-1/Figure 3/Figure3 E/Input Ha.jpg]

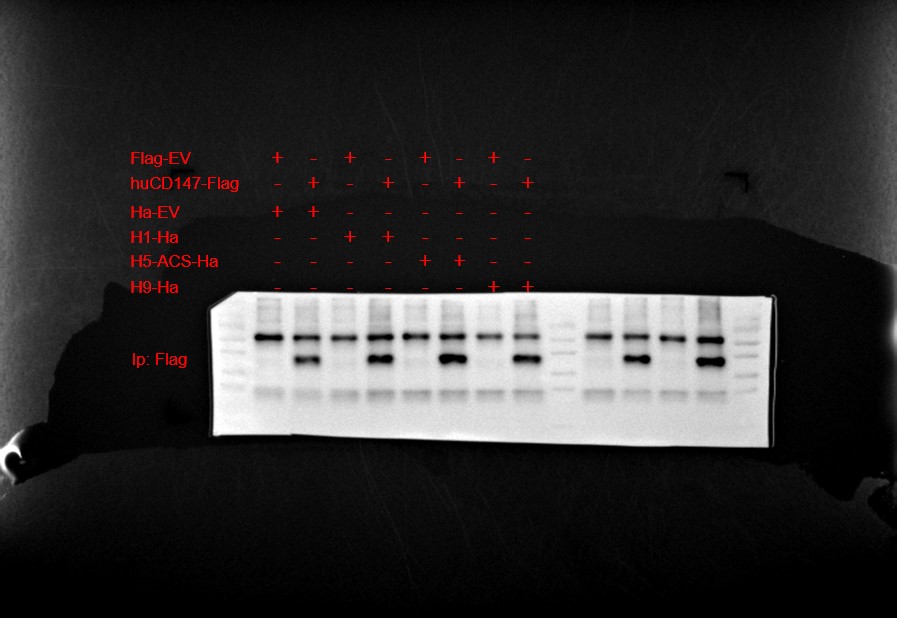

Supplement: Supplementary file 7 [file DataSheet1.zip › raw data-1/Figure 3/Figure3 E/IP Flag.jpg]

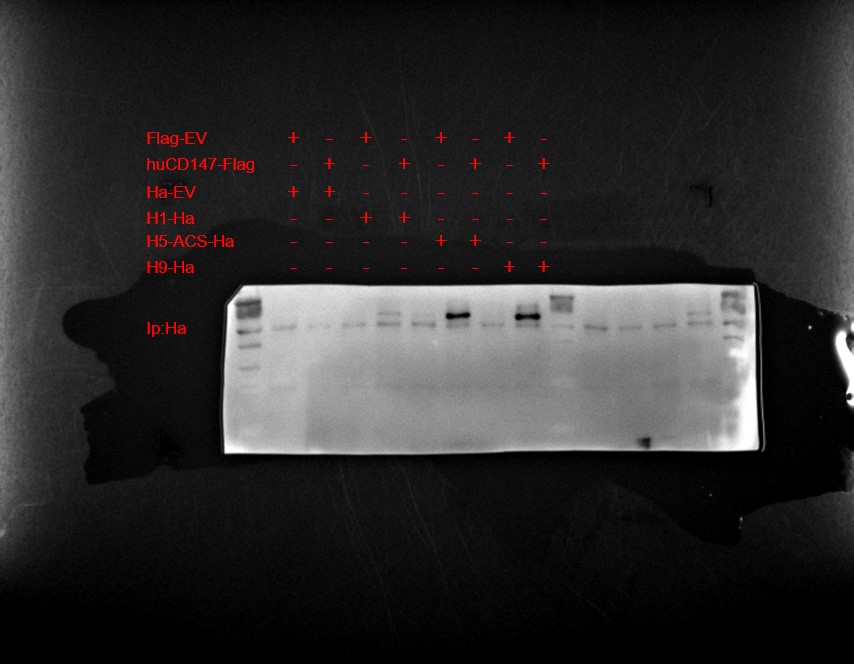

Supplement: Supplementary file 7 [file DataSheet1.zip › raw data-1/Figure 3/Figure3 E/IP Ha.jpg]

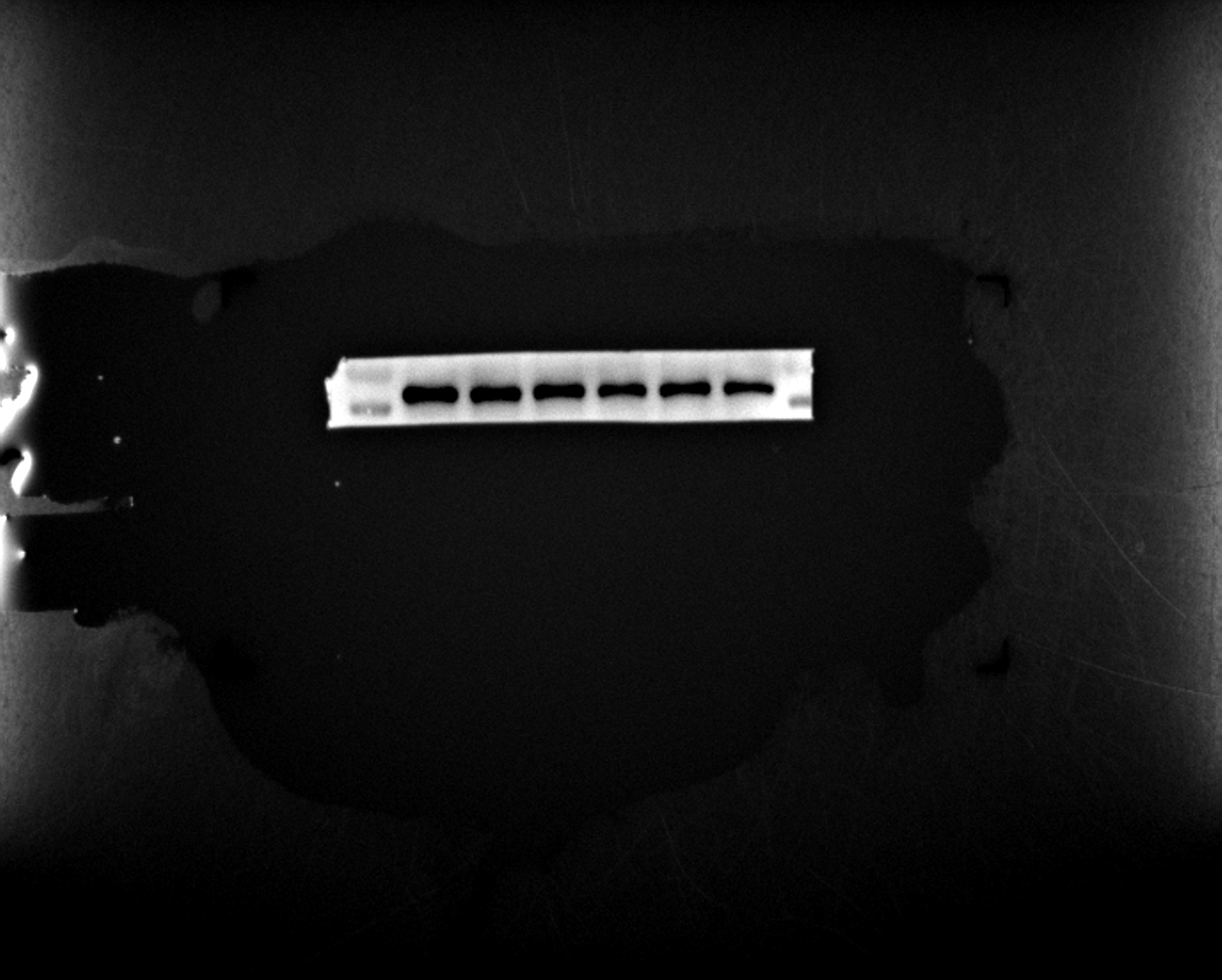

Supplement: Supplementary file 7 [file DataSheet1.zip › raw data-1/Figure 4/4-B DF1-CD147-黏附/DF-J3-4粘附-NP.jpg]

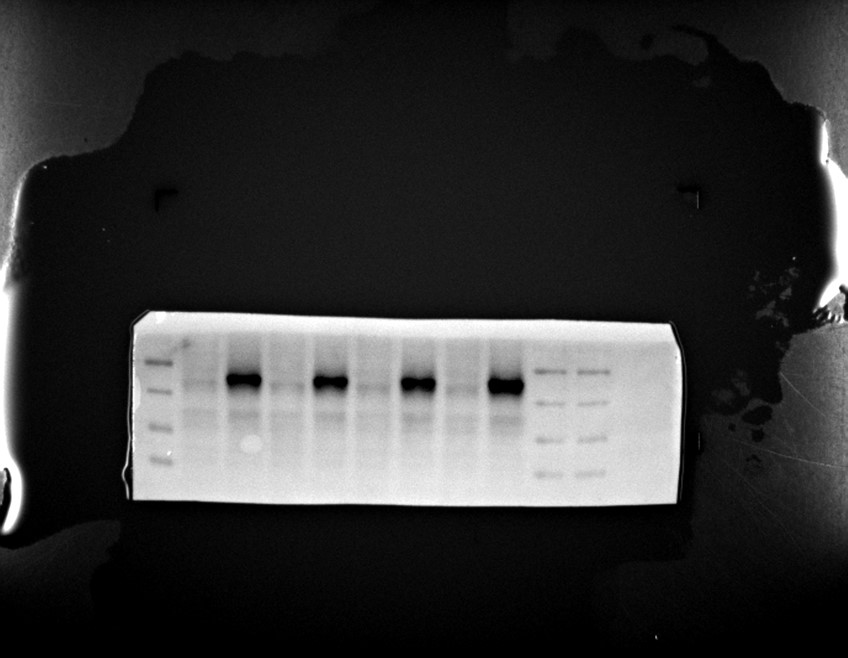

Supplement: Supplementary file 7 [file DataSheet1.zip › raw data-1/Figure 4/4-B DF1-CD147-黏附/Flag.jpg]

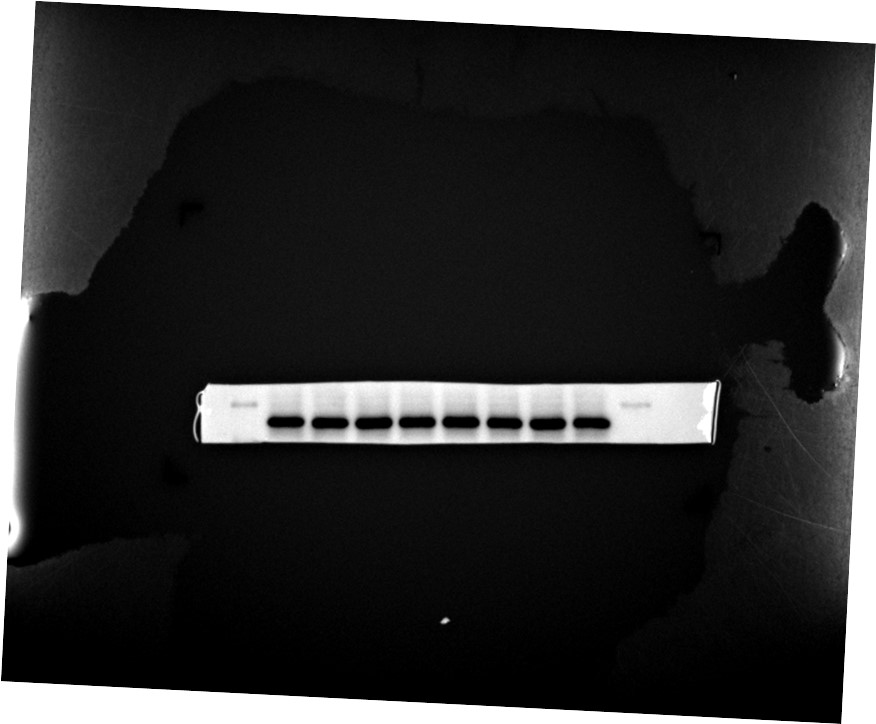

Supplement: Supplementary file 7 [file DataSheet1.zip › raw data-1/Figure 4/4-B DF1-CD147-黏附/GAPDH.jpg]

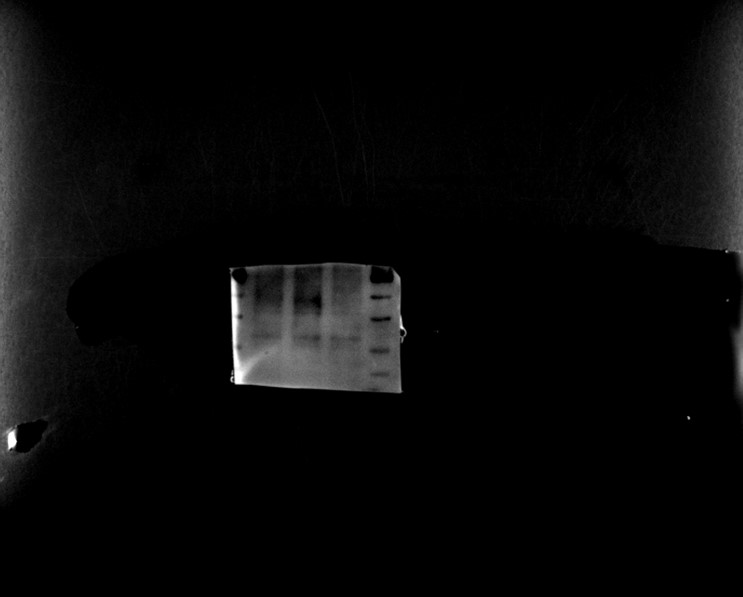

Supplement: Supplementary file 7 [file DataSheet1.zip › raw data-1/Figure 4/4-D DF1-SiCD147 H9N2黏附/CD147.jpg]

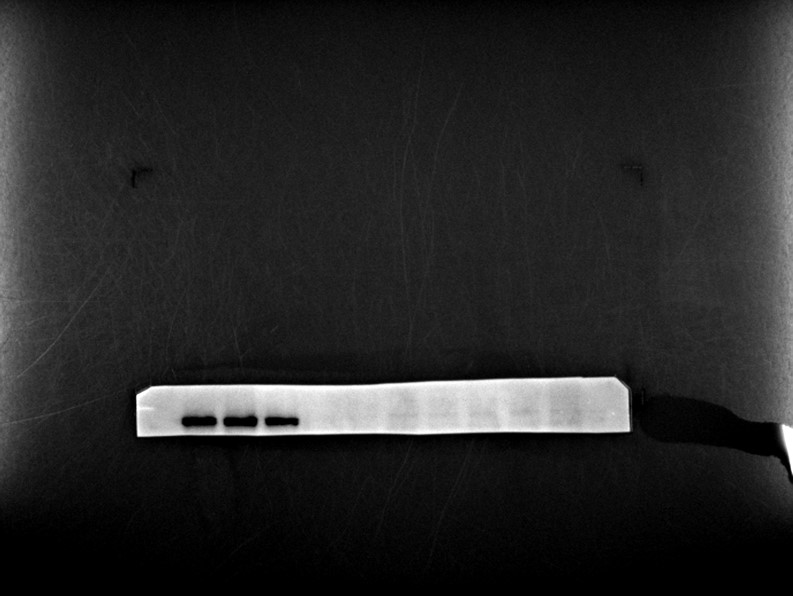

Supplement: Supplementary file 7 [file DataSheet1.zip › raw data-1/Figure 4/4-D DF1-SiCD147 H9N2黏附/Gapdh 1.jpg]

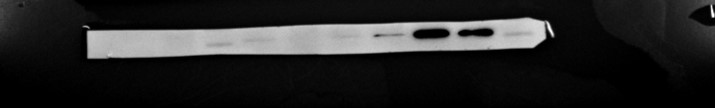

Supplement: Supplementary file 7 [file DataSheet1.zip › raw data-1/Figure 4/4-D DF1-SiCD147 H9N2黏附/Gapdh 2.jpg]

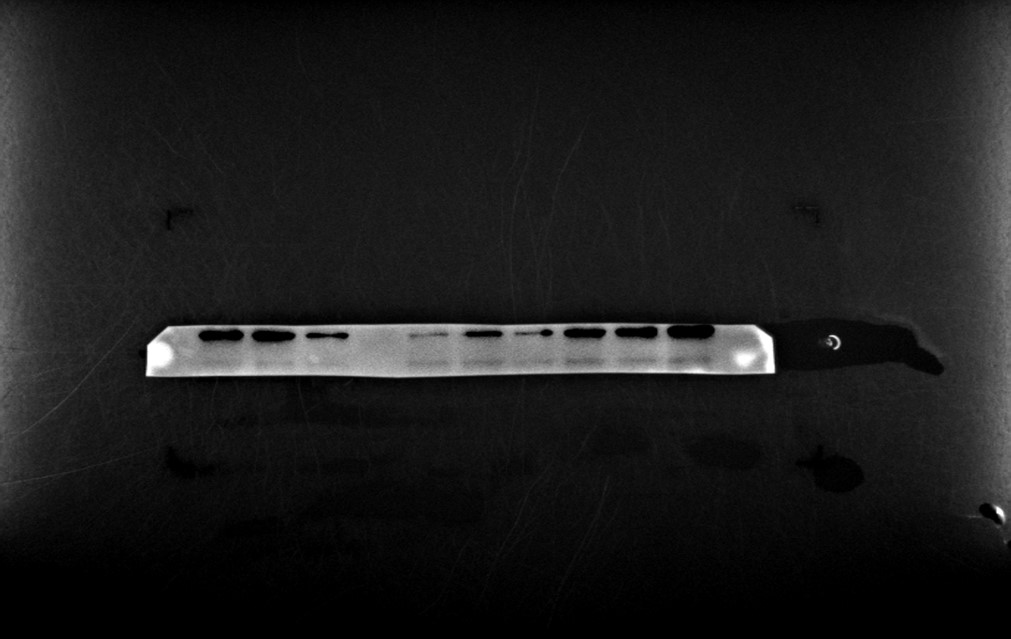

Supplement: Supplementary file 7 [file DataSheet1.zip › raw data-1/Figure 4/4-D DF1-SiCD147 H9N2黏附/NP-1.jpg]

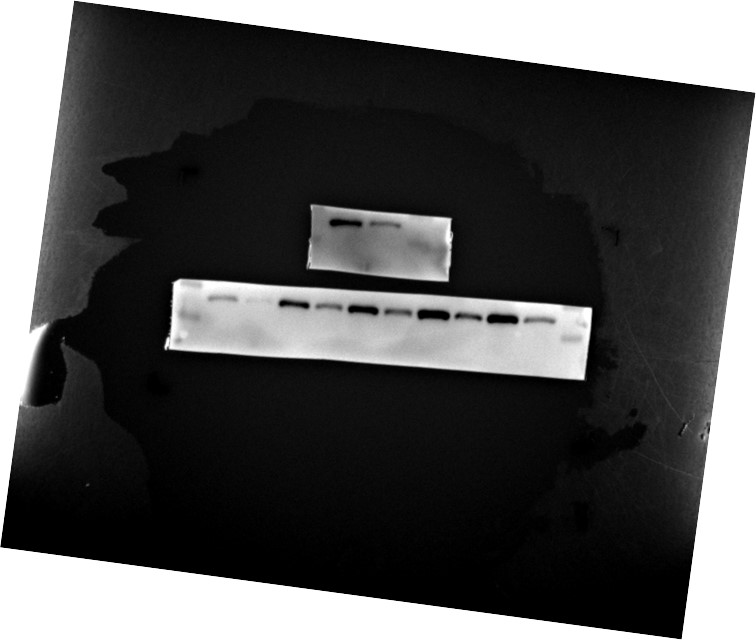

Supplement: Supplementary file 7 [file DataSheet1.zip › raw data-1/Figure 4/4-D DF1-SiCD147 H9N2黏附/NP-2.jpg]

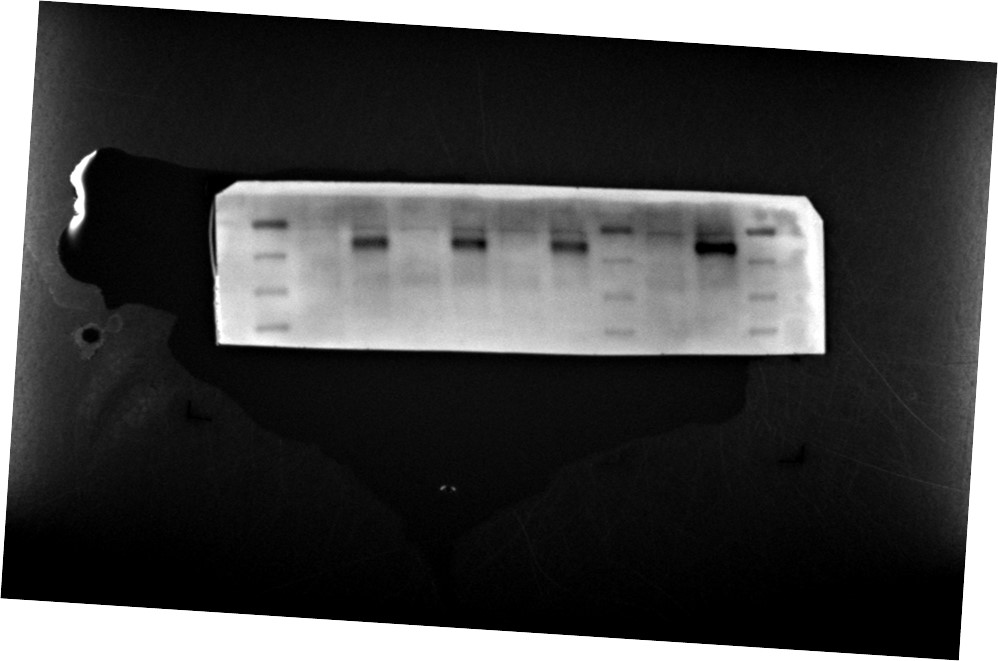

Supplement: Supplementary file 7 [file DataSheet1.zip › raw data-1/Figure 4/4-F DF1-siCD147-JX/flag-1.jpg]

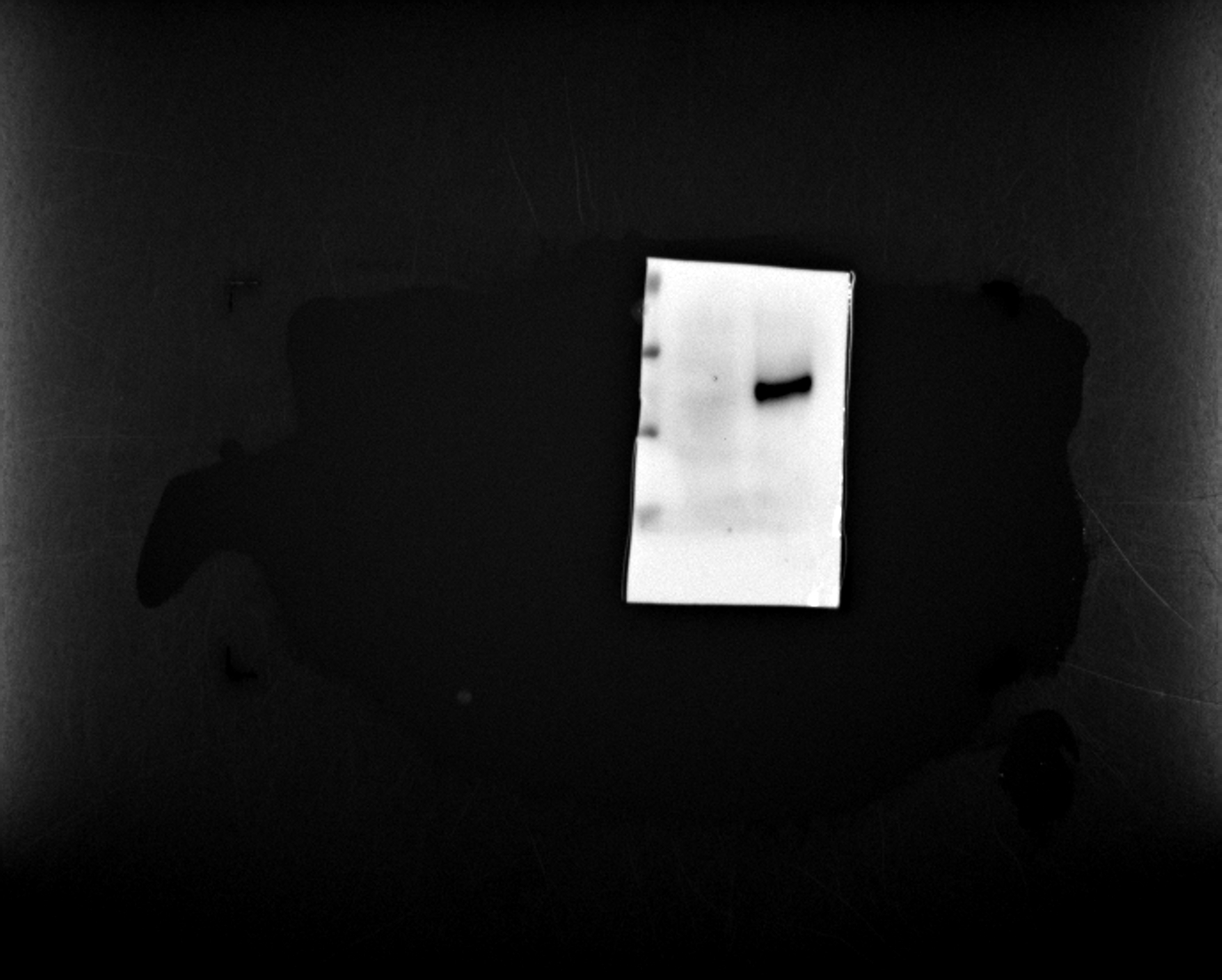

Supplement: Supplementary file 7 [file DataSheet1.zip › raw data-1/Figure 4/4-F DF1-siCD147-JX/flag-2.jpg]

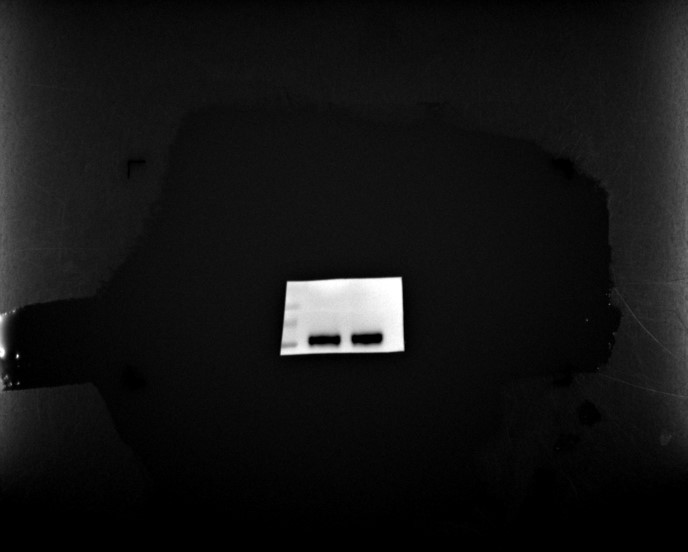

Supplement: Supplementary file 7 [file DataSheet1.zip › raw data-1/Figure 4/4-F DF1-siCD147-JX/gapdh-1.jpg]

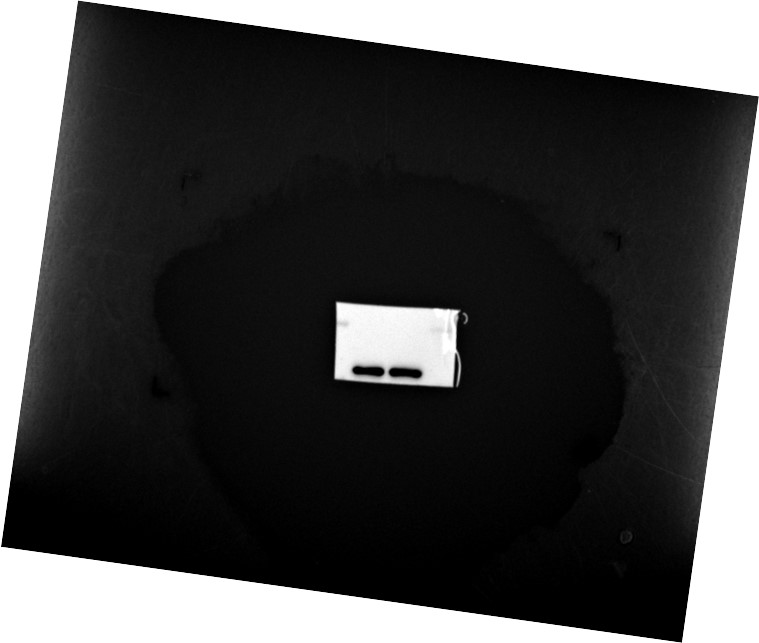

Supplement: Supplementary file 7 [file DataSheet1.zip › raw data-1/Figure 4/4-F DF1-siCD147-JX/gapdh-2.jpg]

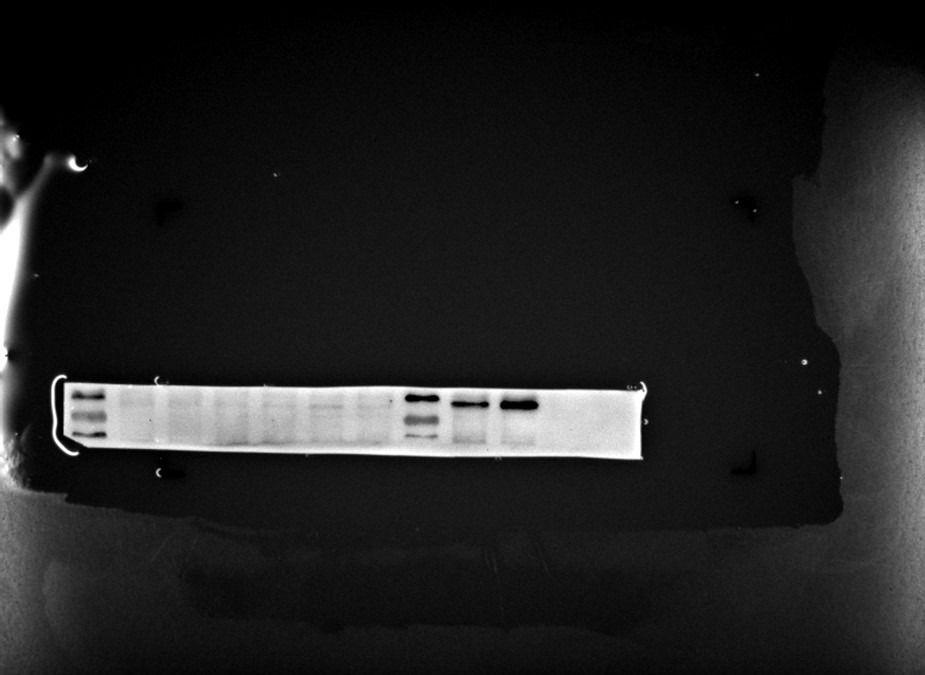

Supplement: Supplementary file 7 [file DataSheet1.zip › raw data-1/Figure 4/4-F DF1-siCD147-JX/NP-1.jpg]

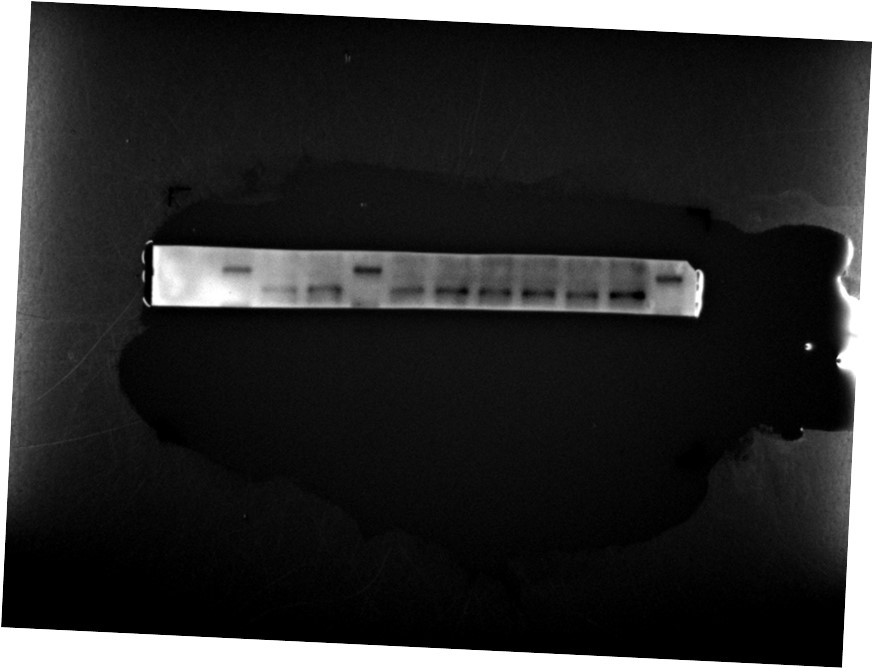

Supplement: Supplementary file 7 [file DataSheet1.zip › raw data-1/Figure 4/4-F DF1-siCD147-JX/NP-2.jpg]

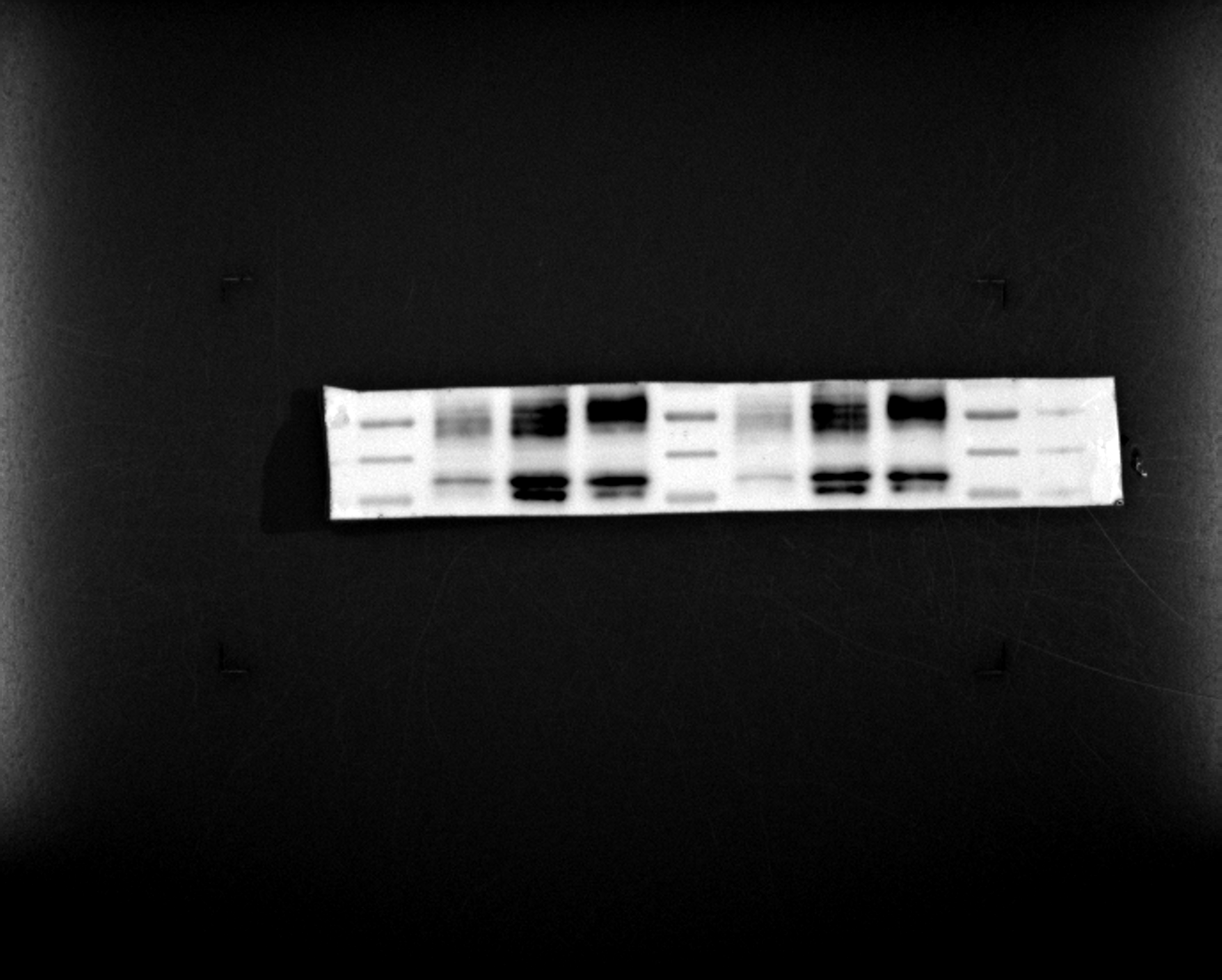

Supplement: Supplementary file 7 [file DataSheet1.zip › raw data-1/Figure 4/4-H A549-KO-CD147-PR8黏附/A549-KO-BG-黏附 入侵-CD147.Tif]

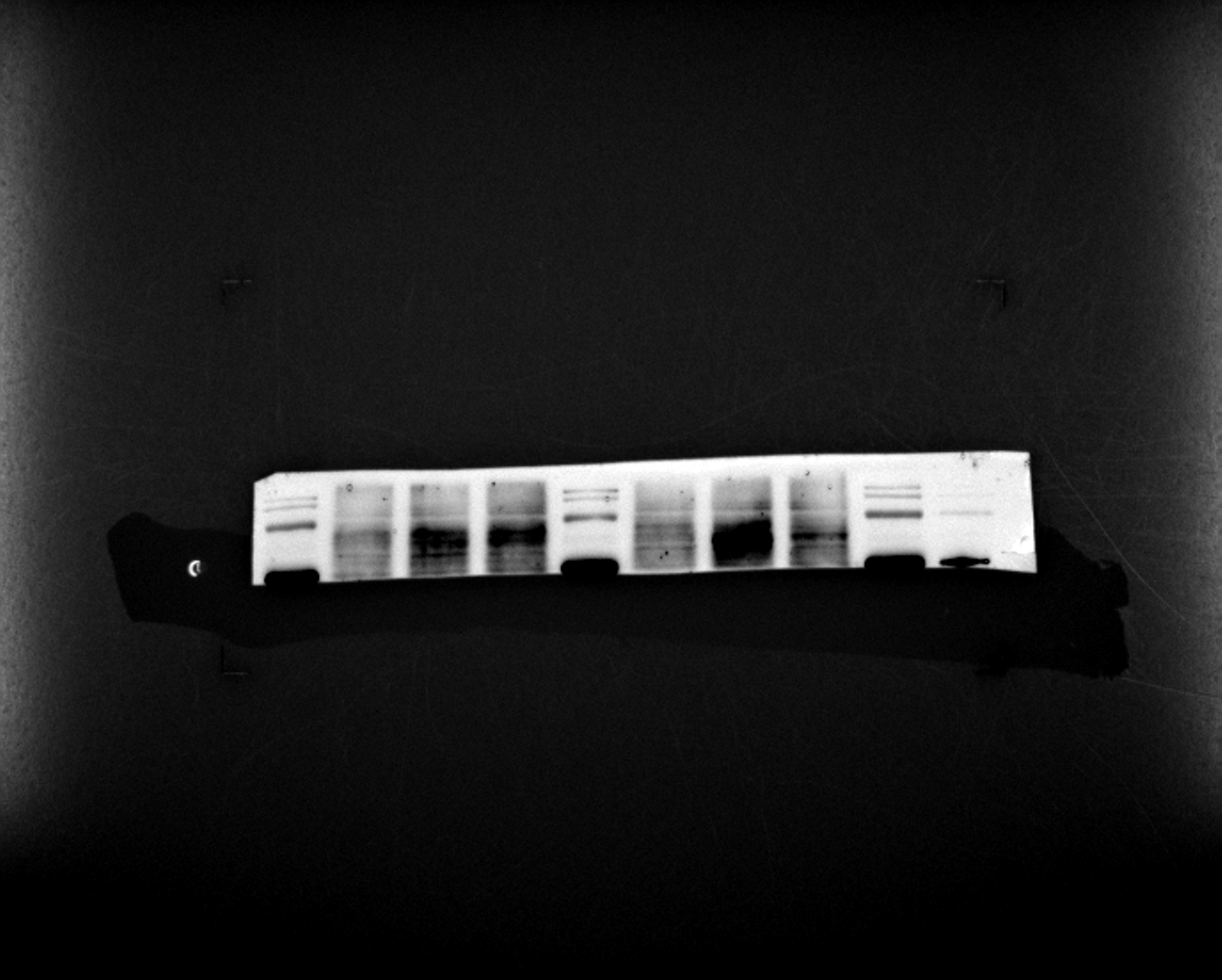

Supplement: Supplementary file 7 [file DataSheet1.zip › raw data-1/Figure 4/4-H A549-KO-CD147-PR8黏附/A549-KO-HA黏附 入侵-NP.Tif]

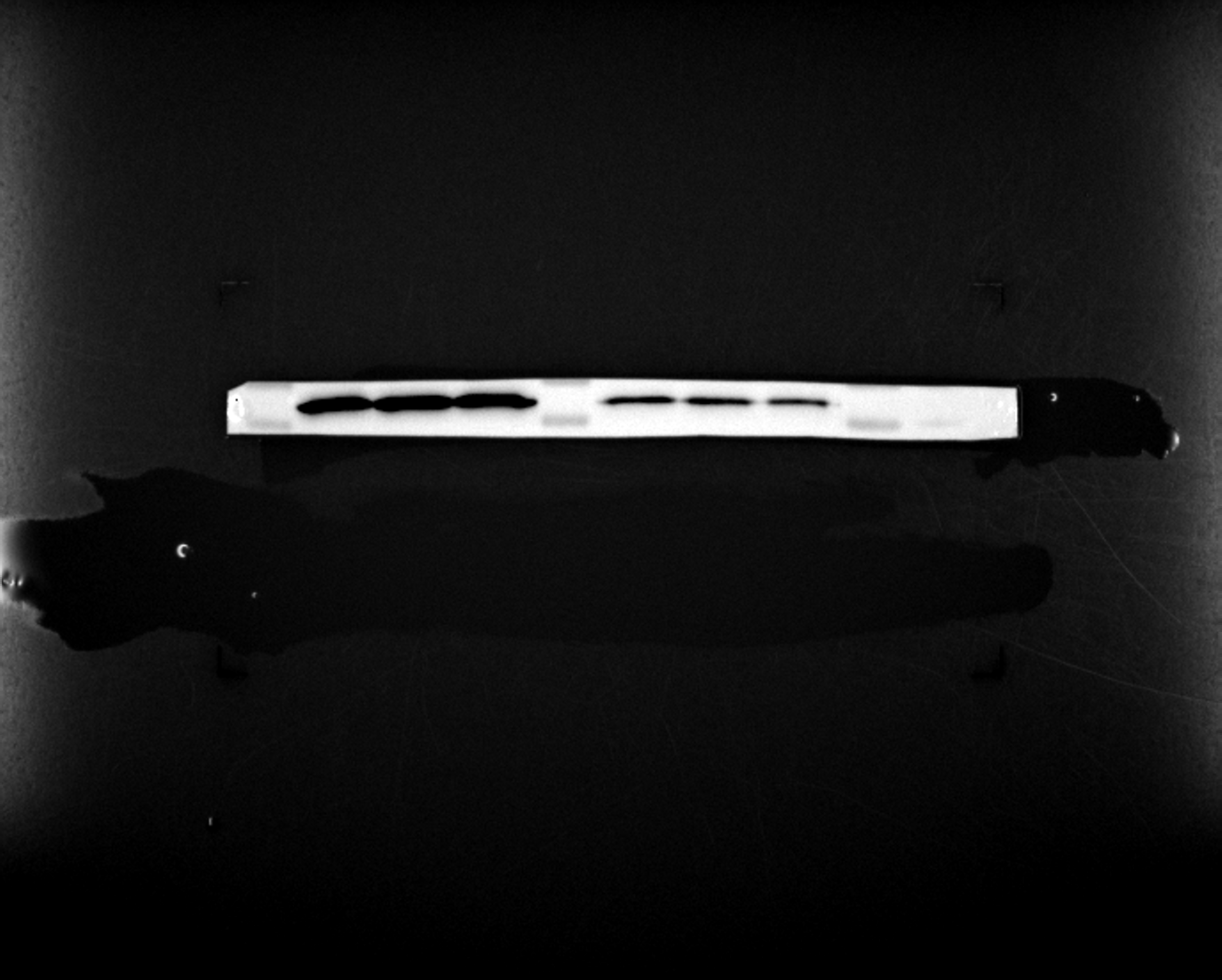

Supplement: Supplementary file 7 [file DataSheet1.zip › raw data-1/Figure 4/4-H A549-KO-CD147-PR8黏附/A549-KO-M1黏附 入侵-Gapdh.Tif]

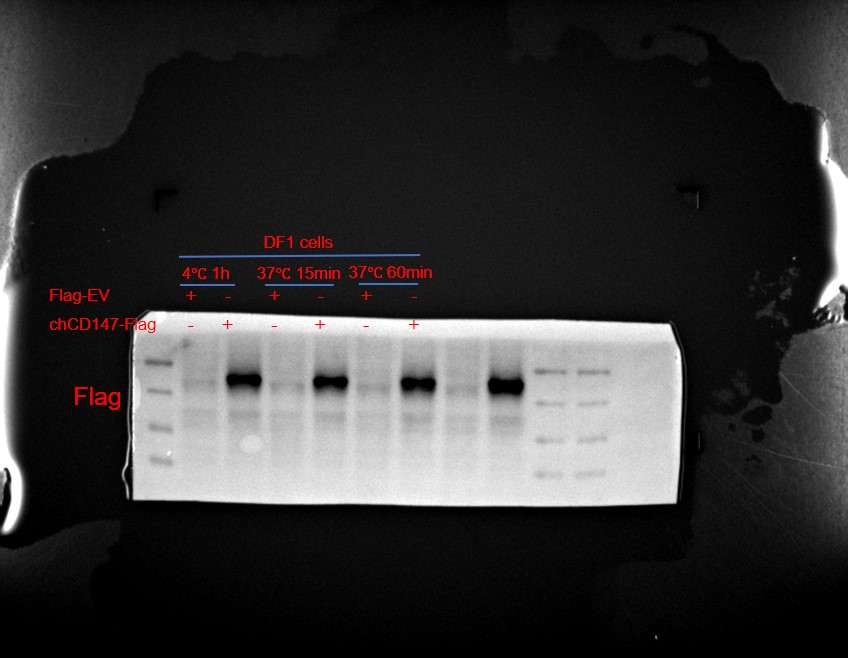

Supplement: Supplementary file 7 [file DataSheet1.zip › raw data-1/Figure 4/Fingure4 B/Flag.jpg]

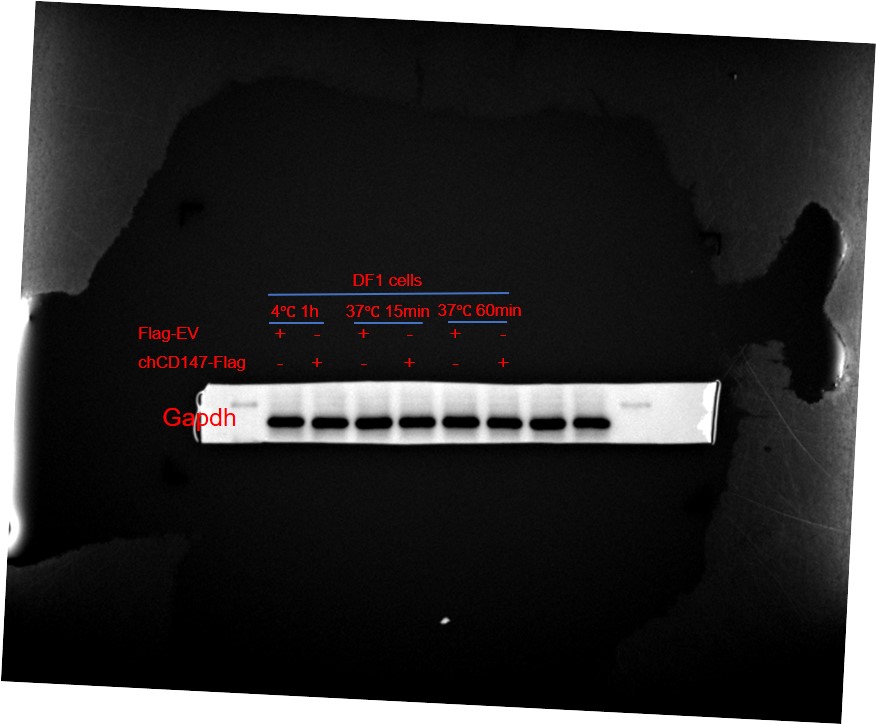

Supplement: Supplementary file 7 [file DataSheet1.zip › raw data-1/Figure 4/Fingure4 B/GAPDH.jpg]

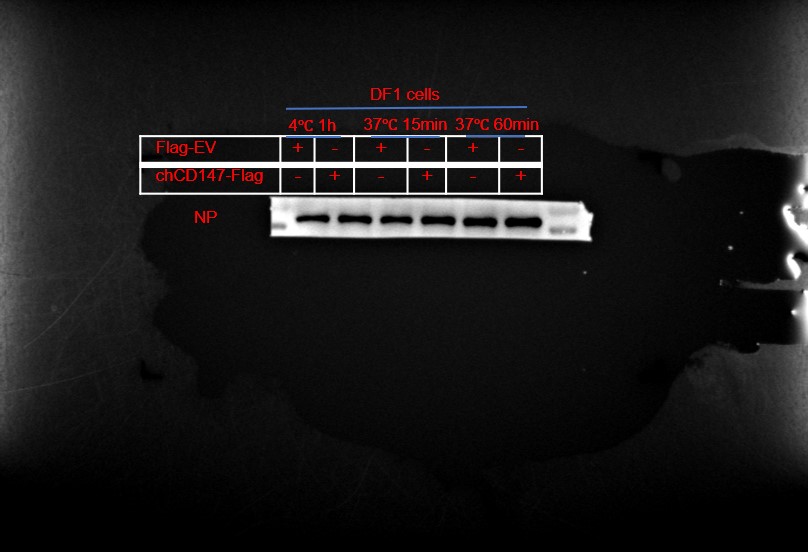

Supplement: Supplementary file 7 [file DataSheet1.zip › raw data-1/Figure 4/Fingure4 B/NP.jpg]

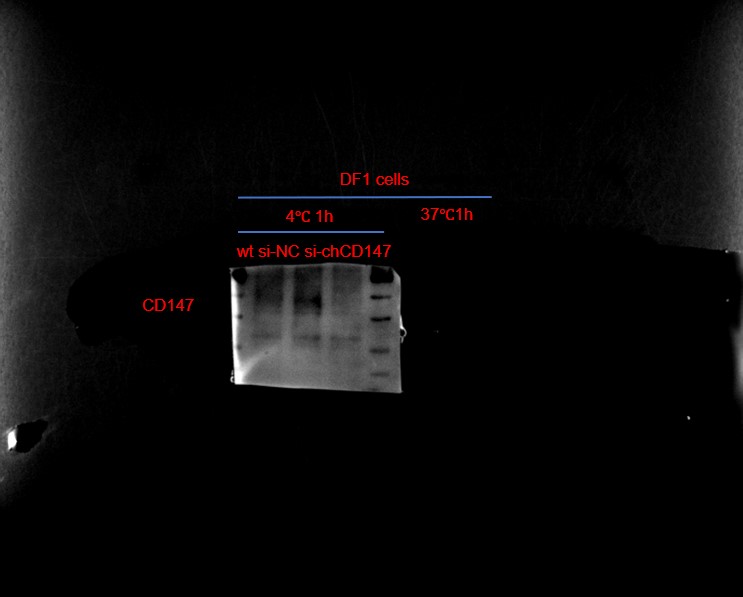

Supplement: Supplementary file 7 [file DataSheet1.zip › raw data-1/Figure 4/Fingure4 D/CD147.jpg]

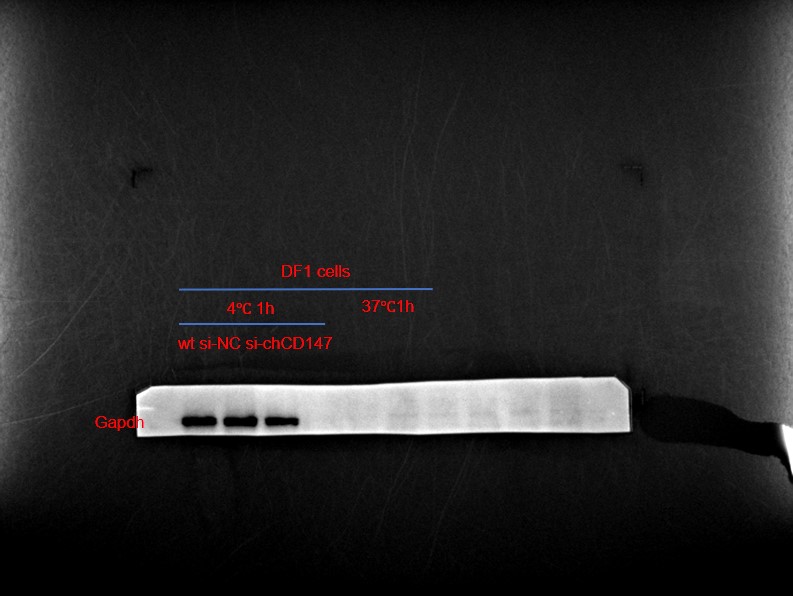

Supplement: Supplementary file 7 [file DataSheet1.zip › raw data-1/Figure 4/Fingure4 D/GAPDH 1.jpg]

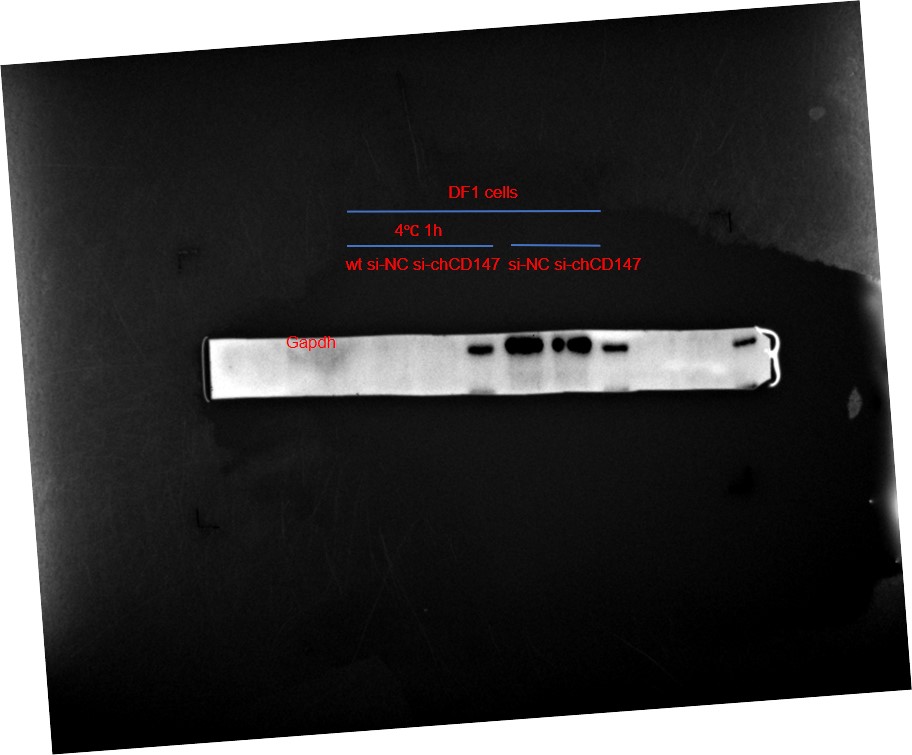

Supplement: Supplementary file 7 [file DataSheet1.zip › raw data-1/Figure 4/Fingure4 D/GAPDH 2.jpg]

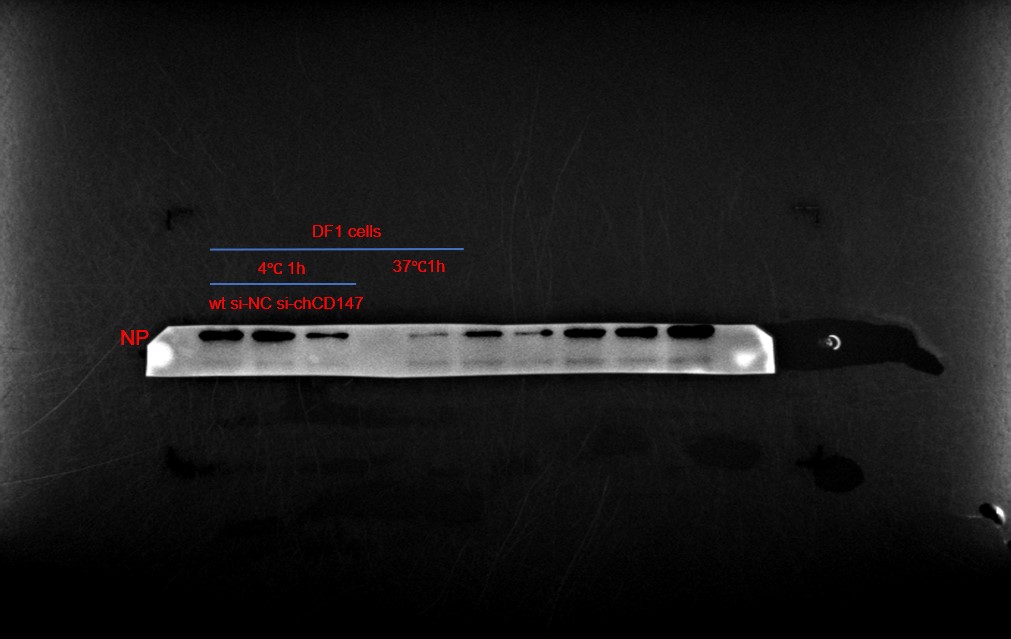

Supplement: Supplementary file 7 [file DataSheet1.zip › raw data-1/Figure 4/Fingure4 D/NP-1.jpg]

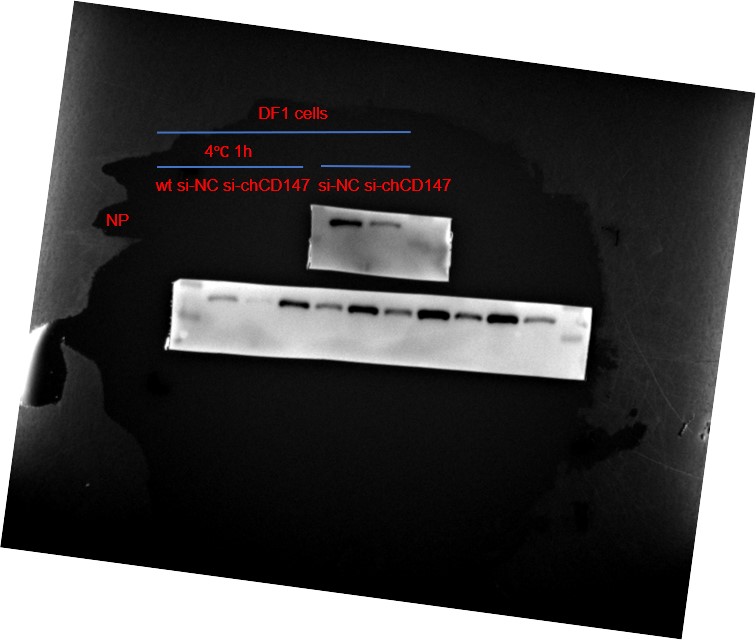

Supplement: Supplementary file 7 [file DataSheet1.zip › raw data-1/Figure 4/Fingure4 D/NP-2.jpg]

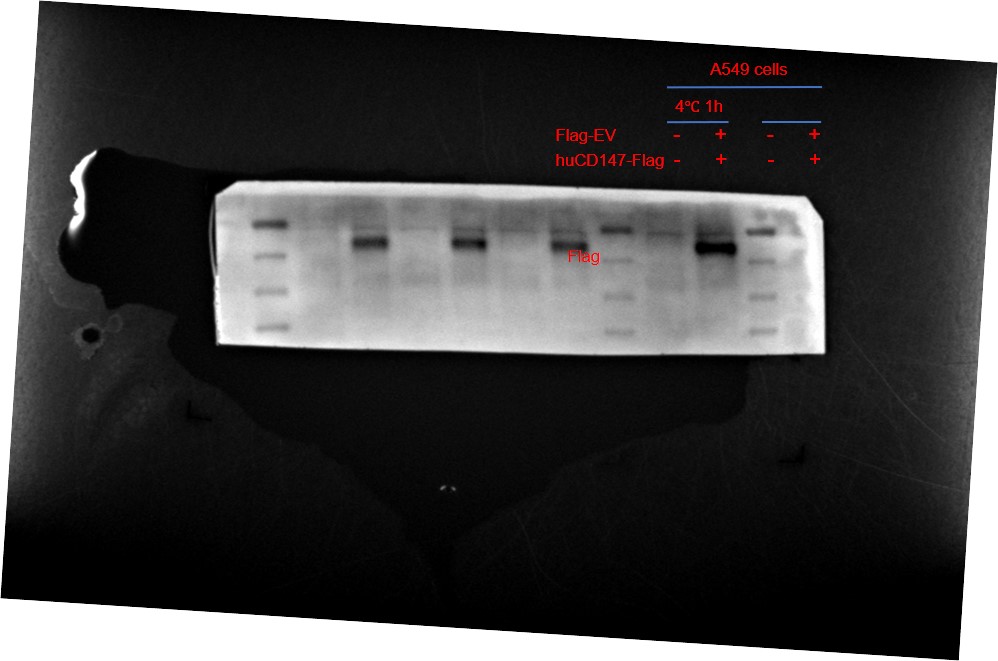

Supplement: Supplementary file 7 [file DataSheet1.zip › raw data-1/Figure 4/Fingure4 F/Flag-1.jpg]
