# Supplementary material for: CD147 mediates the adsorption of influenza A virus on the cell surface through direct interaction with HA
Source: Front Cell Infect Microbiol. 2025 Aug 29;15:1647283. doi: 10.3389/fcimb.2025.1647283 (PMC12426278; doi:10.3389/fcimb.2025.1647283)
Supplement: Supplementary file 4 [file Table1.docx]

**A/chicken/Zhe Jiang/2023010/2023（H9N2）HA** **gene sequence**：

ATGGAGACAGTATCACTAATAACTATACTAGTAGTGGCAACAGTAAGCAATGCAGATAAGATCTGCATCGGCTATCAATCAACAAACTCCACGGAAACTGTGGACACACTAACAGAAAACAATGTCCCTGTAACACATGCCAAAGAACTGCTCCACACAGAGCATAATGGGATGCTGTGTGCAACAAGCTTGGGACATCCTCTTATTCTAGACACCTGCACCATTGAAGGTCTAATTTATGGCAATCCTTCTTGTGATCCATTGCTGGGAGGAAGAGAATGGTCTTATATCGTCGAGAGGCCATCAGCCGTTAACGGATTATGTTATCCCGGGAATGTAGAAAATCTAGAAGAGCTAAGATCACTTTTTAGTTCTTCTAGGTCTTATCAAAGGATCCAGATCTTTCCAGACACAATCTGGAATGTGTCTTACAGTGGGACAAGCAAAGCATGCTCGGATTCATTCTACAGAAGCATGAGATGGTTGACTCAAAAGAACAACGCTTACCCTACCCAAGATGCTCAATACACAAATAATCAAGGAAAGAACATTCTTTTCATGTGGGGTATAAATCATCCACCCACCGATACTGTGCAGACAAATCTGTACACCAGAACCGACACAACAACGAGTGTGGCAACAGAGGAAATGAATAGGGTCTTCAAACCATTGATAGGACCAAGGCCTCTTGTCAACGGTCCGATGGGAAGAATTAATTATTATTGGTCGGTATTGAAACCGGGCCAAACACTGCGGATAAAATCTGATGGGAATCTAATAGCTCCATGGTATGGACACATCCTTTCAGGAGAGAGCCACGGAAGAATCCTAAAGACTGACTTAAAAATGGGTAGCTGCACAGTGCAGTGTCAAACAGAGAAAGGTGGCTTAAACACAACATTGCCCTTCCAAAATGTAAGTAAGTATGCATTTGGAAACTGCTCAAAGTACATTGGTGTAAAGAGTCTCAAACTTGCAGTTGGTCTGAGGAATGTGCCTTCTAGATCTAGCAGAGGACTATTTGGGGCCATAGCAGGATTTATAGAGGGAGGTTGGTCAGGACTGGTTGCTGGTTGGTATGGGTTCCAGCATTCAAATGACCAAGGGGTTGGTATGGCAGCAGATAGAGATTCAACCCAAAAGGCAATTGATAAAATAACATCCAAAGTGAATAATATAGTCGACAAAATGAACAAGCAGTATGAAATCATTGATCATGAGTTCAGTGAGGTAGAAACTAGGCTTAACATGATCAATGATAAGGTTGATGATCAAATCCAAGATATATGGGCATATAATGCAGAATTGCTAGTTCTGCTTGAAAACCAGAAAACACTCGATGAACATGACGCTAATGTAAACAATCTATATAATAAAGTGAAGAGGGCATTGGGTTCCAATGCAGTGGAAGATGGGAGAGGATGTTTCGAGCTATACCACAAATGCGATGACCATTGCATGGAGACAATTCGGAATGGGACCTACAACAGGAGGAAGTATCAAGAGGAATCAAAATTAGAAAGACAGAAAATAGAGGGGGTCAAGCTGGAATCTGAAGAAACTTACAAAATCCTCACCATTTATTCGACTGTCGCCTCATCCCTTGTGATTGCAATGGGGTTTGCTGCCTTTTTGTTCTGGGCCATG
