# Supplementary material for: CD147 mediates the adsorption of influenza A virus on the cell surface through direct interaction with HA
Source: Front Cell Infect Microbiol. 2025 Aug 29;15:1647283. doi: 10.3389/fcimb.2025.1647283 (PMC12426278; doi:10.3389/fcimb.2025.1647283)
Supplement: Supplementary file 5 [file Table2.docx]

GGGACTCAAGACGGCTTCGTCAGGCGCCGCTGTCCCAGCAGAGGTGGGTGGGGGGCAGTGTGGAGCTGCACTGCGAGGCCGTGGGCAGCCCGGTGCCCGAGATCCAGTGGTGGTTTGAAGGGCAGGGTCCCAACGACACCTGCTCCCAGCTCTGGGACGGCGCCCGGCTGGACCGCGTCCACATCCACGCCACCTACCACCAGCCAGCACCATCTCCATCGACACGCTCGTGGAGGAGGACACGGGCACTTACGAGTGCCGGGCCAGCAACGACCCGGATCGCAACCACCTGACCCGGGCGCCCAGGGTCAAGTGGGTCCGCGCCCAGGCAGTCGTGCTAGTCCTGAAACGGTGAGTGAAT
