# Supplementary material for: CD147 mediates the adsorption of influenza A virus on the cell surface through direct interaction with HA
Source: Front Cell Infect Microbiol. 2025 Aug 29;15:1647283. doi: 10.3389/fcimb.2025.1647283 (PMC12426278; doi:10.3389/fcimb.2025.1647283)
Supplement: Supplementary file 6 [file Table3.docx]

**Table S1 Primers related to avian genes**

| **Primer name** | **Primer sequence（5’-3’）** | **Application** |
| --- | --- | --- |
| ATP6V1A-Flag | aaAAGCTTatggacttctccaagctaccaaaaatcc | Eukaryotic expression vector |
|  | ccGGATCCgtcttccagactgcgaaatgca |  |
| VPS26A-Flag | ccAAGCTTatgagttttcttggaggcttcttcg |  |
|  | ccGGATCCcatctcaggctgctcagcag |  |
| chCD147-Flag | AAAGCTAGCACatggcggcgggcgcggacgt |  |
|  | aaCCGGGATCCgttagcattcctctgg |  |
| NCL-Flag | aaAAGCTTatggtgaagctcgccaagg |  |
|  | ccGGATCCttcaaacttgatcttcttcccttgtgg |  |
| BCAP31-Flag | cccAAGCTTatgagtctgcagtggacggtc |  |
|  | ccGGATCCctcctcctttttgtctcgg |  |
| CHMP5-Flag | aaGCGGCCGCatgaggcacctcctgttggc |  |
|  | ccGGATCCcaactcatccttctctgctgct |  |
| ARF4-Flag | AAGCTTatgggcctcaccatctcctc |  |
|  | ccGGATCCgcgttttgagagttcgtttg |  |
| CCT2-Flag | AAGCTTatgcagtctctctcgccgtc |  |
|  | GGATCCacaggggcgatggtctg |  |
| ARF1-Flag | ccAAGCTTatgctatgtttaaaggcaaggatttc |  |
|  | GGATCCtttctggtttcggagctgattgg |  |
| HSPA5-Flag | GCGGCCGCatgaggcacctcctgttggc |  |
|  | GGATCCcaactcatccttctctgctgctt |  |
| HSPA8-Flag | AAGCTTatgtcaaagggaccagctgttg |  |
|  | TCTAGAatccacctcctcaatggttggtc |  |
| ILKAP-Flag | AAGCTTatggacctgttcggggacc |  |
|  | GGATCCgtgctctatccggaccacca |  |
| VPS29-Flag | AAGCTTatgacacacttcatggctgc |  |
|  | ccGGATCCagattttttgtactcaattctttctactttc |  |
| cRAC1A-Flag | AAGCTTatgcaggccatcaagtgtgtg |  |
|  | GGATCCcagcagcagacattttctcttcc |  |
| CASP1-Flag | AAGCTTatggcagaccaggagctg |  |
|  | GGATCCgaggcctgggaagagatagaact |  |
| ASPH-Flag | AAGCTTatggcctcccgcaagggc |  |
|  | GGATCCaatagcaggaagggttcgtctc |  |
| LIMA1-Flag | GCGGCCGCatggagccttccccgtttaat |  |
|  | cGGATCCttcttcatcatcatcttcatagtaacgg |  |
| HB-Flag | AAGCTTatggaggggaccctggag |  |
|  | GGATCCggatgccagcttgtgcac |  |
| ARF6-Flag | AAGCTTatgggcaaggtgctatccaag |  |
|  | ccGGATCCggatttataattggacgttaaccatgtc |  |
| KDELR3-Flag | AAGCTTatgaacatcttccgcatcctgg |  |
|  | TCTAGAaacaggcattggaaggctt |  |
| SLC25A3-Flag | aaGCGGCCGCatgttctcgtccatcgcgc |  |
|  | ccGGATCCctgagttaaaccaagcttcttcttc |  |
| chBSG-si1 | CCUCAUCAAGGGUCACAAATT | siRNA |
|  | UUUGUGACCCUUGAUGAGGTT |  |
| chBSG-si2 | GCGCUGGCAUCUACAACAUTT |  |
|  | AUGUUGUAGAUGCCAGCGCTT |  |
| chBSG-si3 | GCAUUCUCAAACUGAACAUTT |  |
|  | AUGUUCAGUUUGAGAAUGCTT |  |
| ysichBSG-1 | GCACCTCCCACCCTCATCAA | Verify siRNA |
|  | CTACCTTCCCCTCGATCGTGT |  |
| ysichBSG-2 | GAAGTGCTACGGTGAATCTGC |  |
|  | CATCTGGCTTCCTCCTCTTCTC |  |
| ysichBSG-3 | AAGAGGAGGAAGCCAGATGAG |  |
|  | AAGTTCTTCAGCTCCACGTCA |  |
| chCD147mt47-F | CAGCTGTcagATAAGTGCACCTCCCACCCTC | Glycosylation site mutation |
| chCD147mt47-R | CACTTATctgACAGCTGAGGACCACCTTGTCA |  |
| chCD147mt106-F | CAAAAGGAcagGTGTCTATAGAAGTTGAACCCCAAGTC |  |
| chCD147mt106-R | AGACACctgTCCTTTTGCCACTGGGTTTG |  |
| chCD147mt164-F | CATCTACcagATCTCACGCACTGGCAACAAG |  |
| chCD147mt164-R | GTGAGATctgGTAGATGCCAGCGCTACTCTCA |  |
| chCD147mt170-F | ACTGGCcagAAGACAGAGCTTCGCATTCTCAA |  |
| chCD147mt170-R | TCTGTCTTctgGCCAGTGCGTGAGATGTTGTAG |  |
| chCD147mt191-F | cagGGCACCAACATGAAGGGCT |  |
| chCD147mt191-R | TTCATGTTGGTGCCctgGCAGCTGTAGTCACCCATATCCT |  |
| chCD147-ECD-Strep-R | aaTCTAGATTTTTCGAACTGCGGGTGGCTCCcagattcaccgtagcacttccagag | Expression soluble protein |
| rt-chGapdh | TGGGTGTCAACCATGAGAAA | RT-qPCR |
|  | CATCCACCGTCTTCTGTGTG |  |

**Table S2 Primers related to human gene**

| **Primer name** | **Primer sequence（5’-3’）** | **Application** |
| --- | --- | --- |
| huCD147-Flag | aaAAGCTTATGGCGGCTGCGCTGTTC | Glycosylation site mutation |
|  | aaTCTAGAGGAAGAGTTCCTCTGGCGGAC |  |
| huCD147mt44-F | TCACCTGCTCCTTGCCTGACAGCGCCACAGA |  |
| huCD147mt44-R | TCTGTGGCGCTGTCAGGCAAGGAGCAGGTGA |  |
| huCD147mt152-F | ACAAGGCCCTCATGCCCGGCTCCGAGAG |  |
| huCD147mt152-R | CTCTCGGAGCCGGGCATGAGGGCCTTGT |  |
| huCD147mt186-F | cagGGCACCAGCTCCAAGGGCtccgaccagg |  |
| huCD147mt186-R | TGGAGCTGGTGCCGGGGCACCGGTACTGG |  |
| huCD147-1TM-R | aaTCTAGAGTAGATGAAGATGATGGTGACCAGCACCAGCACCTCAGCCACGATGCCCAGGAAGGGCCAGAGGGCCCCGTGGAGCTGGATGT | Truncated expression |
| huCD147 sig-Ig2-F | aaAAGCTTATGGCGGCTGCGCTGTTCGTGCTGCTGGGATTCGCGCTGCTGGGCACCCACGGAGCCTCCGGGgctGTGAAGTCGTCAGAACACATC |  |
| huCD147-ECD-F | aaATCGGGTACCATGGCGGCTGCGCTGTTCGTGCTGCTG | expression Soluble protein |
| huCD147-Ig1-ECD-strep | TTTCTCGAACTGCGGGTGGCTCCAGGCCAGGTGGCTGCGCACGCG |  |
| huCD147-Ig2-ECD-strep | GTGCGCAGCCACCTGGCCTGGAGCCACCCGCAGTTCGAGAAAGGT |  |
| pCAGGS-strepII-end-R | aaATCGCTCGAGTTATCATTTCTCGAACTGCGGGTGGCTCCACGA |  |
| huCD147-sg1 | CACCGCTTGAATGACAGCGCCACAG | sgRNA |
|  | aaacCTGTGGCGCTGTCATTCAAGC |  |
| huCD147-sg2 | aaacAGGTCTTCTACGGTAGTGAAC |  |
|  | CACCGTTCACTACCGTAGAAGACCT |  |
| yhuCD147-sg1 | CGCCTCCGCCGCTTTTTATAG | Verify sgRNA |
|  | ACCTCTAGTCCCCCCTCAAAC |  |
| yhuCD147-sg2 | CAGGCACTAACAAGACCCCAC |  |
|  | CACTCACGTTCCAGGACTAGC |  |
| rt-PR8-NP | CAACTTATCAGAGGACAAGAG | RT-qPCR |
|  | GACCAATTCCATCACCATT |  |
| rt-huGapdh | GGAGCGAGATCCCTCCAAAAT |  |
|  | GGCTGTTGTCATACTTCTCATGG |  |

**Table S3 Primers related to Influenza virus genes**

| **Primer name** | **Primer sequence（5’-3’）** | **Source** | **Application** |
| --- | --- | --- | --- |
| PCA-H1-0-ha-F | AGCGTAATCTGGAACATCGTATGGGTA | H1N1  (PR8) | Truncated expression |
| PCA-H1-0-ha-R | CCGctcgagtCTGGTAGATGCCCATACTCTCCAA |  |  |
| PCA-H1-1-1-ha-R | CCGctcgagAAGTTGTTCCCTCAGTTCCTCATAG |  |  |
| PCA-H1-1-RBD-F | CCatcgatATGAAGGCTAACCTGCTGGTGCTGCTGTGTGCCCTGGCTGCTGCTGATGCTTCCTCCTTTGAGAGGTTTGA |  |  |
| PCA-H1-1-RBD-ha-R | AAActcgagGGCATACATAGGGGCAATCAGG |  |  |
| PCA-H1-1-3-F | CCatcgatATGAAGGCTAACCTGCTGGTGCTGCTGTGTGCCCTGGCTGCTGCTGATGCTTTTGCCCTGAGCAGG |  |  |
| PCA-H1-1-3-ha-R | AAActcgagtGCTCTGGATGCTTGGGATGT |  |  |
| PCA-H1-2-ha-F | CCatcgatCTGTTTGGAGCCATTGCTGGC |  |  |
| PCA-H1-1-1-ha-R | CCGctcgagTGGTGATGGTGATGATGAAGTTGTTCCCTCAGTTCCTCATAG |  | Expression of soluble protein |
| PCA-H1-1-RBD-ha-R | AAActcgagTGGTGATGGTGATGATGGGCATACATAGGGGCAATCAGG |  |  |
| PCA-H1-1-3-ha-R | AAActcgagtTGGTGATGGTGATGATGGCTCTGGATGCTTGGGATGT |  |  |
| PCA-H1-2-his-R | AAActcgagATGGTGATGGTGATGATGGATGCCCATACTCTCCAATTTCACTCC |  |  |
| PCA-H5-F | CCatcgat ATGAAGGAGATCGTGCTGCTGC | H5N6  (JX) | Eukaryotic expression |
| PCA-H5-ha-R | AAActcgagAGCGTAATCTGGAACATCGTATGGGTA GCACATCCACAGGCTCAGGC |  |  |
| H5-ACS-F | ACAGCCCCCTGAGAGAGACTAGAGGCCTGTTCGGCGCCATCGCCG |  | Mutation shearing site |
| H5-ACS-R | TGGCGCCGAACAGGCCTCTAGTCTCTCTCAGGGGGCTGTTTCTCAGG |  |  |
| PCA-H5-His-R | AAActcgagATGGTGATGGTGATGATGCTGGTAGGTGCCGATGCTCTCCAGCT |  | Expression of soluble protein |
| PCA-H9-F | CCatcgat ATGGAGACCGTGAGCCTGATC | H9N2 | Eukaryotic expression |
| PCA-H9-ha-R | CCGctcgagAGCGTAATCTGGAACATCGTATGGGTA CATGGCCCAAAAGAGGAAGGCT |  |  |
| PCA-H9-His-R | CCGctcgagTGGTGATGGTGATGATG CATGGCCCAAAAGAGGAAGGCT |  | Expression of soluble protein |
| rt-H9N2-NP | AACGACCGGAATTTCTGGAGAGG |  | RT-qPCR |
|  | CCGTACACACAAGCAGGCAAGC |  |  |
