# Supplementary material for: CD147 mediates the adsorption of influenza A virus on the cell surface through direct interaction with HA
Source: Front Cell Infect Microbiol. 2025 Aug 29;15:1647283. doi: 10.3389/fcimb.2025.1647283 (PMC12426278; doi:10.3389/fcimb.2025.1647283)
Supplement: Supplementary file 7 [file DataSheet1.zip › raw data-1/Figure 1/Introduction of data sources Figure 1.docx]

Fig. 1A is a pattern diagram drawn by the author.

Fig. 1B The original data was analyzed by GraphPad Prism 9.0.0.

Fig. 1C TCID_50_ original data is displayed in excel table and histogram is drawn, and the WB original pictures are saved separately.
