# Supplementary figures and images for: CD147 mediates the adsorption of influenza A virus on the cell surface through direct interaction with HA (part 2 of 2)
Source: Front Cell Infect Microbiol. 2025 Aug 29;15:1647283. doi: 10.3389/fcimb.2025.1647283 (PMC12426278; doi:10.3389/fcimb.2025.1647283)

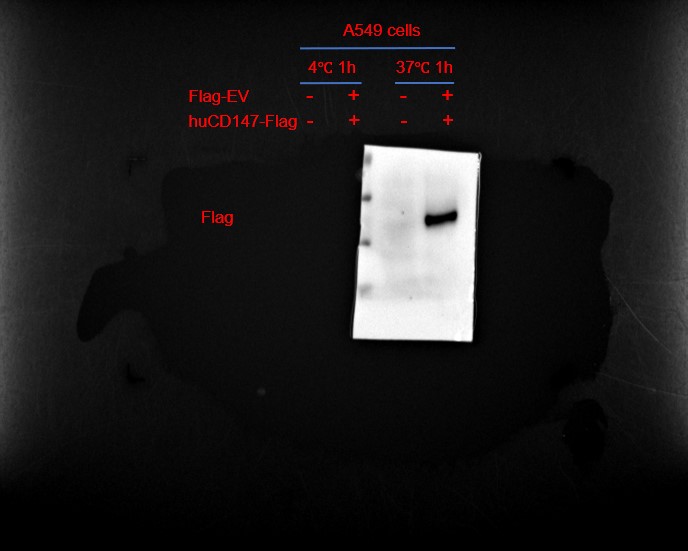

Supplement: Supplementary file 7 [file DataSheet1.zip › raw data-1/Figure 4/Fingure4 F/Flag-2.jpg]

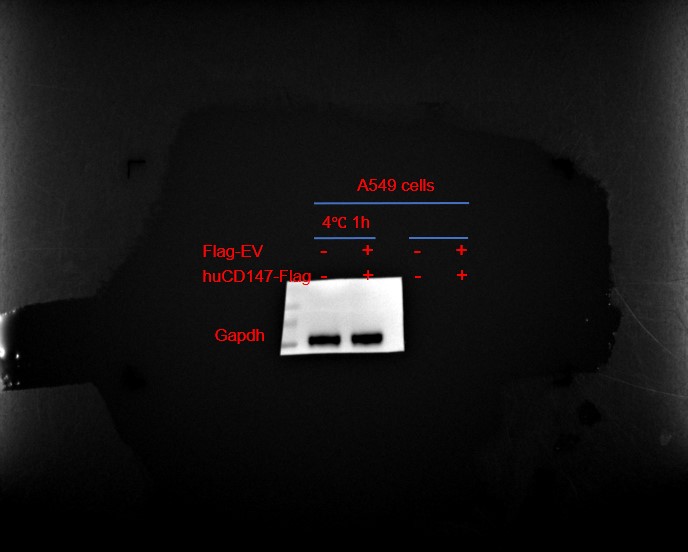

Supplement: Supplementary file 7 [file DataSheet1.zip › raw data-1/Figure 4/Fingure4 F/Gapdh-1.jpg]

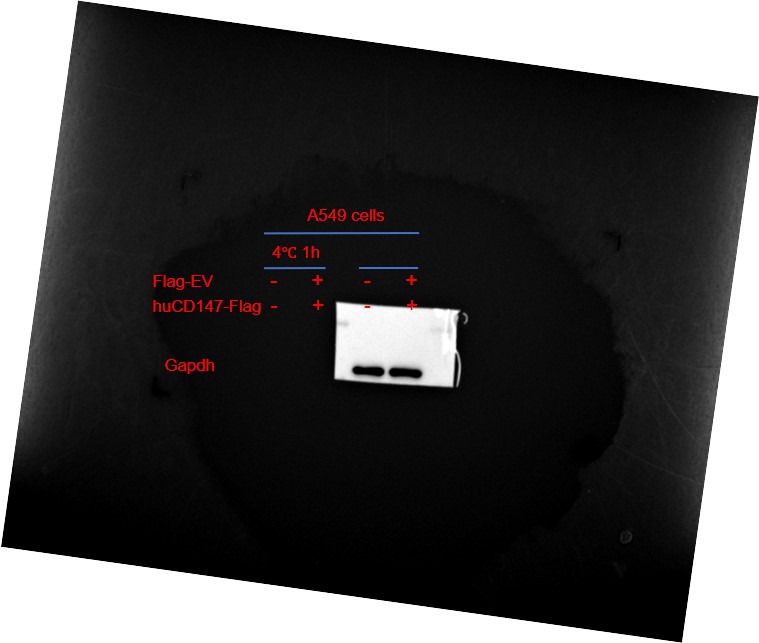

Supplement: Supplementary file 7 [file DataSheet1.zip › raw data-1/Figure 4/Fingure4 F/Gapdh-2.jpg]

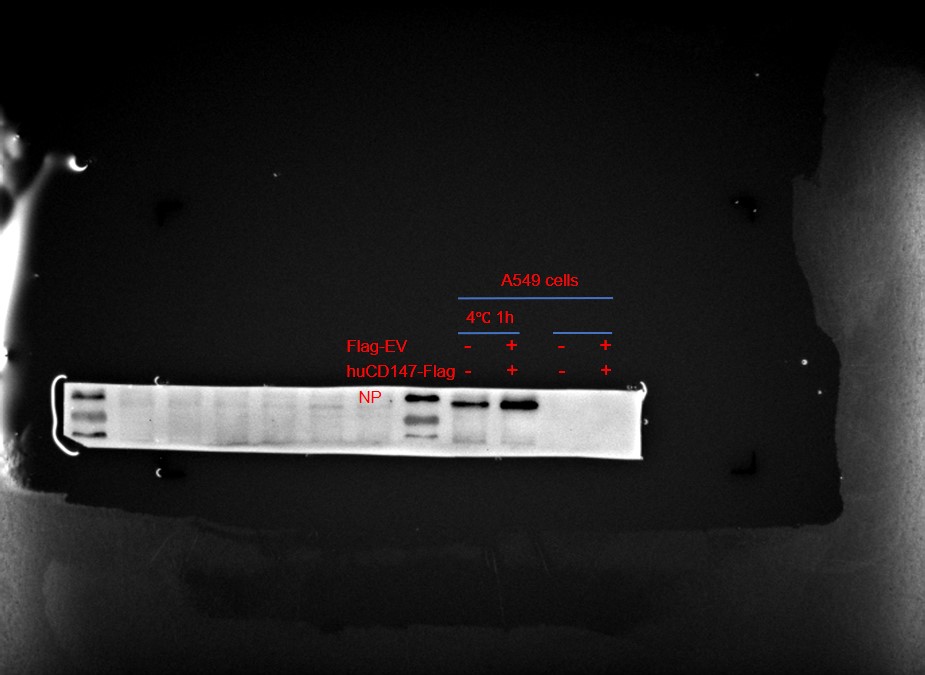

Supplement: Supplementary file 7 [file DataSheet1.zip › raw data-1/Figure 4/Fingure4 F/NP-1.jpg]

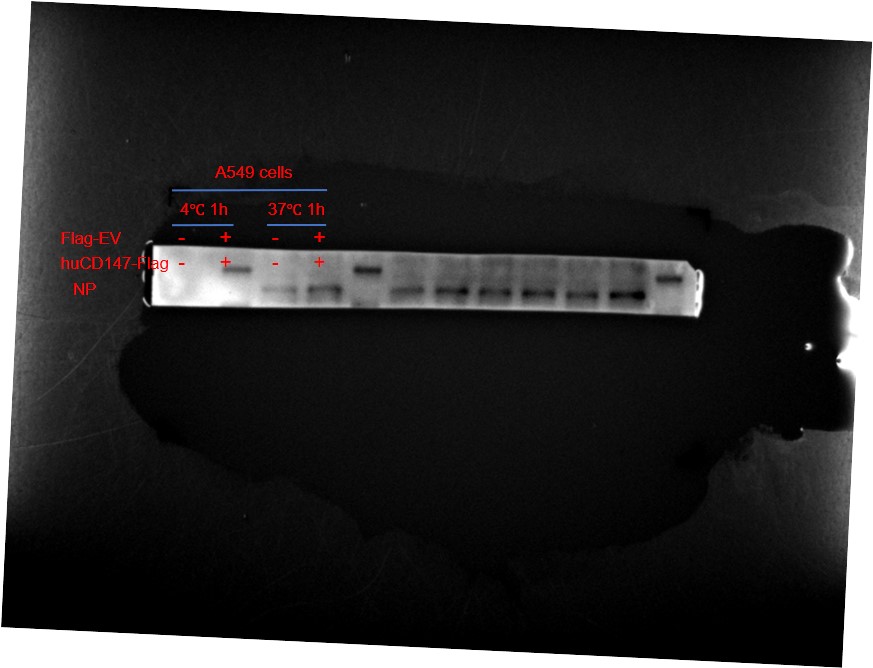

Supplement: Supplementary file 7 [file DataSheet1.zip › raw data-1/Figure 4/Fingure4 F/NP-2.jpg]

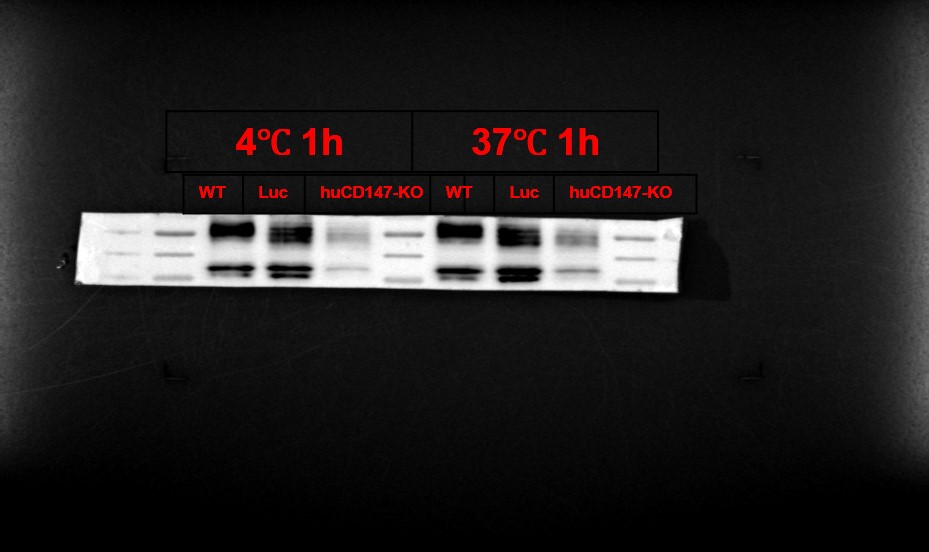

Supplement: Supplementary file 7 [file DataSheet1.zip › raw data-1/Figure 4/Fingure4 H/CD147.jpg]

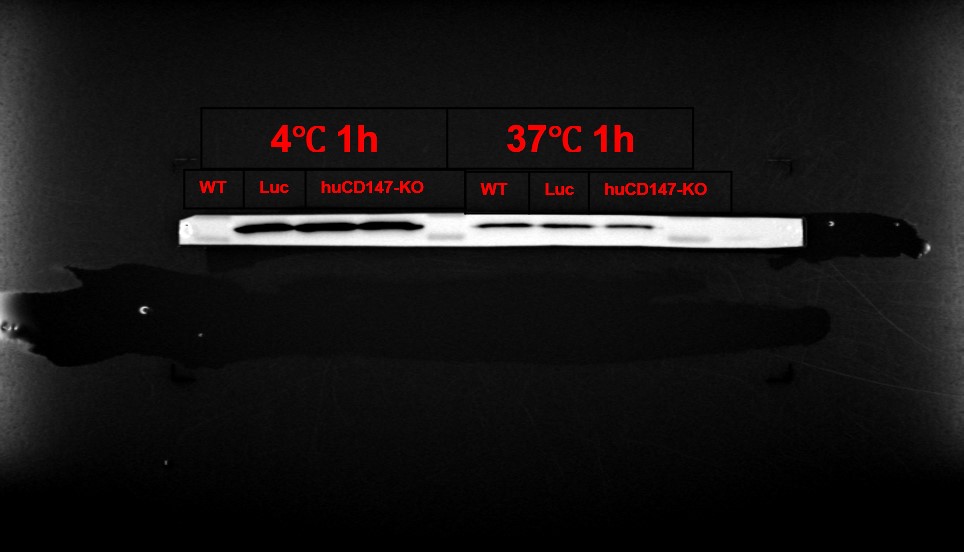

Supplement: Supplementary file 7 [file DataSheet1.zip › raw data-1/Figure 4/Fingure4 H/Gapdh.jpg]

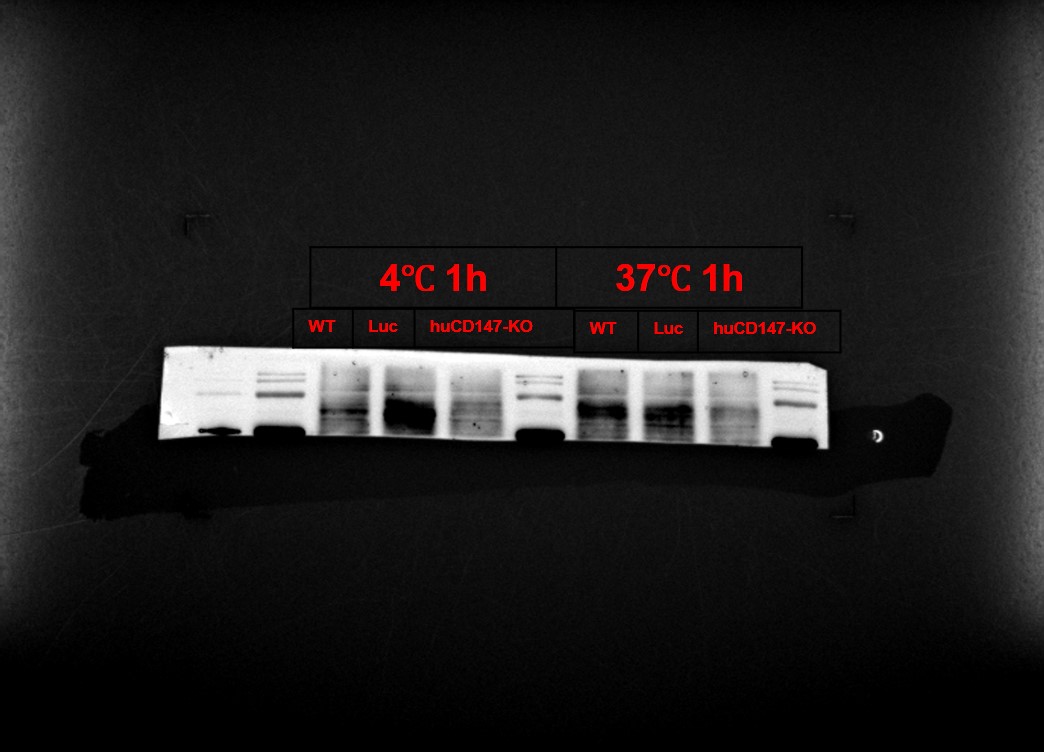

Supplement: Supplementary file 7 [file DataSheet1.zip › raw data-1/Figure 4/Fingure4 H/NP.jpg]

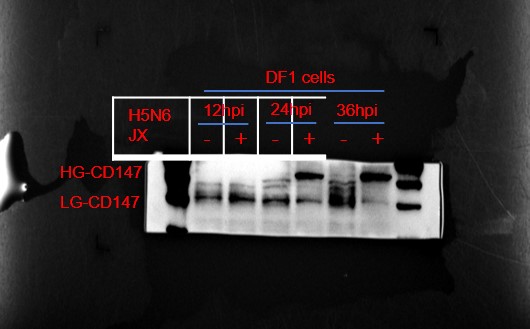

Supplement: Supplementary file 8 [file DataSheet2.zip › raw data-2/Figure 5/5A/CD147.jpg]

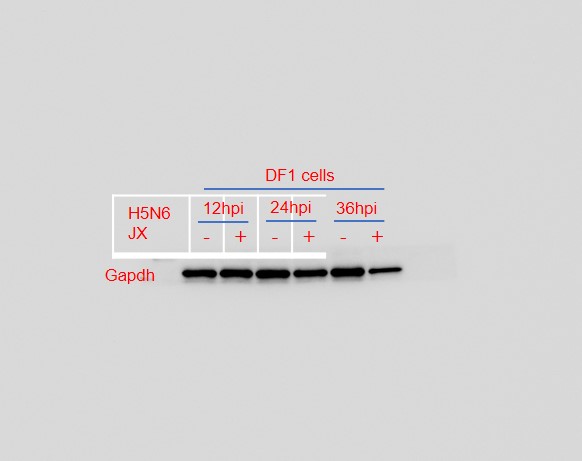

Supplement: Supplementary file 8 [file DataSheet2.zip › raw data-2/Figure 5/5A/Gapdh.jpg]

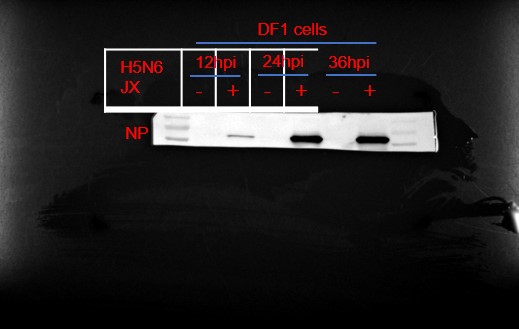

Supplement: Supplementary file 8 [file DataSheet2.zip › raw data-2/Figure 5/5A/NP.jpg]

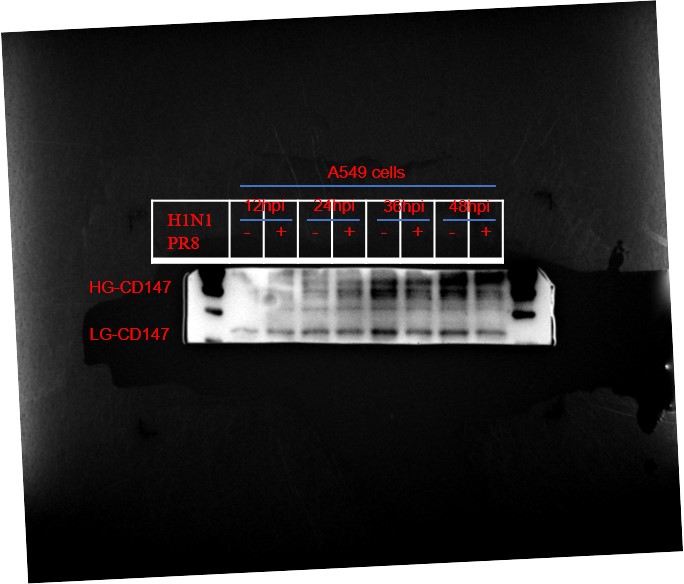

Supplement: Supplementary file 8 [file DataSheet2.zip › raw data-2/Figure 5/5C/CD147.jpg]

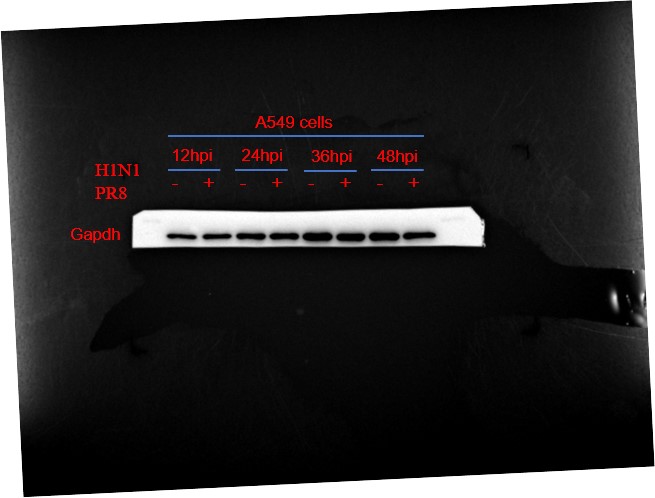

Supplement: Supplementary file 8 [file DataSheet2.zip › raw data-2/Figure 5/5C/Gapdh.jpg]

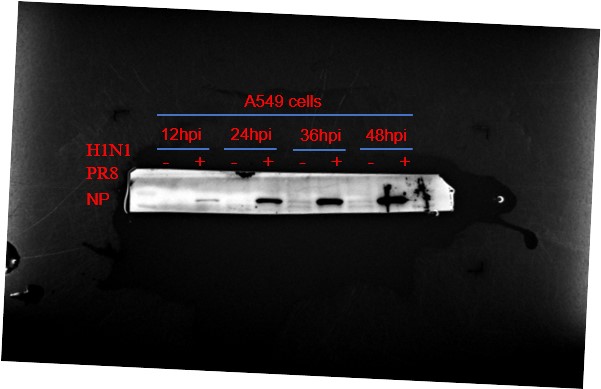

Supplement: Supplementary file 8 [file DataSheet2.zip › raw data-2/Figure 5/5C/NP.jpg]

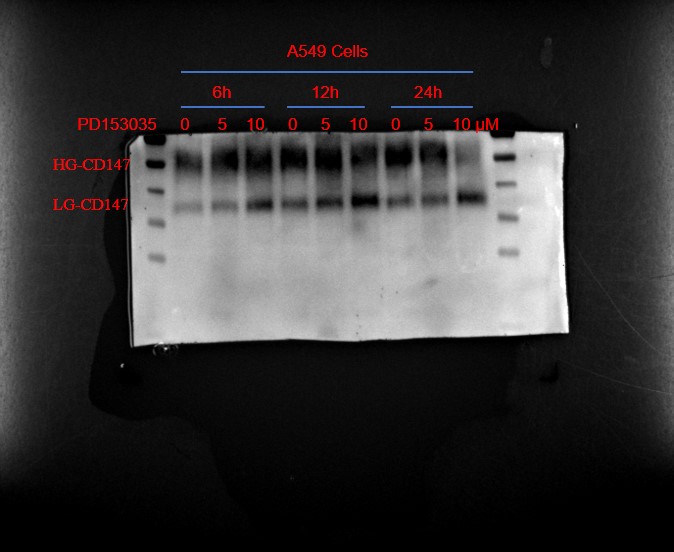

Supplement: Supplementary file 8 [file DataSheet2.zip › raw data-2/Figure 5/5E/CD147.jpg]

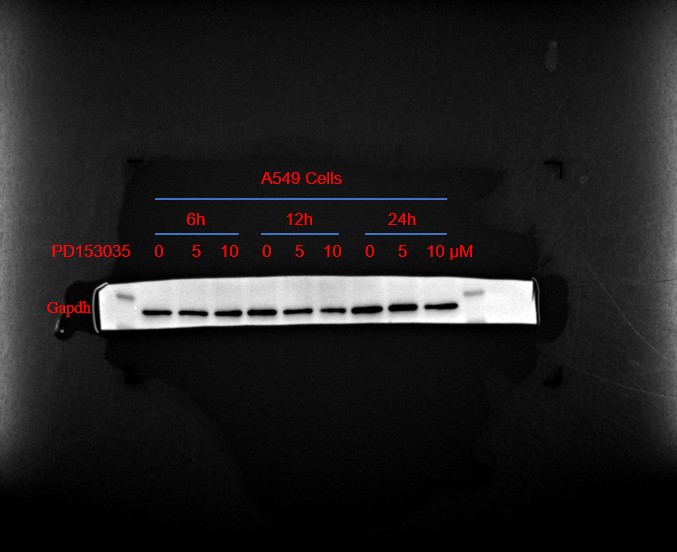

Supplement: Supplementary file 8 [file DataSheet2.zip › raw data-2/Figure 5/5E/Gapdh.jpg]

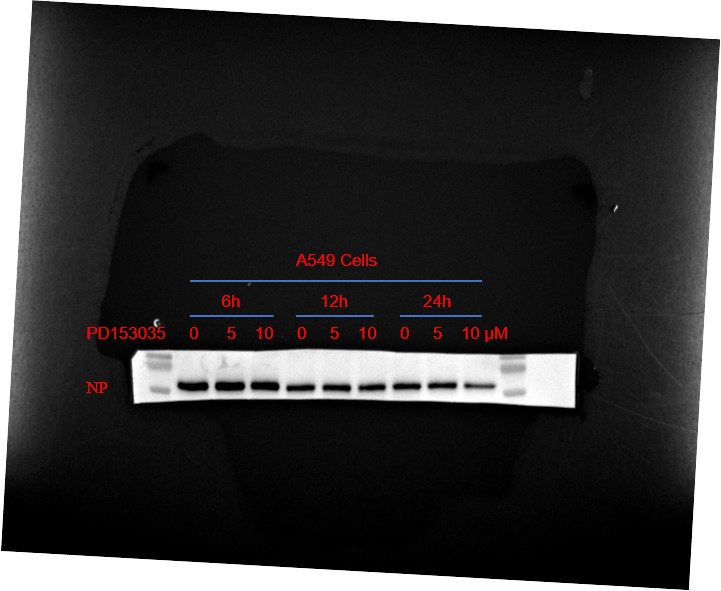

Supplement: Supplementary file 8 [file DataSheet2.zip › raw data-2/Figure 5/5E/NP.jpg]

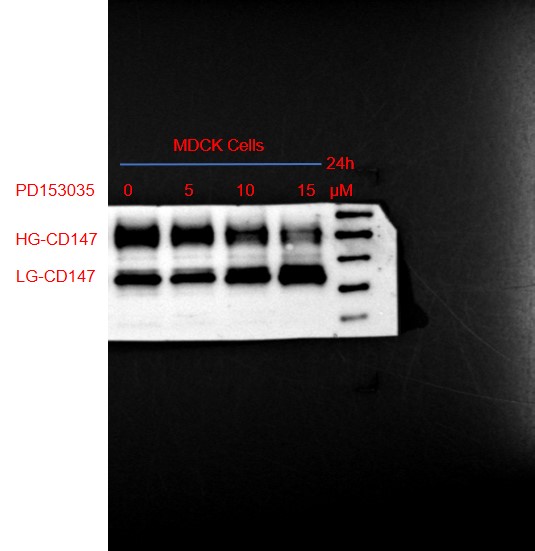

Supplement: Supplementary file 8 [file DataSheet2.zip › raw data-2/Figure 5/5G/CD147.jpg]

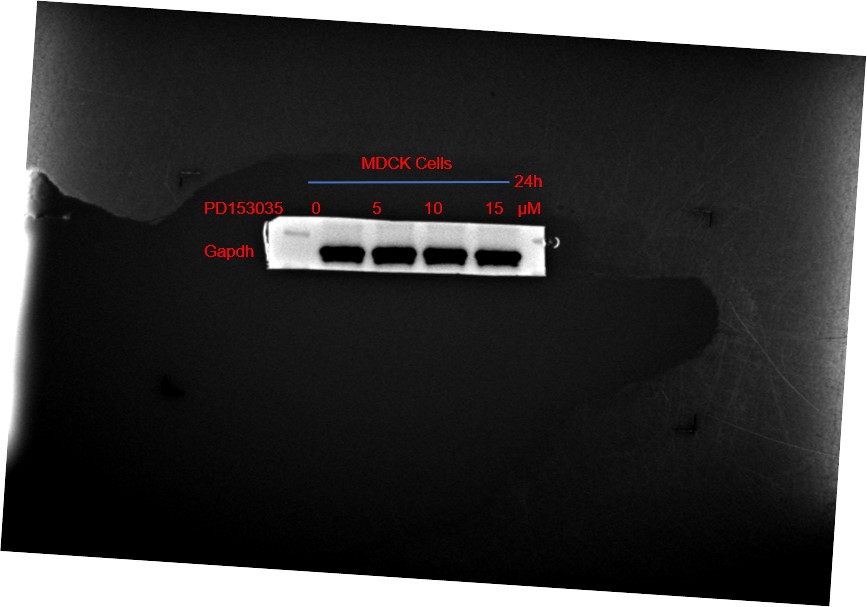

Supplement: Supplementary file 8 [file DataSheet2.zip › raw data-2/Figure 5/5G/Gapdh.jpg]

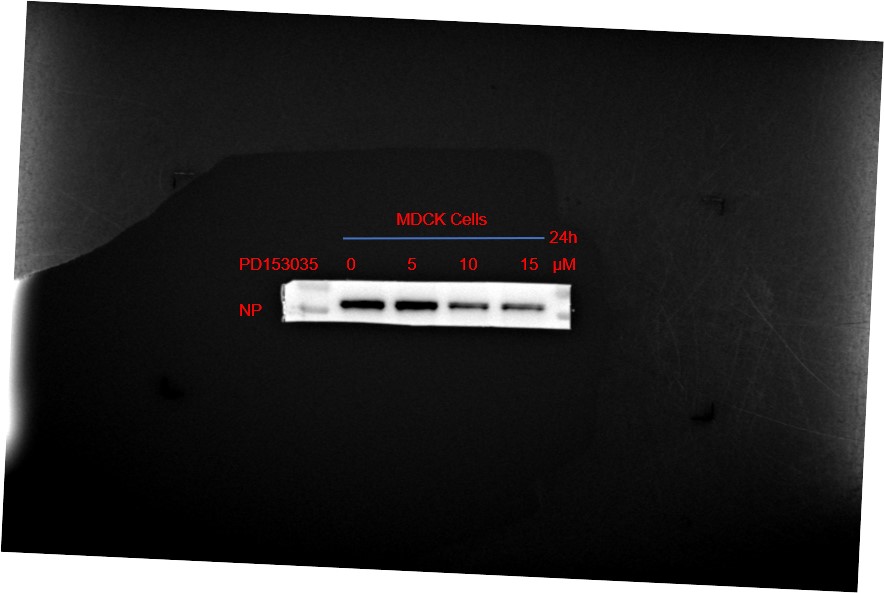

Supplement: Supplementary file 8 [file DataSheet2.zip › raw data-2/Figure 5/5G/NP.jpg]

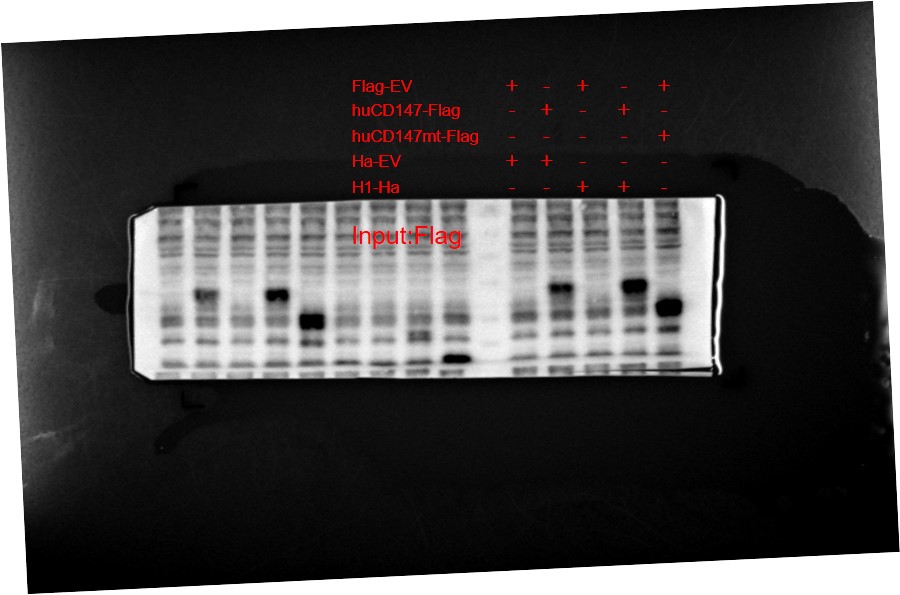

Supplement: Supplementary file 8 [file DataSheet2.zip › raw data-2/Figure 6/Fingure6 A/INPUT Flag.jpg]

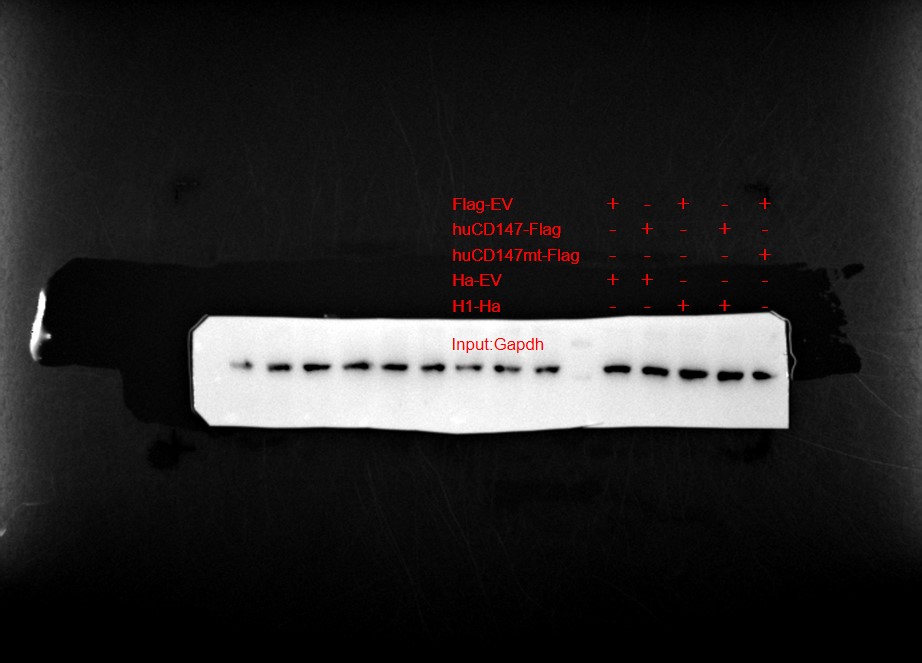

Supplement: Supplementary file 8 [file DataSheet2.zip › raw data-2/Figure 6/Fingure6 A/INPUT Gapdh.jpg]

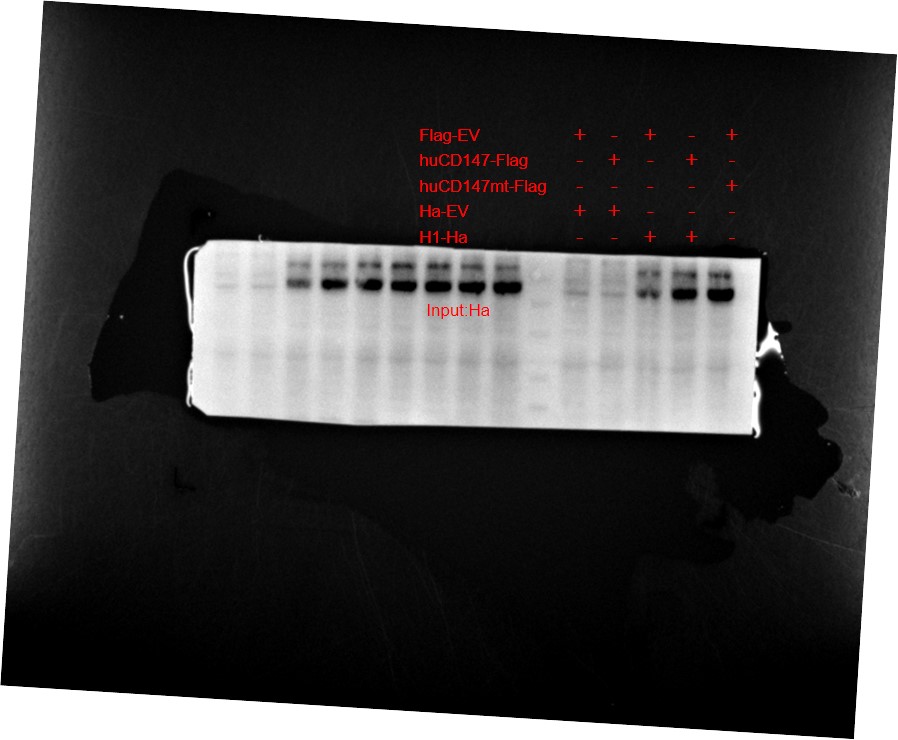

Supplement: Supplementary file 8 [file DataSheet2.zip › raw data-2/Figure 6/Fingure6 A/INPUT Ha.jpg]

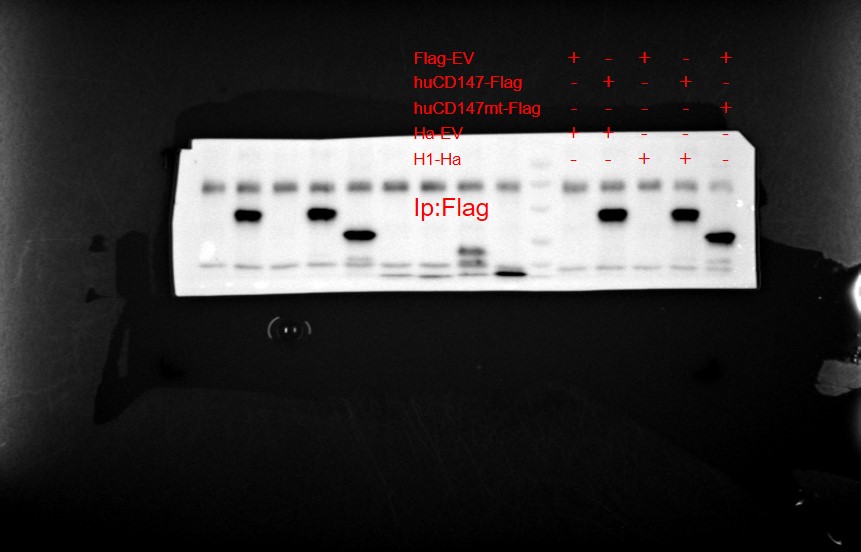

Supplement: Supplementary file 8 [file DataSheet2.zip › raw data-2/Figure 6/Fingure6 A/IP Flag.jpg]

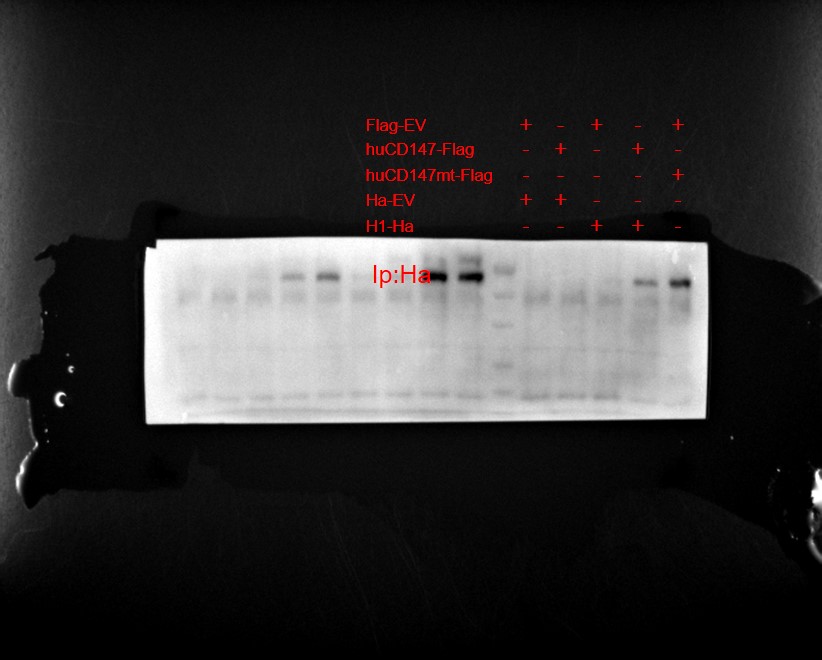

Supplement: Supplementary file 8 [file DataSheet2.zip › raw data-2/Figure 6/Fingure6 A/IP Ha.jpg]

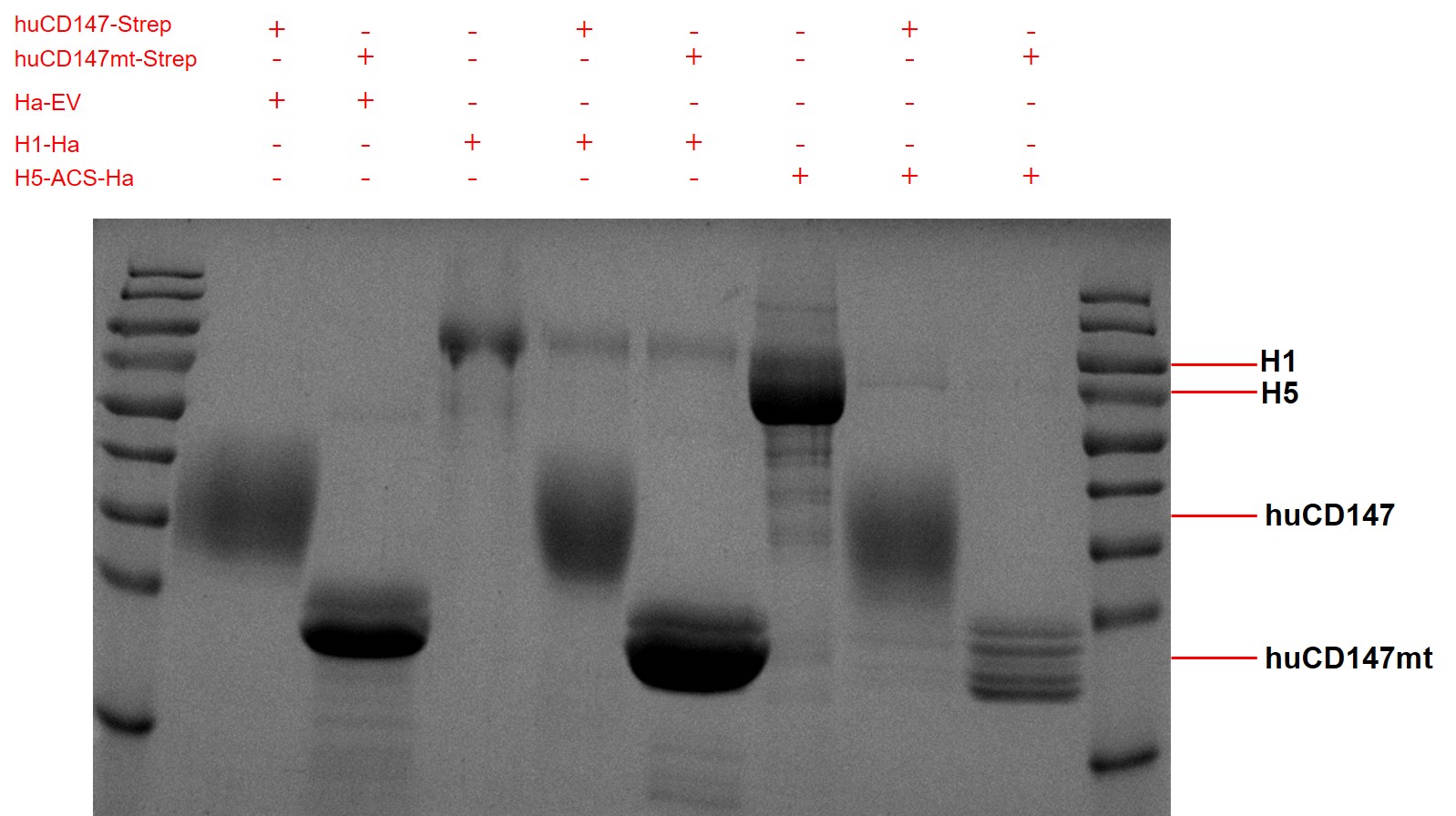

Supplement: Supplementary file 8 [file DataSheet2.zip › raw data-2/Figure 6/Fingure6 B/protein SDS-PAGE.jpg]

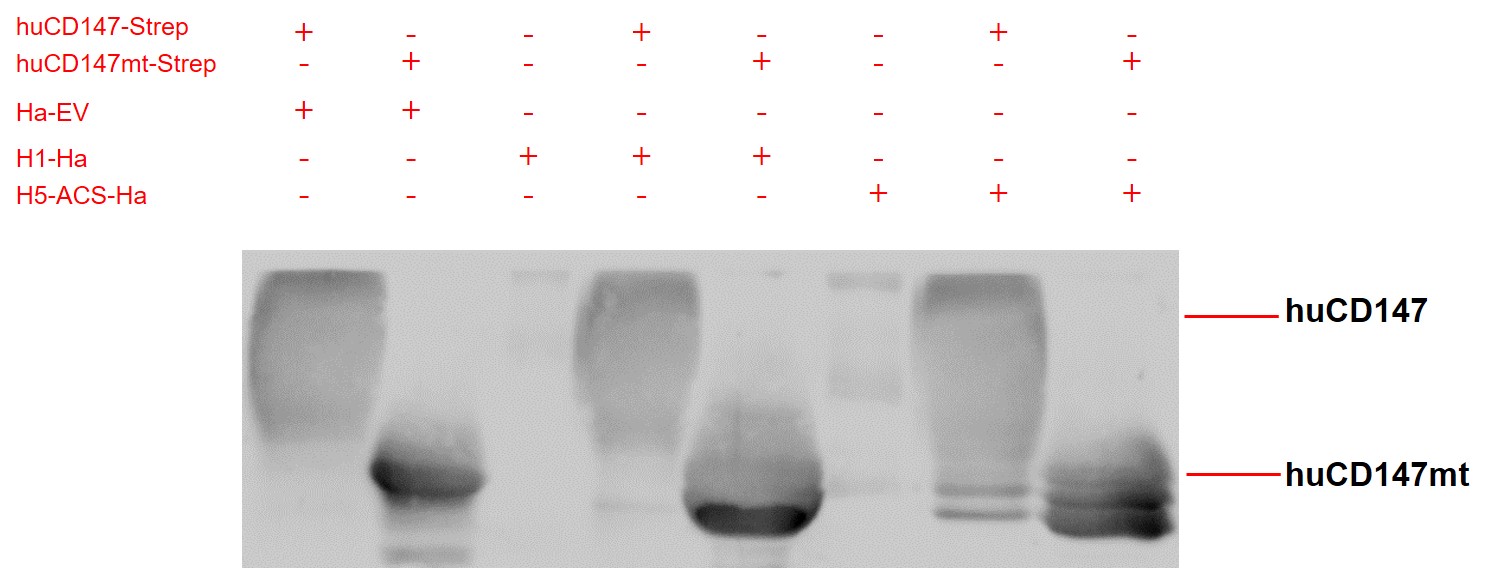

Supplement: Supplementary file 8 [file DataSheet2.zip › raw data-2/Figure 6/Fingure6 B/Strep-pull down-CD147.jpg]

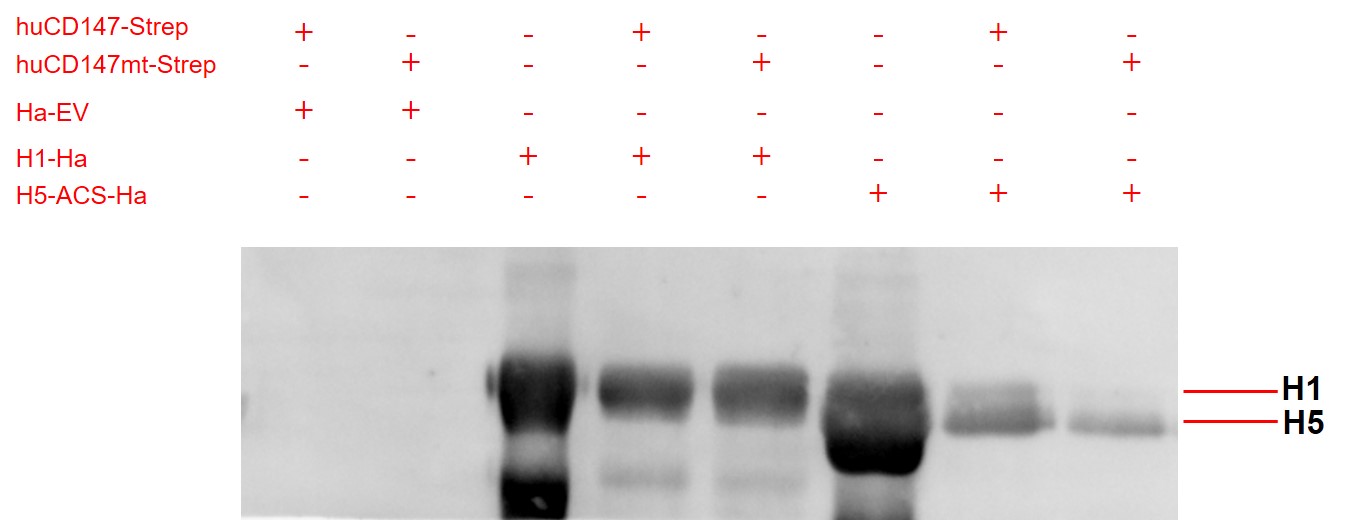

Supplement: Supplementary file 8 [file DataSheet2.zip › raw data-2/Figure 6/Fingure6 B/Strep-pull down-HA.jpg]

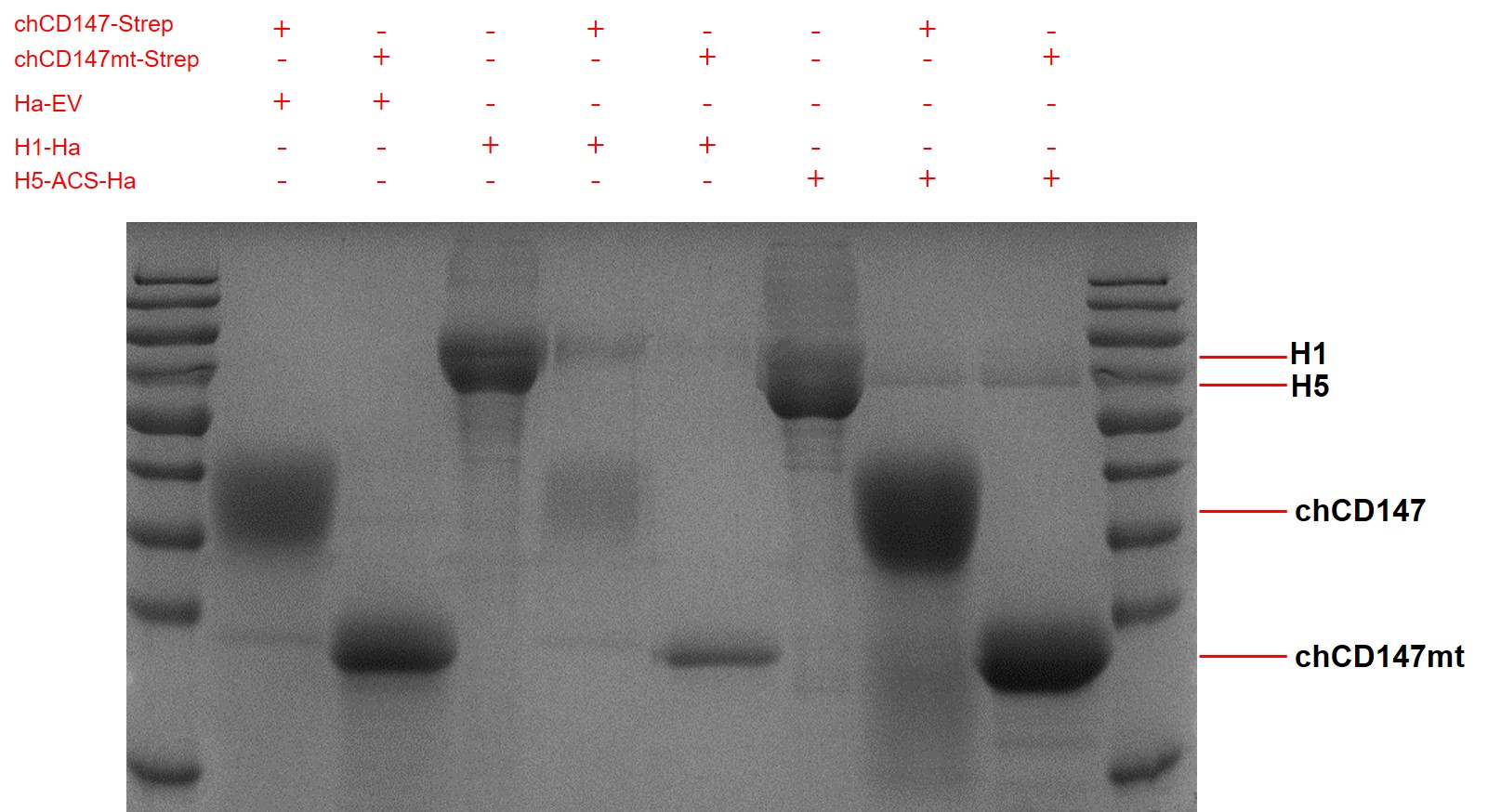

Supplement: Supplementary file 8 [file DataSheet2.zip › raw data-2/Figure 6/Fingure6 C/protein PAGE.jpg]

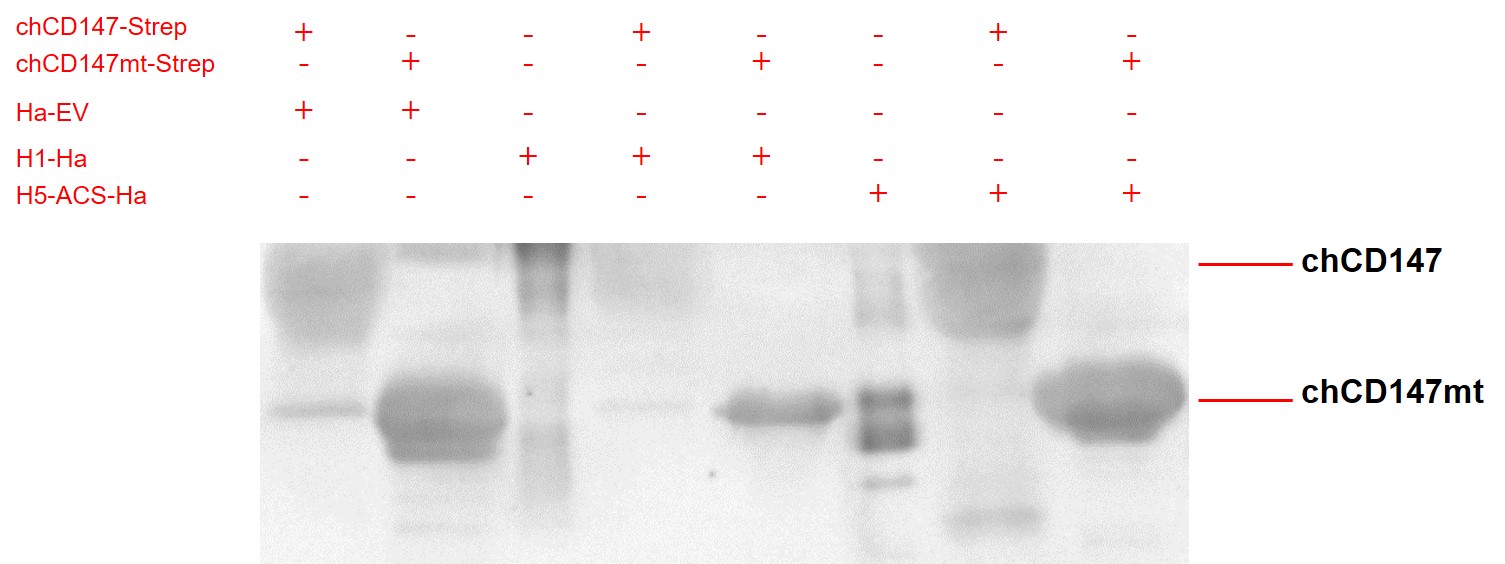

Supplement: Supplementary file 8 [file DataSheet2.zip › raw data-2/Figure 6/Fingure6 C/Strep-pull down-CD147.jpg]

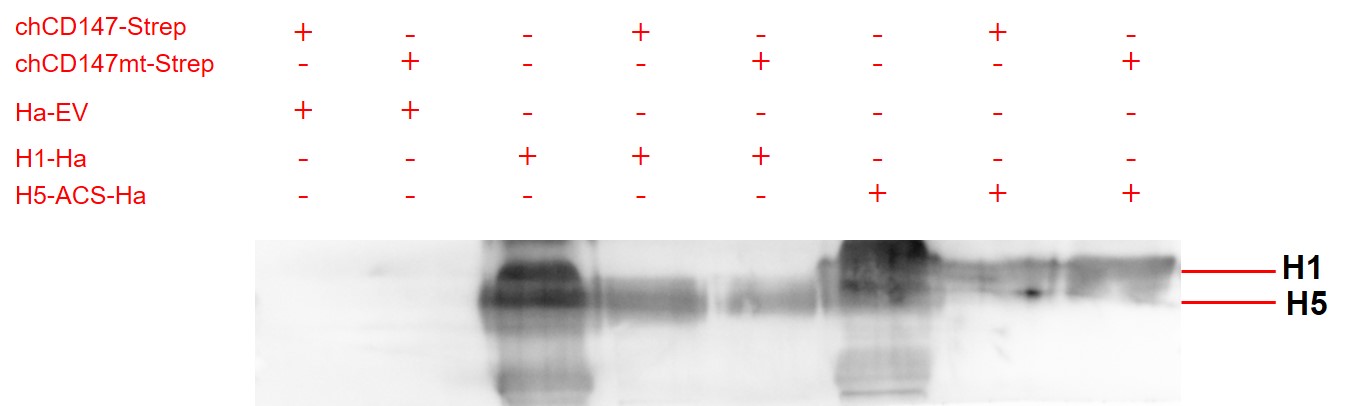

Supplement: Supplementary file 8 [file DataSheet2.zip › raw data-2/Figure 6/Fingure6 C/Strep-pull down-HA.jpg]

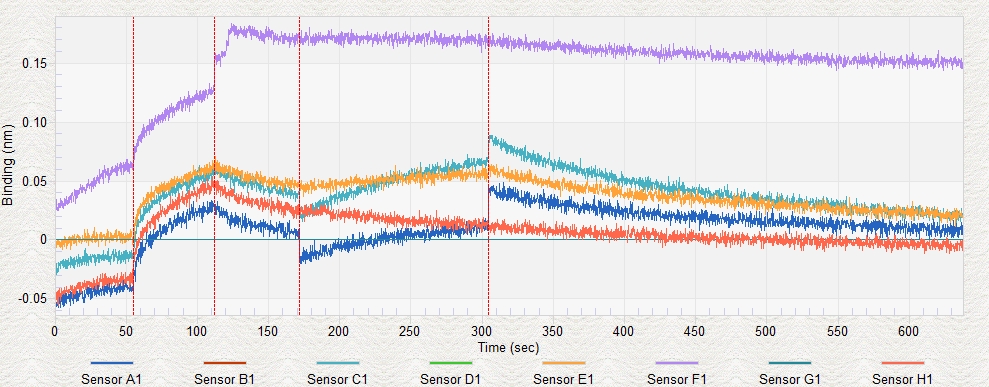

Supplement: Supplementary file 8 [file DataSheet2.zip › raw data-2/Figure 6/Fingure6 D-I/Fingure6 D/241005_Assay_1.jpg]

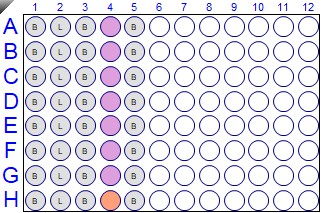

Supplement: Supplementary file 8 [file DataSheet2.zip › raw data-2/Figure 6/Fingure6 D-I/Fingure6 D/Plate1Definition.jpg]

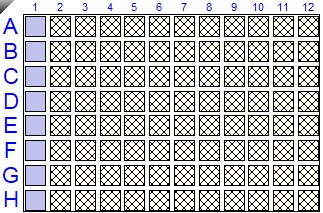

Supplement: Supplementary file 8 [file DataSheet2.zip › raw data-2/Figure 6/Fingure6 D-I/Fingure6 D/SensorPlate.jpg]

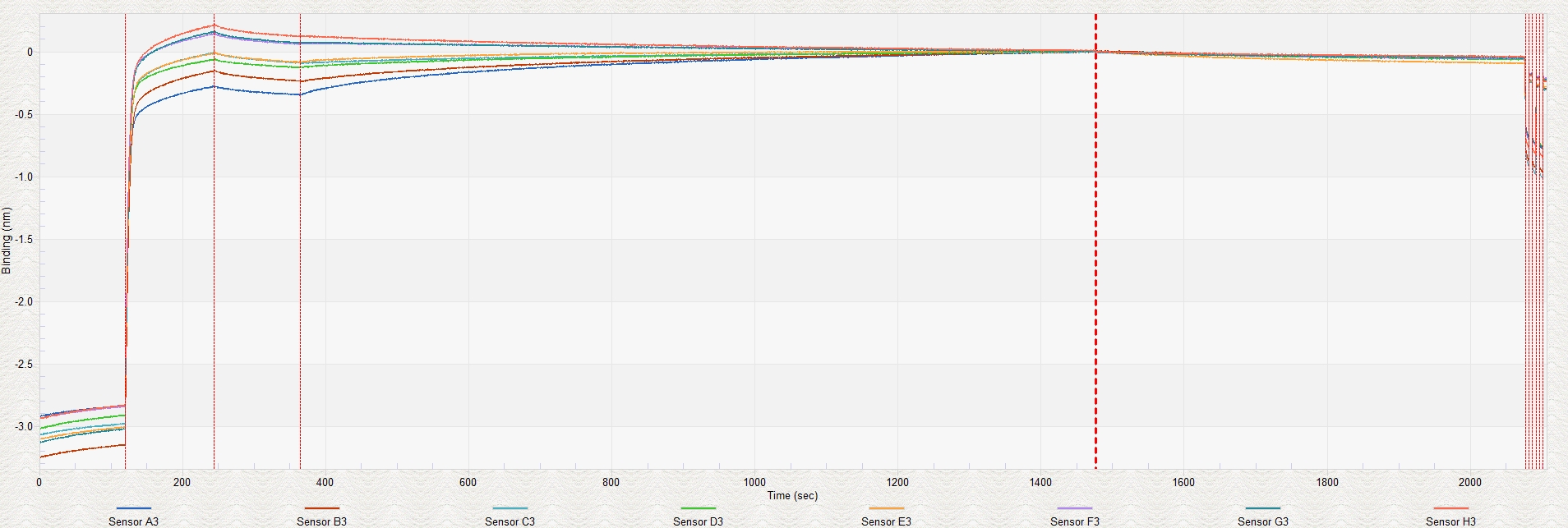

Supplement: Supplementary file 8 [file DataSheet2.zip › raw data-2/Figure 6/Fingure6 D-I/Fingure6 E/241006_Assay_1.jpg]

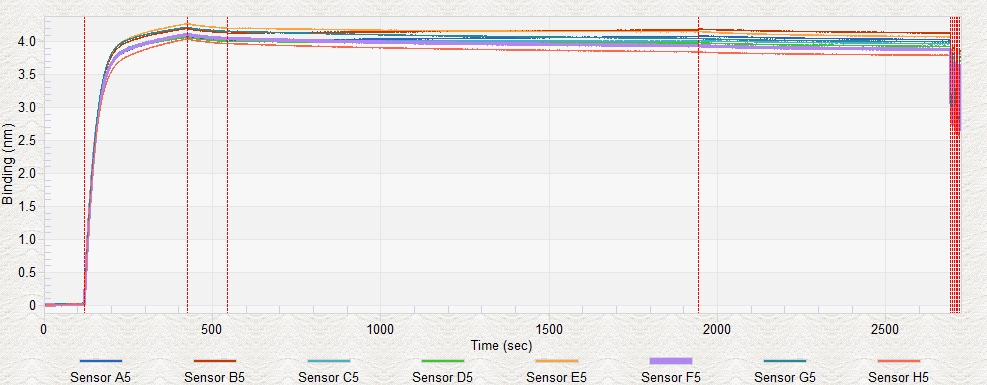

Supplement: Supplementary file 8 [file DataSheet2.zip › raw data-2/Figure 6/Fingure6 D-I/Fingure6 E/241006_Assay_2.jpg]

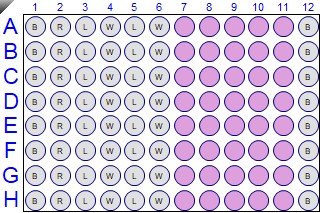

Supplement: Supplementary file 8 [file DataSheet2.zip › raw data-2/Figure 6/Fingure6 D-I/Fingure6 E/Plate1Definition.jpg]

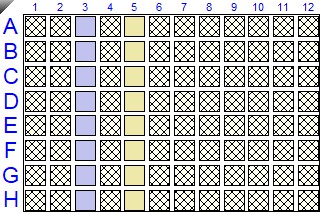

Supplement: Supplementary file 8 [file DataSheet2.zip › raw data-2/Figure 6/Fingure6 D-I/Fingure6 E/SensorPlate.jpg]

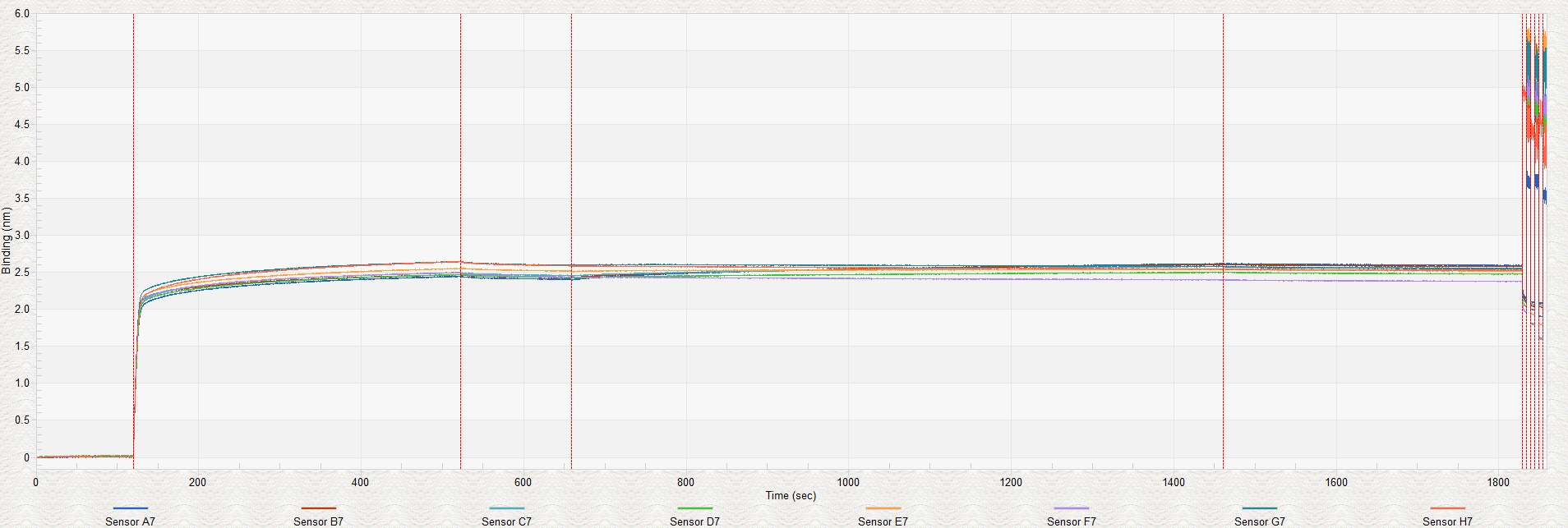

Supplement: Supplementary file 8 [file DataSheet2.zip › raw data-2/Figure 6/Fingure6 D-I/Fingure6 F/241006_Assay_1.jpg]

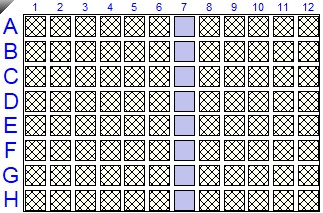

Supplement: Supplementary file 8 [file DataSheet2.zip › raw data-2/Figure 6/Fingure6 D-I/Fingure6 F/SensorPlate.jpg]

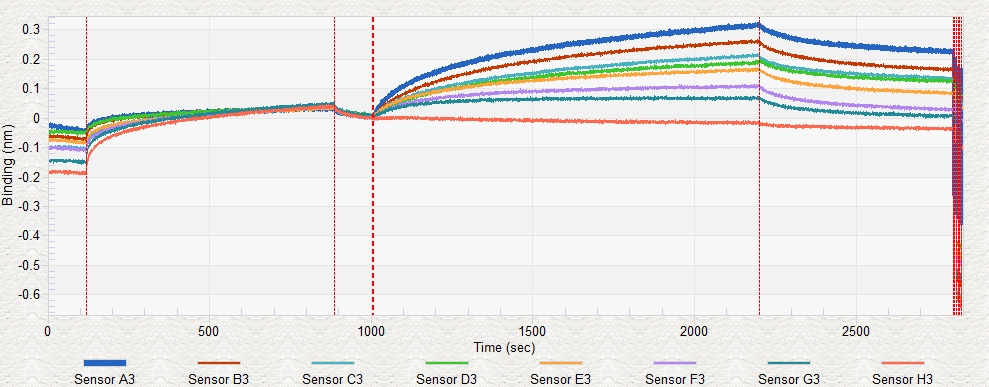

Supplement: Supplementary file 8 [file DataSheet2.zip › raw data-2/Figure 6/Fingure6 D-I/Fingure6 G/241006_Assay_1.jpg]

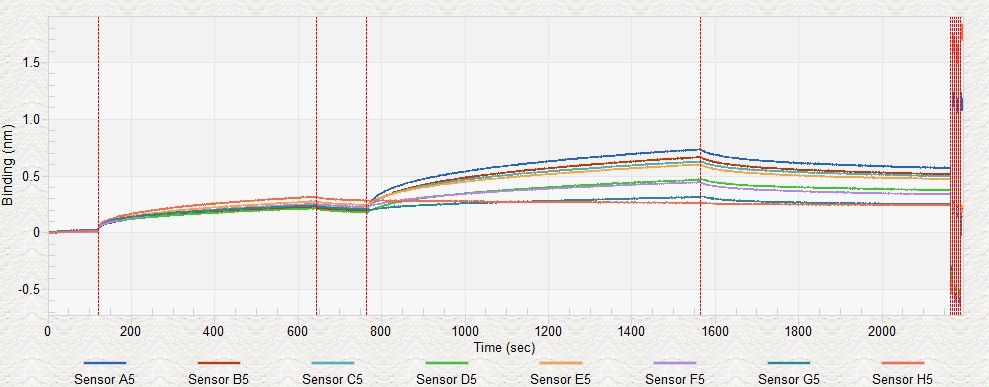

Supplement: Supplementary file 8 [file DataSheet2.zip › raw data-2/Figure 6/Fingure6 D-I/Fingure6 G/241006_Assay_2.jpg]

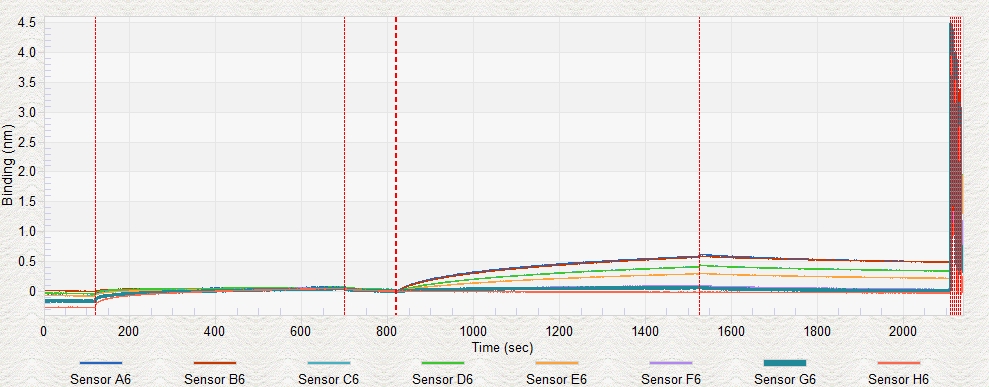

Supplement: Supplementary file 8 [file DataSheet2.zip › raw data-2/Figure 6/Fingure6 D-I/Fingure6 H/241006_Assay_1.jpg]

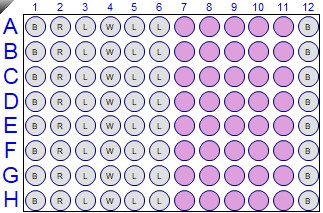

Supplement: Supplementary file 8 [file DataSheet2.zip › raw data-2/Figure 6/Fingure6 D-I/Fingure6 H/Plate1Definition.jpg]

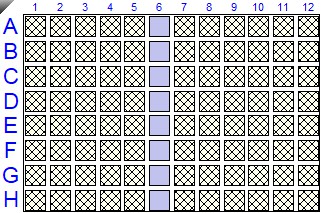

Supplement: Supplementary file 8 [file DataSheet2.zip › raw data-2/Figure 6/Fingure6 D-I/Fingure6 H/SensorPlate.jpg]

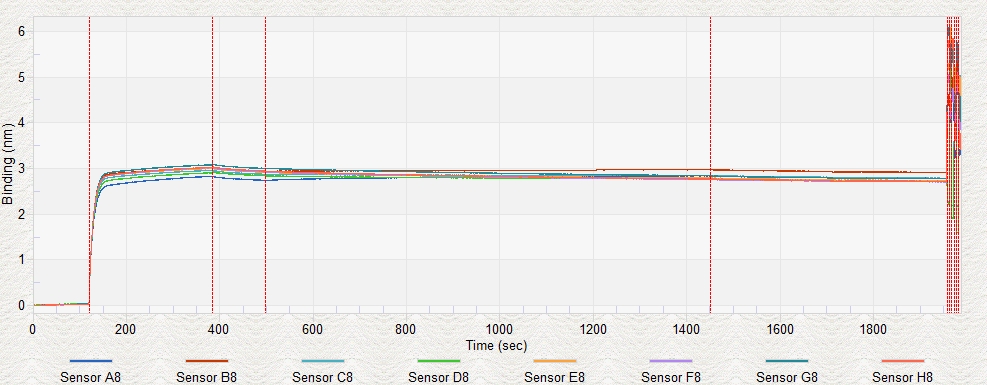

Supplement: Supplementary file 8 [file DataSheet2.zip › raw data-2/Figure 6/Fingure6 D-I/Fingure6 I/241006_Assay_1.jpg]

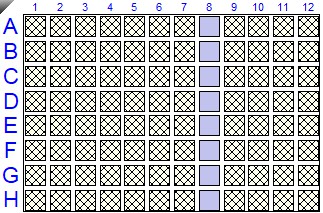

Supplement: Supplementary file 8 [file DataSheet2.zip › raw data-2/Figure 6/Fingure6 D-I/Fingure6 I/SensorPlate.jpg]

## Slide 1
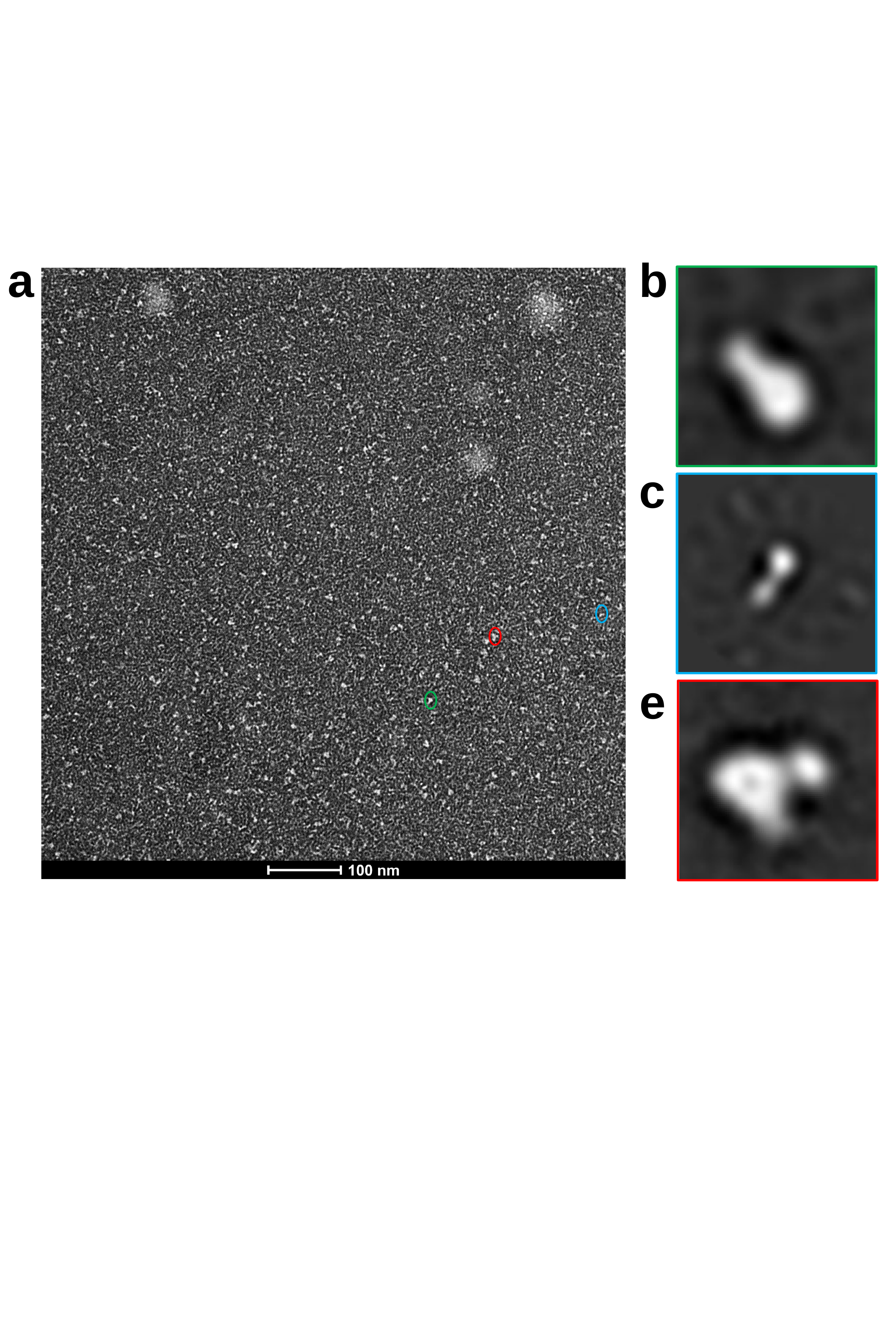

a
b
c
e

Supplement: Supplementary file 8 [file DataSheet2.zip › raw data-2/Figure 6/Fingure6 K.pptx]

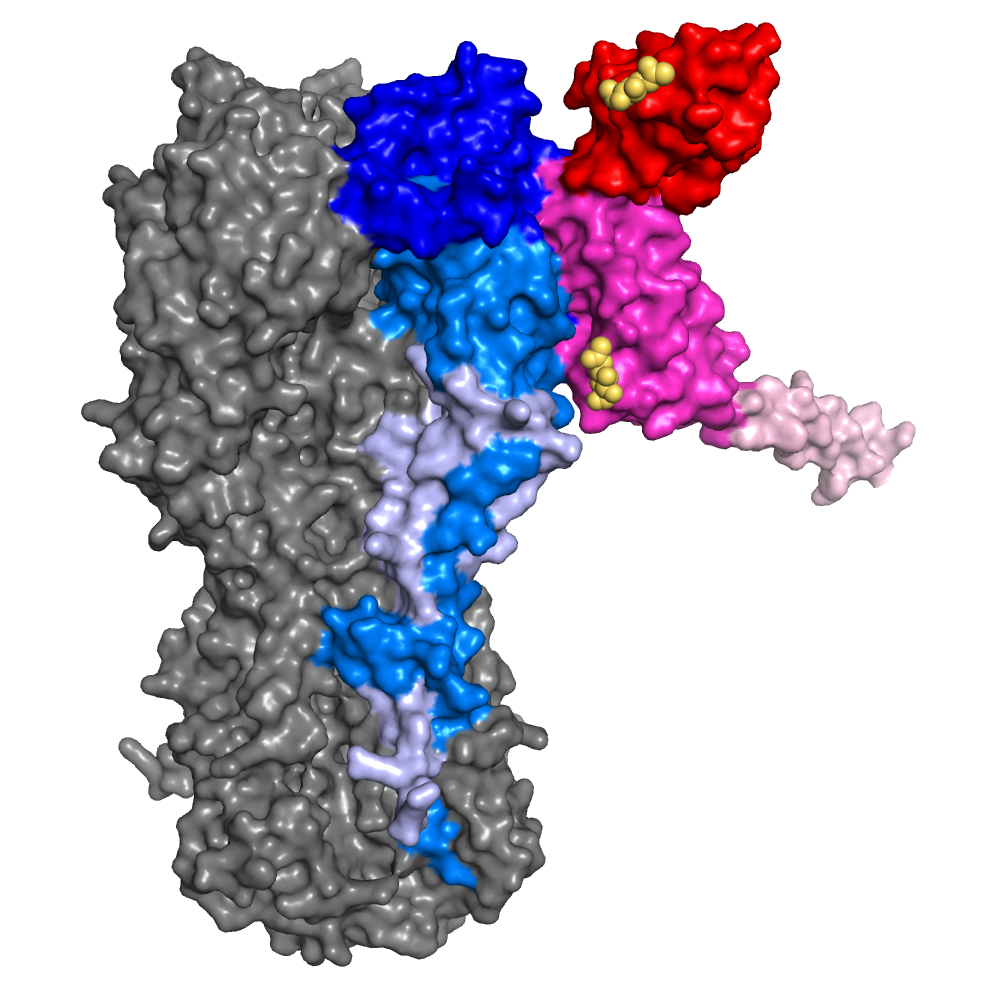

Supplement: Supplementary file 8 [file DataSheet2.zip › raw data-2/Figure 7/Fingure7 A B D E/7-A-1.png]

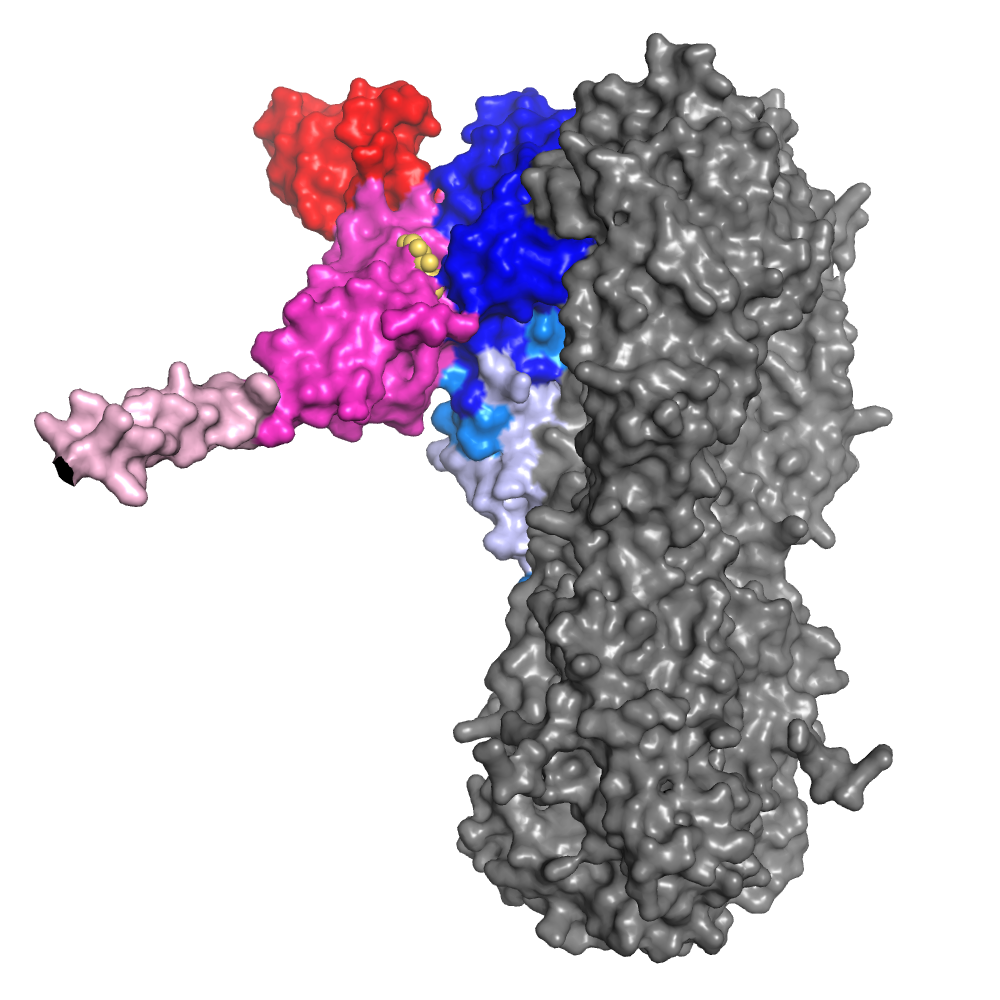

Supplement: Supplementary file 8 [file DataSheet2.zip › raw data-2/Figure 7/Fingure7 A B D E/7-A-2.png]

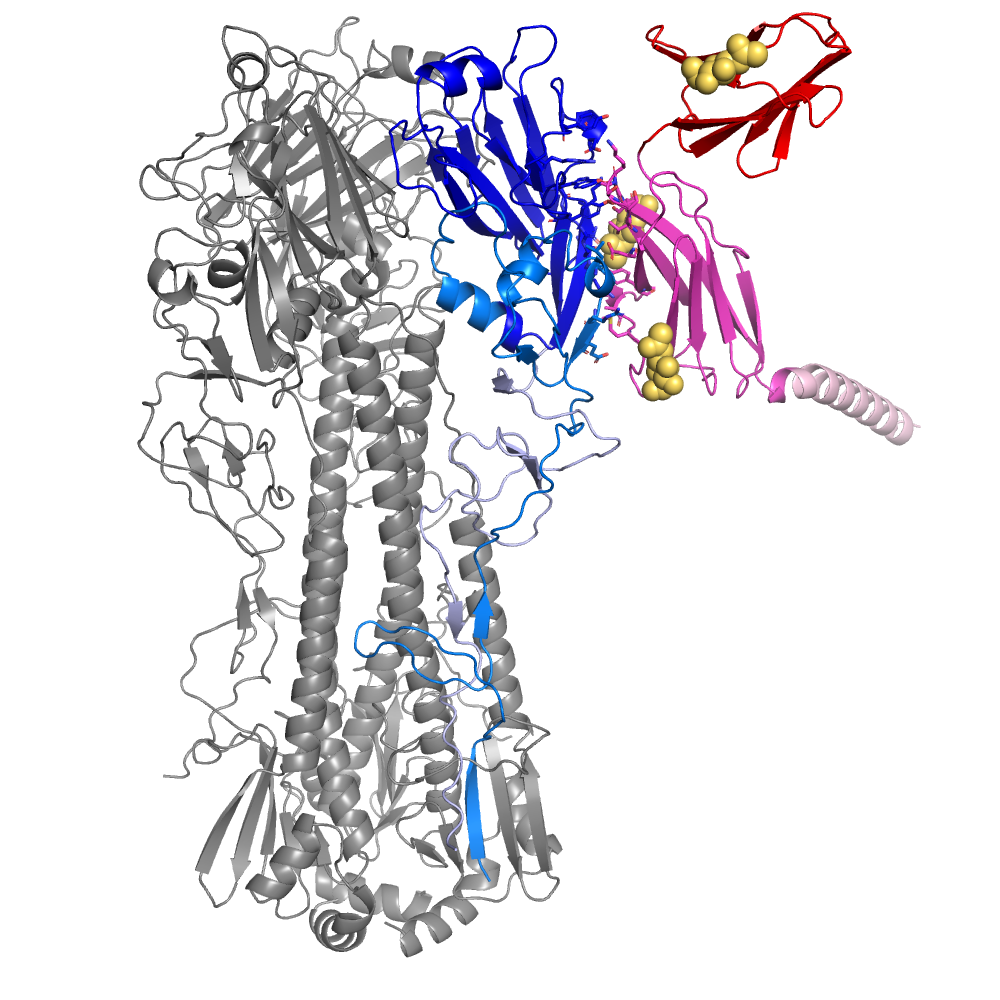

Supplement: Supplementary file 8 [file DataSheet2.zip › raw data-2/Figure 7/Fingure7 A B D E/7-B.png]

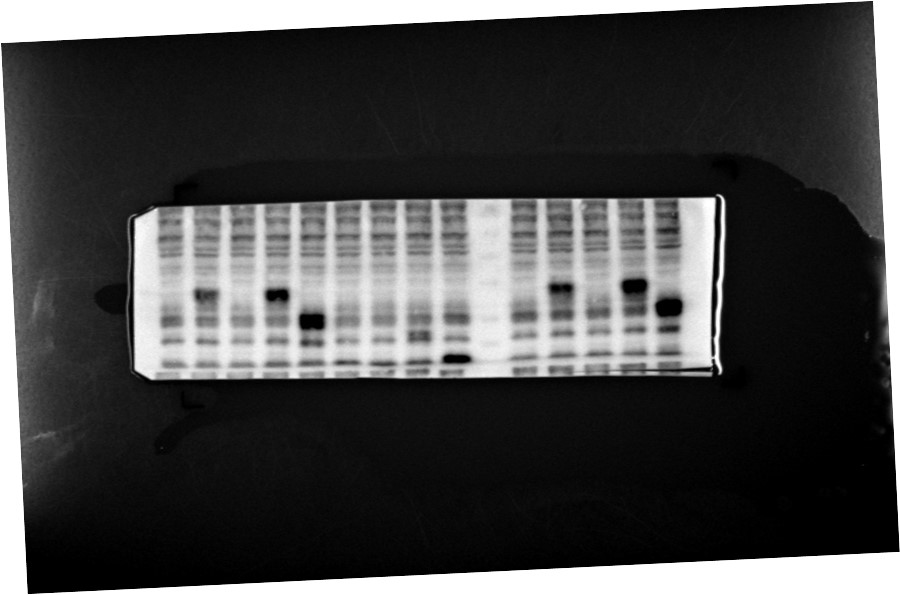

Supplement: Supplementary file 8 [file DataSheet2.zip › raw data-2/Figure 7/Fingure7 F/Input Flag.jpg]

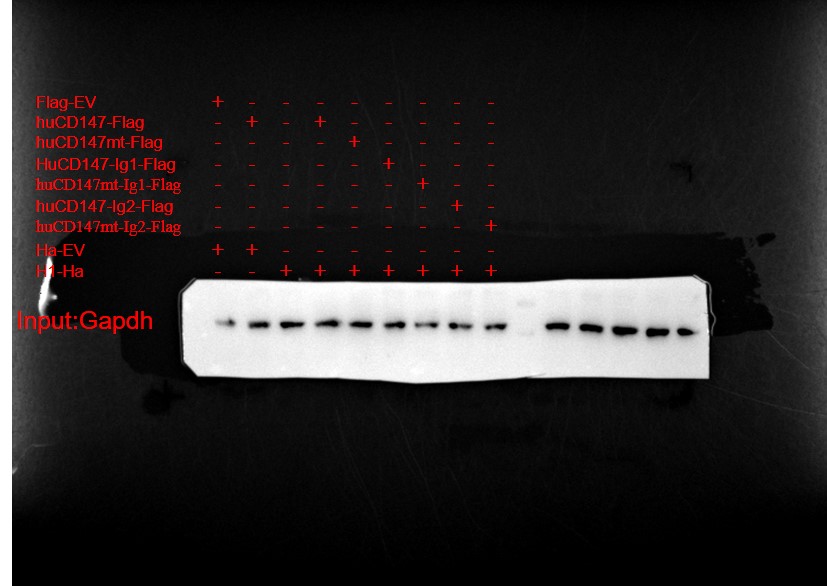

Supplement: Supplementary file 8 [file DataSheet2.zip › raw data-2/Figure 7/Fingure7 F/Input Gapdh.jpg]

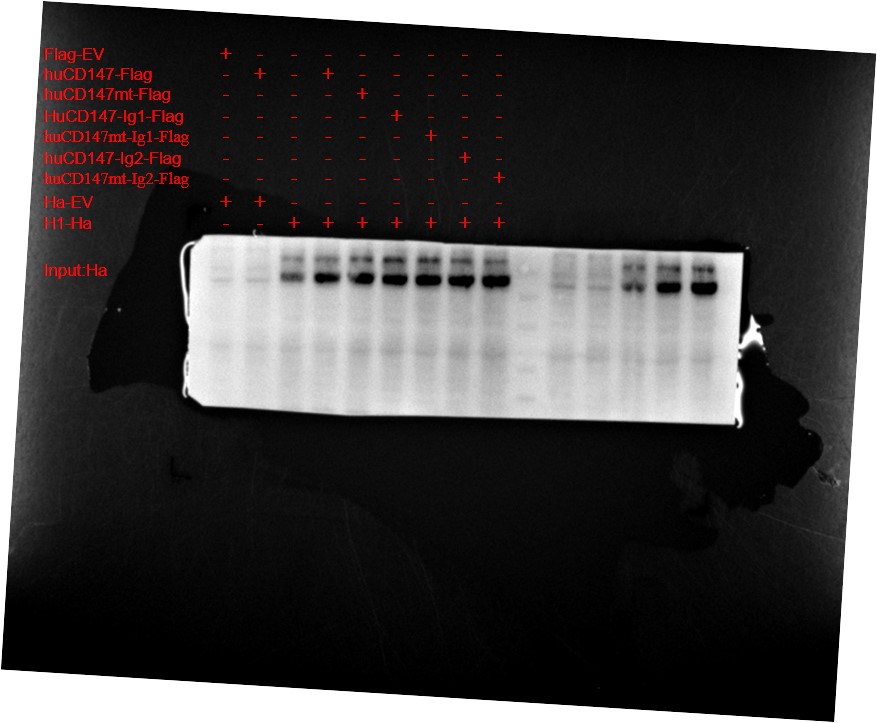

Supplement: Supplementary file 8 [file DataSheet2.zip › raw data-2/Figure 7/Fingure7 F/Input Ha.jpg]
